# PCPE-1 promotes cardiac fibrosis with aging and obesity

**DOI:** 10.1172/jci.insight.195508

**Published:** 2026-04-09

**Authors:** Yung-Ting Hsiao, Yohko Yoshida, Hirotsugu Tsuchimochi, Jingyuan Tang, Tin May Aung, Chun-Han Chang, Agian Jeffilano Barinda, Zhihong Li, Nur Syakirah Binti Othman, Tom Yoshizaki, Yiwei Ling, Shujiro Okuda, Manabu Abe, Seiya Mizuno, Satoru Takahashi, Takayuki Inomata, Hidetaka Kioka, Yasushi Sakata, Daichi Maeda, Yuya Matsue, Takaaki Furihata, Hiroshi Iwata, James T. Pearson, Kinya Otsu, Kenneth Walsh, Akihito Ishigami, Tohru Minamino, Ippei Shimizu

**Affiliations:** 1 Department of Cardiovascular Aging, National Cerebral and Cardiovascular Center Research Institute, Osaka, Japan.; 2 Department of Cardiovascular Biology and Medicine, and; 3 Department of Advanced Senotherapeutics, Juntendo University Graduate School of Medicine, Tokyo, Japan.; 4 Department of Cardiac Physiology, National Cerebral and Cardiovascular Center Research Institute, Osaka, Japan.; 5 Department of Cardiovascular Pathophysiology and Therapeutics, The University of Osaka Graduate School of Medicine, Osaka, Japan.; 6 Division of Bioinformatics, Niigata University Graduate School of Medical and Dental Sciences, Niigata, Japan.; 7 Department of Cellular Neurobiology, Brain Research Institute, and; 8 Department of Animal Model Development, Brain Research Institute, Niigata University, Niigata, Japan.; 9 Laboratory Animal Resource Center in Transborder Medical Research Center, Institute of Medicine, University of Tsukuba, Ibaraki, Japan.; 10 Department of Cardiovascular Medicine, Niigata University Graduate School of Medical and Dental Sciences, Niigata, Japan.; 11 Department of Cardiovascular Medicine, The University of Osaka Graduate School of Medicine, Osaka, Japan.; 12 National Cerebral and Cardiovascular Center Research Institute, Osaka, Japan.; 13 Division of Cardiovascular Medicine, Robert M. Berne Cardiovascular Research Center, University of Virginia School of Medicine, Charlottesville, Virginia, USA.; 14 Molecular Regulation of Aging, Tokyo Metropolitan Institute for Geriatrics and Gerontology, Tokyo, Japan.; 15 Department of Cardiovascular Medicine, National Cerebral and Cardiovascular Center, Osaka, Japan.

**Keywords:** Aging, Cardiology, Adipose tissue

## Abstract

Heart failure with preserved ejection fraction (HFpEF) is a multifactorial disease that develops in several clinical settings. Despite its complex pathogenesis, evidence indicates a central role for fibrosis in the progression of left ventricular diastolic dysfunction (LVDD). Through exploratory research into adipokines derived from brown adipose tissue (BAT), we identified a secreted-type profibrotic protein, procollagen C-endopeptidase enhancer-1 (PCPE-1), whose expression increased in BAT with aging. PCPE-1 promotes the cleavage of procollagens and is a critical initiator of fibrillogenesis. This molecule was increased in the plasma of aged mice. In addition to aging, obesity led to an increase in PCPE-1 expression in the LV of mice. Both systemic and BAT-specific PCPE-1 depletion ameliorated LV fibrosis and LVDD in the obese HFpEF model. Our data also showed that age-associated LVDD was ameliorated in the systemic PCPE-1–KO mouse fed with a normal chow diet. Conversely, the overexpression of PCPE-1 expression in BAT was shown to lead to aggravation of LV fibrosis and LVDD. Mechanistically, we found ROS/DNA damage/c-Fos/c-Jun signaling resulted in an increased production of PCPE-1 in brown adipocytes. These results indicate PCPE-1 may represent a druggable target for aging- and obesity-related HFpEF.

## Abstract 2

We found that PCPE-1, produced predominantly from the brown adipose tissue, promoted cardiac fibrosis with aging and obesity.

## Introduction

Heart failure is a life-threatening disease reported to affect more than 64 million individuals worldwide (1, 2). Heart failure with preserved ejection fraction (HFpEF) is defined as heart failure with a left ventricular ejection fraction (LVEF) of 50% or higher at diagnosis and accounts for approximately half of all patients with heart failure. The prevalence of HFpEF is increasing, and it is associated with high hospitalization, rehospitalization, and mortality rates (3). Despite remarkable recent advances in heart failure with reduced ejection fraction (HFrEF) treatment (4–8), therapeutic options remain extremely limited for HFpEF. SGLT2 inhibitors are the only drug class with robust, dedicated outcome trials demonstrating reductions in heart-failure events (9, 10). More recently, GLP-1 receptor agonists and dual GIP/GLP-1 receptor agonists have also shown beneficial effects — including reductions in worsening heart-failure events — in obesity-related HFpEF, suggesting an emerging second class of therapies with outcome-modifying potential (11–14). Thus, there is a critical need to establish additional therapeutic options for this challenging disorder, particularly given that HFpEF is a systemic disease affecting not only cardiac tissue but also multiple organs (15). Furthermore, its pathogenesis shows considerable heterogeneity in its morphological and functional features. Of these, left ventricular diastolic dysfunction (LVDD) (impaired relaxation, increased stiffness) is recognized as the most common phenotype of HFpEF (16), with accumulating evidence suggesting a central role for myocardial fibrosis in the development of myocardial stiffness and LVDD that drives the pathogenesis in HFpEF (17). However, it remains unclear whether suppression of cardiac fibrosis per se may represent a viable therapy for HFpEF. Thus, given the current limitations of treatments for HFpEF, we sought to explore mechanistic pathways to identify potential druggable targets for this disorder. The therapeutic potential of adipokines derived from white adipose tissue (WAT), including IL-1β and TNF-α, has been tested in clinical trials to combat cardiovascular disorders, resulting in inconsistent results depending on the disease or molecule targeted (18, 19). Unlike WAT-derived counterparts, brown adipose tissue–derived (BAT-derived) adipokines (BATokines) have not been sufficiently tested for their potential as therapeutic targets in cardiovascular diseases. Thus, our research focused on BAT research as a potential avenue for drug discovery for HFpEF. Procollagen C-endopeptidase enhancer-1 (PCPE-1) is a secreted extracellular matrix protein that enhances the cleavage of the C-pro-peptides of procollagen, thereby promoting the formation of mature, fibrillary collagen. Through exploratory research into BATokines, here we show that PCPE-1 promotes LV fibrosis and LVDD in the murine obesity or aged HFpEF models.

## Results

### Characterization of secreted factors from BAT.

**Figure 1:**
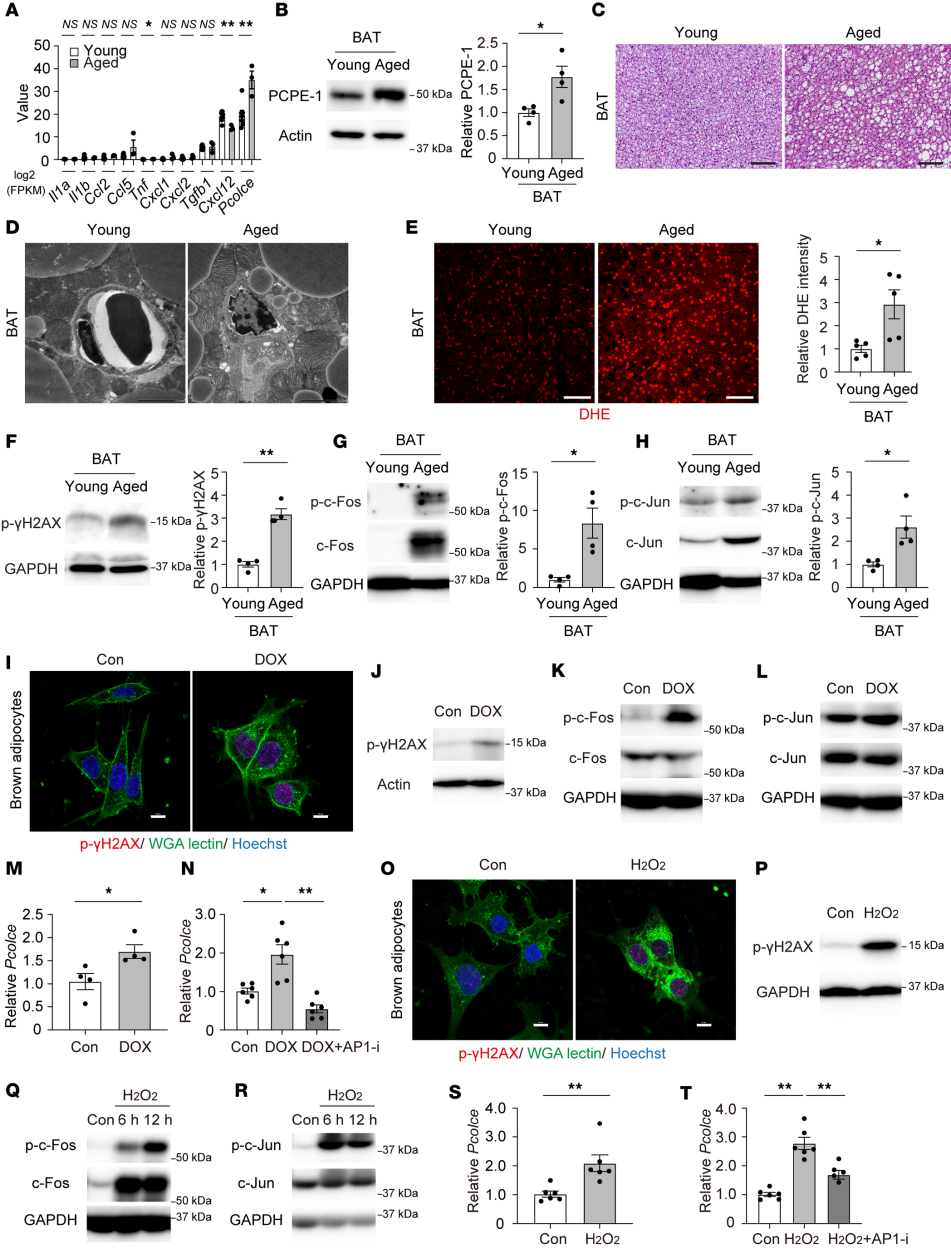
Characterization of secreted factors from BAT. BAT from C57BL/6NCrSlc mice fed an NCD were analyzed. (**A**) Panel studied young (12–13 weeks) or aged (79 weeks) mice, and other panels tested young (11 weeks) or aged (111–116 weeks) mice. (**A**) RNA-seq analyzing BAT (GSE274901) (*n* = 7, 3). (**B**) BAT PCPE-1 Western blot and quantification (*n* = 4, 4). (**C**) H&E staining of BAT. Scale bar: 100 μm. (**D** and **E**) Electron microscopic findings (**D**: Scale bar: 2 μm) and dihydroethidium staining of BAT (**E**: Scale bar: 50 μm) with quantification (*n* = 5, 5). (**F**–**H**) BAT Western blot analysis of phospho-γH2AX (**F**), phospho-cFos and c-Fos (**G**), phospho-c-Jun and c-Jun (**H**), and their quantification relative to GAPDH (*n* = 4, 4). (**I** and **O**) Immunofluorescent staining for phospho-γH2AX, WGA lectin, and Hoechst in differentiated brown adipocytes administered with DOX (**I**) or H_2_O_2_ (**O**). Scale bar: 10 μm. (**J**–**L** and **P**–**R**) For differentiated brown adipocytes, Western blot of phospho-γH2AX (**J** and **P**), phospho-cFos and c-Fos (**K**, **L**, **Q**, and **R**). (**M**, **N**, **S**, and **T**) qPCR showing transcript *Pcolce* in differentiated brown adipocytes, administered DOX (**M** [*n* = 4], **N** [*n* = 6]) or H_2_O_2_ (**S** and **T**) (*n* = 6), together with an AP1 inhibitor (AP1-i) (**N** and **T**). Housekeeping genes: *Actb* (**M**) and *18s* (**N**, **S**, and **T**). All data, except for **N** and **T** (2-way ANOVA followed by Dunnett’s T3 for **N**; Tukey’s multiple-comparison test for **T**), were analyzed by an independent-samples *t* test. Data information: Representative H&E-stained images or electron microscope images from 1 series of observations (**C** and **D**), and other data were obtained from 1 representative analytical experiment out of at least 2 independent experiments showing similar results. *P < 0.05, **P < 0.01. Values are presented as mean ± SEM. NS = not significant.

In an exploratory analysis, we performed bulk RNA-seq on BAT collected from 3-month-old (young) and 19-month-old (aged) C57BL6/NcrSlc male mice fed with a normal chow diet (NCD). We found that transcript *Pcolce*, the gene encoding the secreted-type profibrotic protein PCPE-1, ranked among the top 4 molecules shown to be preferentially upregulated by aging in BAT (Table 1). This led us to examine transcripts for other well-known adipokines for their expression in aged BAT, which demonstrated that transcript *Tnf* was mildly increased with aging; however, the expression level change of this molecule was lower than that of *Pcolce* (Figure 1A). We also found that transcripts for other traditional adipokines, including *Il1a*, *Il1b*, *Ccl2*, *Ccl5*, *Cxcl1*, *Cxcl2*, and *Tgfb1*, were comparable in expression level between young and aged mice, while that for *Cxcl12* was reduced in expression with aging (Figure 1A). In addition to the *Pcolce* transcript, we found that aged BAT displayed high-level expression of the protein PCPE-1 (Figure 1B). Analysis of single-cell RNA-seq data deposited in the Tabula Muris Senis (https://tabula-muris-senis.sf.czbiohub.org/) showed that transcript *Pcolce* was predominantly expressed in specific organs, such as adipose tissue, aorta, and heart (Supplemental Figure 1A; supplemental material available online with this article; https://doi.org/10.1172/jci.insight.195508DS1). In BAT, this analysis revealed that mesenchymal stem cells from adipose tissue had an enriched profile for *Pcolce* expression (Supplemental Figure 1B), and this was higher compared with fibroblasts in the heart (Supplemental Figure 1C). We collected preadipocytes from BAT of neonatal rats and found that their differentiation led to increased *Pcolce* transcript (Supplemental Figure 1D), and this level was higher compared with cardiac fibroblasts collected from rat neonates (Supplemental Figure 1E). PCPE-1 is an extracellular matrix protein that enhances the cleavage of the C-pro-peptides of procollagen, and this molecule is not considered to mediate biological effects targeting cells. In line with this concept, administration of recombinant PCPE-1 protein did not affect transcripts involved in fibrosis in cultured neonatal rat ventricular myocytes and fibroblasts (Supplemental Figure 1F). Supplementing cultures with this protein also did not change the level of an activation marker in cardiac fibroblasts (Supplemental Figure 1G). Quantitative PCR (qPCR) studies indicated that the transcript *Pcolce* was reduced in the heart, aorta, and skeletal muscle with age (Supplemental Figure 1H). In contrast, *Pcolce* expression increased with age in BAT and liver (Supplemental Figure 1H), and the analysis of transcript levels suggested that BAT was the main source of its production with aging (Supplemental Figure 1I). In aging mice, H&E staining showed mild enlargement of intracellular lipids in brown adipocytes (Figure 1C and Supplemental Figure 1J), and an electron microscope study revealed a ballooning phenotype of mitochondria together with a diminished cristae formation within this organelle (Figure 1D and Supplemental Figure 2A). Dihydroethidium staining showed a significant increase in ROS in aged BAT (Figure 1E), together with an increase in oxidative stress–related DNA damage marker, 8-hydroxy-2’-deoxyguanosine (8-OHdG) (Supplemental Figure 2, B and C). Of the anti-ROS molecules evaluated, transcriptome studies showed the highest expression of *Gpx3* (coding gene for glutathione peroxidase 3). However, this transcript declined in BAT with aging (Supplemental Figure 2D). Other anti- or pro-ROS markers showed varying results depending on the molecule studied (Supplemental Figure 2D). In this setting, phospho-γH2AX and the expression of DNA damage–related genes *Parp3* and *Gadd45a* increased in the fat pad (Figure 1F and Supplemental Figure 2E), as did its downstream molecules, including c-Fos and c-Jun (Figure 1, G and H). Analysis of the Tabula Muris single-cell RNA-seq data revealed that the cells annotated as adipose tissue mesenchymal stem cells exhibited high *Pcolce*, *Fos*, and *Jun* expression (Supplemental Figure 2F). To further test the role of DNA damage/c-Fos/c-Jun pathway, doxorubicin (DOX) was administered to differentiated brown adipocytes. We found that DOX induced DNA damage (Figure 1, I and J, and Supplemental Figure 2, G–I) and resulted in an increase in phosphorylated c-Fos and c-Jun levels in these cells (Figure 1, K and L, and Supplemental Figure 2, J and K). We also found that DOX increased the expression of the *Pcolce* transcript, which was abolished by the administration of a c-Fos/c-Jun complex (AP-1) inhibitor (Figure 1, M and N). To further test the role of ROS in the initiation of DNA damage, H_2_O_2_ was administered to differentiated brown adipocytes. We found that H_2_O_2_ administration led to an increase in the level of the DNA damage marker, as well as the phosphorylation of c-Fos and c-Jun levels (Figure 1, O–R, and Supplemental Figure 3, A–E). H_2_O_2_ also increased the *Pcolce* transcript, which was suppressed by treatment with the AP-1 inhibitor (Figure 1, S and T). Although we previously found that the ER stress–mediated IRE1/JNK/c-Fos/c-Jun pathway increased the PCPE-1 level in obesity (20), the level of phospho-IRE1 remained low in the aged BAT compared with BAT collected from obese mice (Supplemental Figure 3F). Collectively, these results indicated that ROS/DNA damage/c-Fos/c-Jun signaling increased the production of PCPE-1 in brown adipocytes with aging.

### HFpEF models exhibit high PCPE-1 levels in hearts.

**Figure 2:**
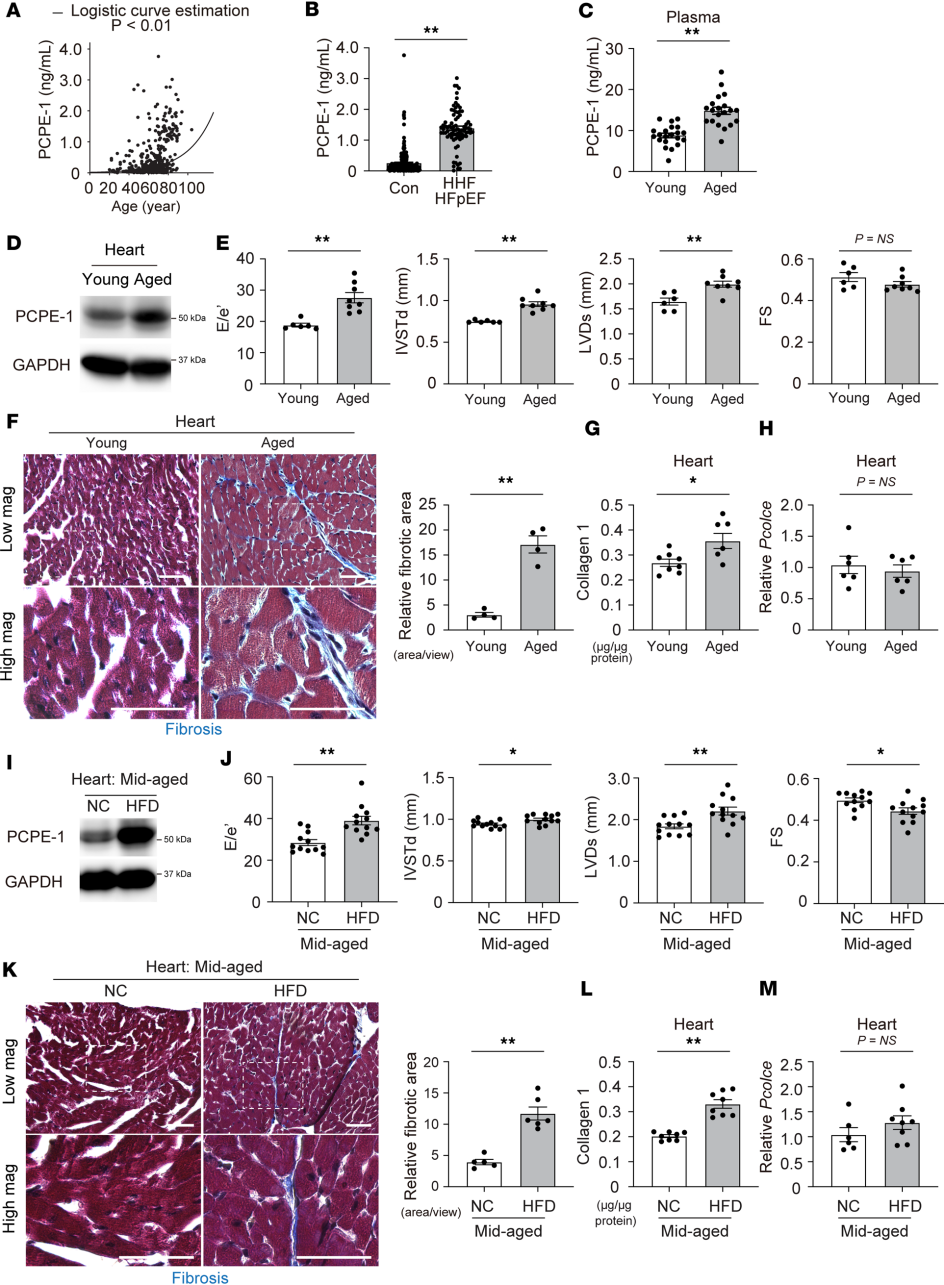
HFpEF models exhibit high PCPE-1 level in hearts. (**A** and **B**) Plasma PCPE-1 ELISA in the total cohort (*n* = 405) (**A**), and in patients (EF ≥ 50%) hospitalized with heart failure (HHF) (*n* = 80) or other diagnoses (Con) (*n* = 219) (**B**). (**C**) Plasma PCPE-1 ELISA in young (10–12 weeks) and aged (103–135 weeks) mice (*n* = 21, 20). (**D**) Cardiac PCPE-1 Western blot in young (10–12 weeks) and aged (103–135 weeks) mice. (**E**) Echocardiography in young (8 weeks) and aged (94 weeks) mice: E/e’ (marker for diastolic dysfunction), IVSTd (interventricular septum thickness), LVDs (left ventricular systolic dimension), FS (fractional shortening) (*n* = 6, 8). (**F**) Masson’s trichrome stain of the heart (Scale bar: 50 μm) and its quantification (% area/view) (*n* = 4, 4). (**G** and **H**) ELISA for collagen type I (shown as Collagen 1) (**G**) (*n* = 8, 7) or transcript *Pcolce* (**H**) (*n* = 6, 6) in the heart of young (10–12 weeks) and aged mice (103–135 weeks). Housekeeping gene: *Actb*. (**I**–**M**) These panels studied C57BL/6NCrSlc mice fed with a normal chow diet (NCD) or a high-fat diet (HFD) aged 44–45 weeks (middle-aged). (**I**) Cardiac PCPE-1 Western blot. (**J**) Echocardiography in mice (*n* = 12, 12). (**K**) Cardiac Masson’s trichrome stain and quantification (*n* = 5, 6). Scale bar: 50 μm. (**L** and **M**) ELISA for Collagen 1 (**L**) (*n* = 8, 8) or transcript *Pcolce* (**M**) (*n* = 6, 8) in the hearts. Housekeeping gene: Rplp0. Logistic curve estimation in **A**; other panels: independent-samples *t* test. Data information: Representative Masson’s trichrome–stained images from 1 series of observations (**F** and **K**); other data were obtained from 1 representative analytical experiment out of at least 2 independent experiments showing similar results. *P < 0.05, **P < 0.01. Values are presented as mean ± SEM. NS = not significant.

Next, we characterized the protein levels of PCPE-1 in humans and found that circulating PCPE-1 increased with age (Figure 2A), and among them, patients hospitalized with heart failure and diagnosed as HFpEF had high levels of this secreted-type profibrotic protein compared with all other cases exhibiting ejection fraction of 50% or greater (Figure 2B). The level of PCPE-1 did not show a positive correlation with BMI (Supplemental Figure 4A). Patients with hypertension had higher circulating PCPE-1 concentrations (Supplemental Figure 4B); however, levels were similar in patients with type 2 diabetes or dyslipidemia (Supplemental Figure 4, C and D). In multiple regression analyses, we found that age, but not hypertension, independently correlated with circulating PCPE-1 (Supplemental Table 1). PCPE-1 also increased in the plasma and heart of aged mice (Figure 2, C and D, and Supplemental Figure 4E) and was associated with LVDD and LV hypertrophy (Figure 2E). Although we found that body and heart weight increased with age (Supplemental Figure 4, F–H) and the aged hearts became fibrotic (Figure 2, F and G, and Supplemental Figure 4I), the transcript levels of *Pcolce* in the heart were comparable between young and aged mice (Figure 2H). We next characterized middle-aged mice fed with a high-fat diet (HFD) versus an NCD. The HFD increased both body and heart weight (Supplemental Figure 4, J–L), and this condition increased the expression of the DNA damage marker in BAT (Supplemental Figure 4M). In this setting, it was found that PCPE-1 protein levels were increased in the heart (Figure 2I and Supplemental Figure 4N). Echocardiography indicated that LVDD and LV hypertrophy also developed in the HFD condition (Figure 2J). Although histological analysis, biochemical quantification of hydroxyproline, and ELISA findings showed that the heart became fibrotic (Figure 2, K and L, and Supplemental Figure 4O), the cardiac *Pcolce* transcript was comparable between the HFD and NCD groups (Figure 2M).

### Depletion of PCPE-1 ameliorates HFpEF in obesity and aged mouse models.

**Figure 3:**
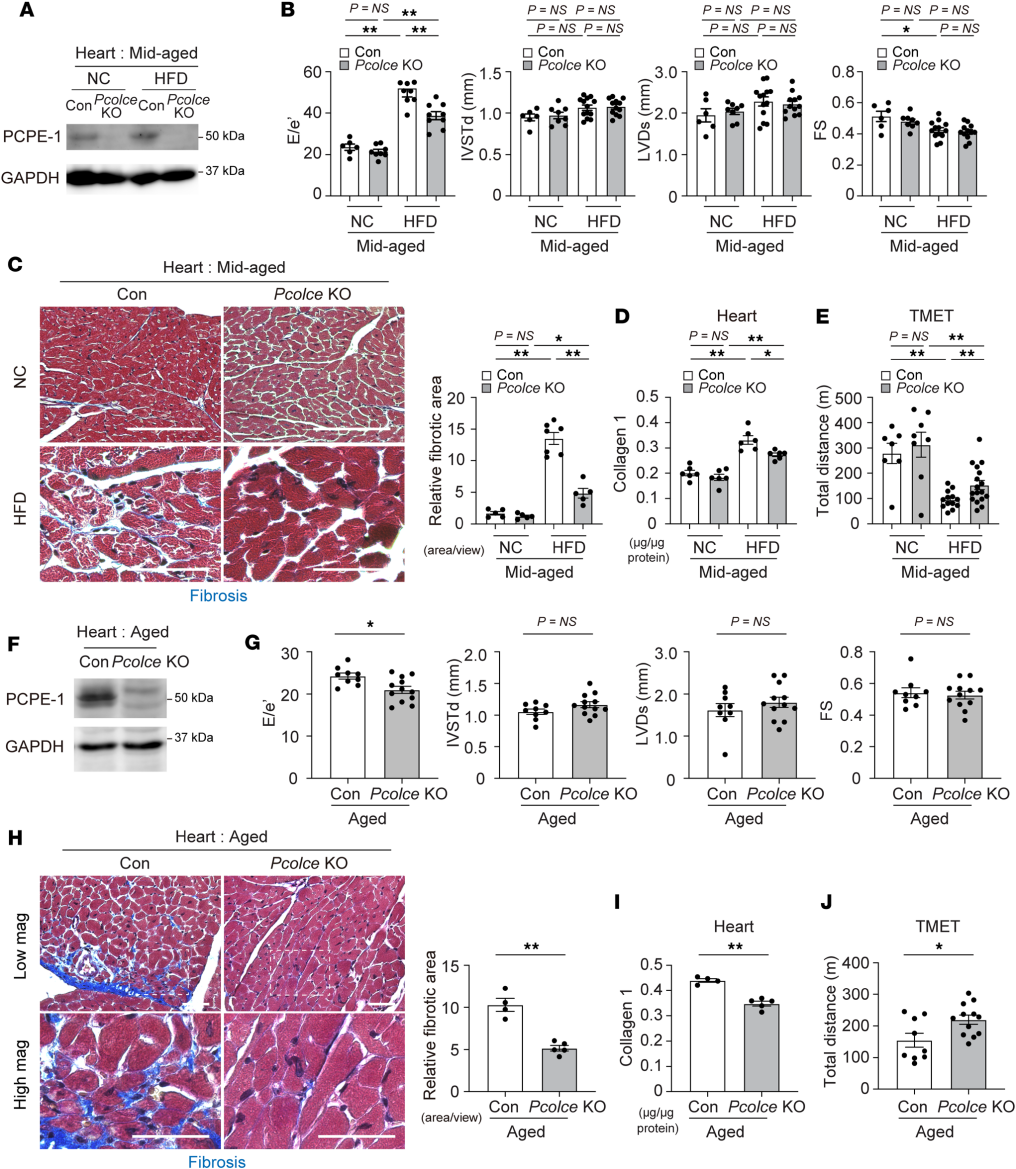
Depletion of PCPE-1 ameliorates HFpEF in obesity and aged mouse models. (**A**–**E**) Littermate control (Con) or systemic *Pcolce*-KO mice fed with a high-fat diet (HFD) from 4 weeks of age or a normal chow diet (NCD) and studied at 41–46 weeks of age (middle-aged). (**F**–**J**) Systemic *Pcolce*-KO mice (74–80 wk) fed with an NCD. (**A** and **F**) Western blot analysis of PCPE-1 expression in the heart. (**B** and **G**) Echocardiographic findings in the indicated mice: E/e’ (marker for diastolic dysfunction), IVSTd (interventricular septum thickness), LVDs (left ventricular systolic dimension), FS (fractional shortening) (*n* = 6, 8, 8, 9 for E/e’ and *n* = 6, 8, 13, 12 for other parameters in **B**; *n* = 9, 12 for **G**). (**C** and **H**) Masson’s trichrome stain of the heart. Scale bar: 50 μm. Right panels indicate quantification of relative fibrotic area (**C**) (*n* = 7, 5) (**H**) (*n* = 4, 5). (**D** and **I**) ELISA for cardiac collagen type I (shown as Collagen 1) (*n* = 10, 9 for **D**) and (*n* = 4, 5 for **I**). (**E** and **J**) Treadmill examination test (TMET) showing the running distance (*n* = 13, 17 for **E**) and (*n* = 9, 12 for **J**). Two-way ANOVA followed by Tukey’s multiple-comparison (**B** and **D**) or Dunnett T3 multiple comparison test (**C**); others: independent-samples *t* test. Data information: Representative Masson’s trichrome–stained images from 1 series of observations (**C** and **H**), and other data were obtained from 1 representative analytical experiment out of at least 2 independent experiments showing similar results. *P < 0.05, **P < 0.01. Values are presented as mean ± SEM. NS = not significant.

Next, we characterized systemic middle-aged *Pcolce*-KO (*Pcolce^–/–^*) mice maintained on an NCD or an HFD (Figure 3A and Supplemental Figure 5A). This analysis revealed that both body and heart weight were comparable between genotypes on the different diet regimens (Supplemental Figure 5, B–D). In this setting, the levels of systemic glucose and insulin intolerance were similar between the genotypes in the HFD-fed groups (Supplemental Figure 5, E and F), but we found that PCPE-1 deficiency mitigated LVDD under obesity (Figure 3B and Supplemental Figure 5G) and diminished the degree of cardiac fibrosis (Figure 3, C and D, and Supplemental Figure 5H). Physiological studies also showed an increase in physical activity among the HFD-fed middle-aged *Pcolce*-KO mice (Figure 3E).

In the aging model, the genetic depletion of PCPE-1 resulted in the amelioration of age-associated LVDD (Figure 3, F and G, and Supplemental Figure 6A). Similar to the model of obesity, there were no differences in body weight and heart weight between the genotypes (Supplemental Figure 6, B–D); however, the level of LV fibrosis was diminished in the aged KO mice compared with that in the littermate control group (Figure 3, H and I, and Supplemental Figure 6E). The running distance during the treadmill test also lengthened in the KO group (Figure 3J), and the degrees of systemic insulin and glucose tolerance were found to be comparable between the 2 genotypes (Supplemental Figure 6, F and G). We also found that LV-diastolic dysfunction was significantly ameliorated in the KO group of female mice, suggesting that PCPE-1 mediates the pathogenesis of age-related HFpEF in a sex-independent manner (Supplemental Figure 6H). Transcripts for fibrogenic mediators, including *Tgfb1, Tnf, Lgals3, Edn1*, and *Agt,* were comparable in the kidney of C57BL/6NCrSlc-WT mice fed an NCD or HFD (Supplemental Figure 6I), but the *Ccn2* transcript increased under this setting (Supplemental Figure 6J). In the HFD-fed *Pcolce*-KO male mice, transcripts for *Ccn2* were reduced compared with HFD-fed littermate control male mice (Supplemental Figure 6, K and L), but there was no detectable effect on the transcripts of other profibrogenic mediators.

### The BATokine PCPE-1 contributes to the pathogenesis of HFpEF.

**Figure 4:**
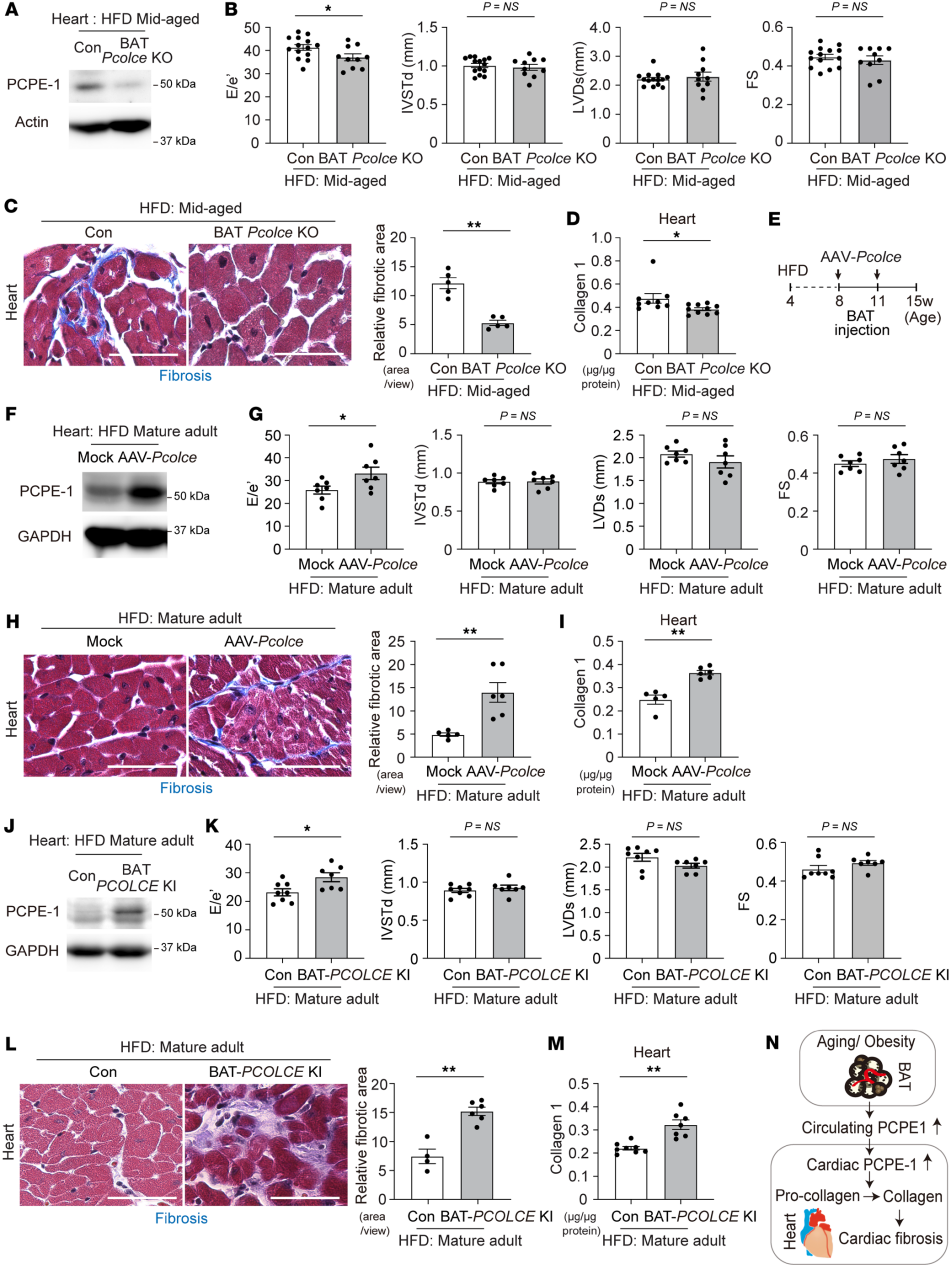
The BATokine PCPE-1 contributes to the pathogenesis of HFpEF. Littermate control (Con) or BAT *Pcolce*-KO mice were fed with a high-fat diet (HFD) and studied during 41–47 weeks of age (middle-aged). BAT adeno-associated virus (AAV) encoding *Pcolce* was injected in the BAT of C57BL/6NCrSlc mice fed with an HFD and studied at 15 weeks of age (mature adult). BAT-*PCOLCE* KI mice were also fed with an HFD and studied at 23–25 weeks of age (mature adult). (**A**, **F**, and **J**) Western blot analysis of mouse (**A** and **F**) or human (**J**) PCPE-1 expression in the heart of the indicated mice. (**B**, **G**, and **K**) Echocardiographic findings in mice: E/e’ (marker for diastolic dysfunction), IVSTd (interventricular septum thickness), LVDs (left ventricular systolic dimension), FS (fractional shortening) (*n* = 14, 10 for **B**), (*n* =7, 7 for **G**), and (*n* = 8, 7 for **K**). (**C**, **H**, and **L**) Masson’s trichrome stain of the heart and its quantification (*n* =5, 5 for **C**), (*n* = 5, 6 for **H**), and (*n* = 4, 6 for **L**) in mice. Scale bar: 50 μm. (**D**, **I**, and **M**) ELISA for collagen type I (shown as Collagen 1) testing hearts in mice (*n* = 9, 10 for **D**), (*n* = 5, 6 for **I**), and (*n* = 8, 7 for **M**). (**E**) Experimental design of the AAV-*Pcolce* mouse. (**N**) Schematic demonstrating that ROS/DNA damage/c-Fos/c-Jun signaling upregulates PCPE-1 expression in BAT with aging. PCPE-1 produced from BAT promotes fibrosis in the heart. All data were analyzed by an independent-samples *t* test. Data information: Representative Masson’s trichrome–stained images from 1 series of observations (**C**, **H**, and **L**), and other data were obtained from 1 representative analytical experiment out of at least 2 independent experiments showing similar results. *P < 0.05, **P < 0.01. Values are presented as mean ± SEM. NS = not significant.

To evaluate the contribution of BAT-derived PCPE-1 to the pathogenesis of HFpEF, we specifically depleted PCPE-1 in BAT by crossing UCP-1Cre^+/–^ with *Pcolce^fl/fl^* mice (i.e., BAT *Pcolce*-KO). In these mice, the levels of PCPE-1 in the plasma and heart were reduced compared with levels in the *Pcolce^fl/fl^* littermate control mice fed an HFD (Figure 4A and Supplemental Figure 7, A and B). Body, heart weight, levels of systemic glucose/insulin tolerance, and volumes of visceral and subcutaneous fat analyzed by CT were comparable between the genotypes (Supplemental Figure 7, C–H). Similar to the systemic *Pcolce*-KO mice, the *Ccn2* transcript was reduced in the kidney of the BAT *Pcolce*-KO mice under diet-induced obesity (Supplemental Figure 7I). It was also found that LVDD was ameliorated in BAT-specific *Pcolce*-KO of the middle-aged mice with obesity (Figure 4B), as was cardiac fibrosis (Figure 4, C and D, and Supplemental Figure 7J). We next generated a BAT-specific PCPE-1 overexpression model by the injection of adeno-associated virus (AAV) encoding *Pcolce* (AAV-*Pcolce*) into the BAT fat pad of adult mice (Figure 4E). This led to the induction of *Pcolce* expression in BAT and an increase in PCPE-1 levels in the heart under conditions of diet-induced obesity (Figure 4F and Supplemental Figure 8A). Body and heart weight were comparable between the groups (Supplemental Figure 8, B–D). Administration of AAV-*Pcolce* to BAT led to a deterioration of LVDD (Figure 4G) and an aggravation of cardiac fibrosis (Figure 4, H and I, and Supplemental Figure 8E).

To further test the role of BAT-derived PCPE-1, we generated BAT-specific human *PCOLCE* knock-in (KI) mice by crossing UCP1-Cre^+/–^ with *PCOLCE^KI/KI^* mice (BAT-*PCOLCE*-KI) (Supplemental Figure 8F). Analyses of the adult mice revealed that human PCPE-1 protein expression could be detected in hearts of the BAT-*PCOLCE*-KI mice fed with an HFD (Figure 4J and Supplemental Figure 8G). Body and heart weight were comparable between the genotypes (Supplemental Figure 8, H–J), but echocardiographic analysis revealed that LVDD was enhanced in the KI mice (Figure 4K), as was the progression of LV fibrosis (Figure 4, L and M, and Supplemental Figure 8K).

## Discussion

BAT, once believed to be predominantly involved in heat production, has gained attention for its role in systemic metabolic regulation. Furthermore, emerging evidence suggests that BAT secretes adipokines that can influence various physiological processes (21). However, their role in cardiovascular diseases remains unclear. Through exploratory research into BATokines, we found that the secreted-type profibrotic protein PCPE-1 increases in this fat pad with aging in mice. PCPE-1 binds to the C-terminal pro-peptide of procollagen and drives enzymatic cleavage of procollagens, thereby enhancing collagen fibrillogenesis (22). Recently, it was shown that diet-induced obesity increased PCPE-1 level in BAT and circulation, leading to enhanced pathogenesis in metabolic dysfunction–associated steatotic liver disease (20). Our present study demonstrates that aged BAT exhibits elevated levels of ROS and DNA damage, and in vitro data indicated that the ROS/DNA damage/c-Fos/c-Jun pathway was responsible for the production of this secreted profibrotic protein in brown adipocytes. Adipokines have been extensively studied in inflamed WAT in obesity (23–25). Of these, TNF-α, IL-1β, and IL-6 have attracted attention as molecules that causally contribute to the state of chronic sterile inflammation (18, 19, 26, 27). Notably, the IL-1β inhibitor canakinumab is reported to reduce the need for hospitalization for heart failure (28). However, etanercept, a TNF-α antagonist, was not able to exert beneficial effects on the rate of death or hospitalization from heart failure among high-risk patients (18). These studies suggest that targeting WAT adipokines may have utility for the treatment of cardiovascular diseases. In contrast, few studies have focused on adipokines produced from BAT and tested their potential as therapeutic targets in age- or lifestyle-related diseases. Interestingly, among the well-known adipokines, our transcriptome studies indicate *Tnf* and *Pcolce* alone to be the only adipokines that are increased with aging in BAT, with the induction level of *Pcolce* exceeding *Tnf* in aged BAT. Analysis of single-cell RNA-seq data from the Tabula Muris Senis revealed that *Pcolce* was predominantly expressed by preadipocytes in BAT. Our genetic studies involving the BAT-specific depletion of *Pcolce* revealed BAT as the primary source of this secreted profibrotic protein. Furthermore, in addition to chronological aging, we found that obesity increased PCPE-1 protein levels in the heart. Further studies using 2 loss-of-function models and 2 gain-of-function models demonstrated that PCPE-1 aggravates LV fibrosis and diastolic dysfunction in the obese-HFpEF model. Importantly, analysis of human samples showed that PCPE-1 increased in plasma with aging and was positively correlated with diastolic dysfunction in patients with HFpEF. The estimation of BAT weight in humans ranges from 14 g to above 500 g (29–32), but it is relatively minimal compared with rodents. Thus, it is not clear which organ predominantly produces PCPE-1 in humans. However, our preclinical and clinical studies indicate that this molecule in circulation may enhance tissue fibrosis in systemic organs, including the heart, indicating the potential therapeutic utility of targeting PCPE-1. Various diseases, such as hypertrophic cardiomyopathy, Fabry disease, cardiac amyloidosis, cardiac sarcoidosis, myocarditis, and hemochromatosis, can give rise to the clinical syndrome of heart failure with normal systolic function, which Borlaug et al. has described as “HFpEF masqueraders” (15). HFpEF can also develop as a consequence of multiple pathogenic conditions, such as aging- and lifestyle-related diseases including obesity, diabetes, dyslipidemia, and hypertension. Thus, the pathology of HFpEF is multifactorial and it is unlikely to be treated by a one-size-fits-all approach (33). However, accumulating evidence indicates that LV fibrosis is a central feature among many etiologies of HFpEF (17). An endomyocardial biopsy study showed that 93% of patients with HFpEF develop cardiac fibrosis (34). Compared with the LV pressure overload model or myocardial infarction (35–38), our data indicate that LV fibrosis induced by obesity or aging was microscopic, suggesting that micro-fibrosis of the heart is sufficient to develop LVDD.

Excessive ROS is established as a potential driving force for the aging phenotype, and DNA damage is known to accrue in aged organs. Our results showed that the ROS/DNA damage/c-Fos/c-Jun pathway was responsible for PCPE-1 production in brown adipocytes. In mice, the BAT-specific PCPE-1 loss-of-function or gain-of-function models provided evidence indicating that myocardial PCPE-1 largely originates from BAT. Although it was previously not clear whether suppression of cardiac fibrosis per se could become a therapeutic approach for HFpEF, our results indicate that inhibition of PCPE-1 can suppress LV fibrosis and ameliorate LVDD, suggesting that fibrosis could be a druggable target for HFpEF. Similar to the “fantastic-four” therapeutic approach for HFrEF. (39), combination therapies will likely be necessary for the treatment of HFpEF, and further studies are needed to test the clinical relevance of PCPE-1 and establish the potential antifibrotic agents in combination therapies.

We performed pressure-volume loop studies in HFD-fed systemic *Pcolce*-KO male mice and found that effective arterial elastance, a marker of afterload, was comparable between genotypes (Supplemental Figure 5G). qPCR analyses in the kidney revealed that the expression of a fibrogenic mediator, *Ccn2* (connective tissue growth factor, CTGF), was significantly reduced both in HFD-fed systemic *Pcolce*-KO male mice and HFD-fed BAT-specific *Pcolce*-KO male mice compared with their respective littermate controls (Supplemental Figure 6L and Supplemental Figure 7I). These results raise the possibility that the reduction of *Ccn2* levels observed in both the BAT-specific and whole-body KO mice may contribute to the attenuation of cardiac fibrosis. Ccn2 is a well-established profibrotic factor in the kidney and heart, and its downregulation could synergistically enhance the antifibrotic effects mediated by the loss of PCPE-1. Thus, the improvement in cardiac fibrosis in *Pcolce*-deficient mice may not solely result from direct inhibition of PCPE-1–dependent pathways, but may also involve secondary effects through the suppression of *Ccn2*. Although the precise molecular link between PCPE-1 and Ccn2 remains to be clarified, it is conceivable that PCPE-1 may regulate cardiac remodeling via interorgan crosstalk or through yet-unknown fibrotic signaling pathways. In contrast to BAT, our data showed that the *Pcolce* transcript did not increase in the heart with aging and obesity. However, this does not exclude the possibility of posttranscriptional regulation of this molecule in the heart under these settings. Thus, further studies are warranted to dissect organ-to-organ crosstalk and delineate the diverse fibrotic pathways involved in HFpEF.

Taken together, our studies indicate the ROS/DNA damage/c-Fos/c-Jun pathway increases PCPE-1 production in BAT, thus augmenting cardiac fibrosis and diastolic dysfunction in the obese or aged HFpEF mouse models (Figure 4N). Given the high circulating PCPE-1 levels in patients with HFpEF, inhibition of this secreted profibrotic protein could prove a viable therapeutic option for HFpEF.

## Methods

### Sex as a biological variable.

Our study examined male mice, except for Supplemental Figure 6H, showing echocardiographic findings in aged systemic *Pcolce*-KO female mice fed an NCD. It is unknown whether the findings in HFD male mice are relevant to female mice.

### Human samples.

Blood samples and clinical data were collected from inpatients or outpatients as biobank samples at the department of cardiology in Niigata University Hospital or Juntendo University Hospital. The timing of sample collection was determined by the primary physician and was not standardized. All study participants provided written informed consent prior to participation in the biobank registration. Samples collected from 2013 to 2019 were used for further studies. Diagnosis of HFpEF was made by at least 2 cardiologists based on the following criteria: (a) symptoms and physical signs of heart failure requiring hospitalization and (b) echocardiographic findings demonstrating LVEF of 50% or greater. Plasma samples were subjected to ELISA, and the PCPE1 ELISA kit Human Procollagen C Proteinase Enhancer 1, 96-strip wells (Sandwich) were used according to the manufacturer’s instructions (MyBioSource, MBS722836S).

### Animal models.

Mice were housed in animal facilities under specific pathogen–free conditions at a constant temperature of 23°C and in a 12-hour light/12-hour dark cycle. We used the following mice from different age groups: young (8–13 wk), mature-adult (14–25 wk), middle-aged (41–47 wk), early-old (60–67 wk), and aged (78–135 wk) mice. All experiments included only male mice for analysis, except for Supplemental Figure 6H, which tested female mice. C57BL/6NCrSlc male mice were purchased from SLC Japan. Some of these mice were maintained on an HFD (calories 507.6 kcal/100 g; crude fat 32 g/100 g; crude protein 25.5 g/100 g; crude fiber 2.9 g/100 g) (HFD32, CLEA) or an NCD (calories 162.0 kcal/100 g; crude fat 4.76 g/100 g; crude protein 24.96 g/100 g; crude fiber 4.84 g/100 g) (CE-2, CLEA) from 4 weeks of age. Systemic *Pcolce-*KO or floxed *Pcolce* mice were generated by Abe et al. (20). To generate *Pcolce*-floxed mice, a targeting vector was constructed in the following steps. A 1.11 kb DNA fragment carrying exon 3 and exon 4 of the *Pcolce* was amplified by PCR and inserted into the KpnI sites of the middle entry clone (pDME-1). In this clone, a DNA fragment of pgk promoter-driven Neo-poly (A) (pgk-Neo) flanked by 2 frt sites and 1 loxP sequence was located at site 278 bp downstream of exon 4, while the other loxP sequence was placed at site 345 bp upstream of exon 3. The 6.97 kb upstream and 5.52 kb downstream homologous genomic DNA fragments were retrieved from the BAC clone, and then subcloned to 5′ entry clone (pD5UE-2) and 3′ entry clone (pD3DE-2), respectively. For the targeting vector assembly, the 3 entry clones were recombined to a destination vector plasmid (pDEST-DT) containing a cytomegalovirus enhancer/chicken actin (CAG) promoter-driven diphtheria toxin gene by using MultiSite Gateway Three-Fragment Vector construction kit (Thermo Fisher Scientific). The culture of embryonic stem cells and generation of chimeric mice were carried out as previously described (40). To generate *Pcolce*-deficient mice by CRISPR-Cas technology, hU6-sgRNA plasmids (41) were ligated with synthetic oligonucleotides of the sequence 5′- CTCCAGAGCATCGTAGCGGC -3′ and 5′- GGCACCTCAGGCCAGCGACT-3′, which are identical to part of exon 3 of Pcolce. C57BL6/N female mice were superovulated and mated with C57BL6/N males, and fertilized eggs were collected from the oviduct. The pronuclear stage eggs were injected with a plasmid expressing Cas9 nickase (System Bioscience) and hU6-sgRNA plasmids. UCP1-Cre^+/–^ (C57BL/6J background) were crossed with mice carrying floxed *Pcolce* alleles with a C57BL/6 background to generate BAT-specific PCPE-1–KO (UCP1-Cre^+/–^ *Pcolce^fl/fl^*) (referred to as BAT *Pcolce*-KO) mice. The genotypes of the littermate controls were UCP1-Cre^–/–^ *Pcolce^fl/fl^*. The UCP1-Cre^–/–^ mouse was provided by Evan Rosen (Beth Israel Deaconess Medical Center and Harvard Medical School, Boston, MA, USA). The PCPE–KI model was generated by Mizuno and Takahashi et al. (42). We selected a sequence (5′-CGCCCATCTTCTAGAAAGAC-3′) in the first intron of the *Gt(ROSA)26Sor* gene as the CRISPR target, and inserted this sequence into *pX330-mC* plasmid*,* which carried both guide RNA and Cas9-mC expression units (42). The donor DNA vector was *pRosa-CAG-fEGFP-hPCOLCE*, in which the previously reported mouse *Cables1* cDNA of *pRosa-CAG-fEGFP-Cables1* (42) was replaced with human *PCOLCE* cDNA. The above DNA vectors were microinjected into zygotes of C57BL/6J mice (Charles River Laboratories Japan; originally from The Jackson Laboratory, stock no. 000664) according to our previous report (42). Subsequently, microinjected zygotes were transferred into the oviducts of pseudo-pregnant Institute of Cancer Research (ICR) female mice (Crl:CD1(ICR), Charles River Laboratories Japan, strain code 022), and newborns were obtained.

To confirm the presence of the designed KI allele, the genomic DNA were purified from the tail with PI-200 (Kurabo Industries Ltd.) according to the manufacturer′s protocol. Genomic PCR was performed with KOD-Fx (Toyobo). The primers (ROSA screening 5 forward: 5′-TCTTTTCTGTTGGACCCTTACCTTGACC-3′ and ROSA screening 3 reverse: 5′-AATTTGATGGGGGAAAACTTGAATGAAA-3′) were used for detecting the correct KI allele. In addition, we checked random integration of DNA vectors by PCR with the donor vector backbone (VB) detecting primers (VB detection-forward 5′-GTTTTCCCAGTCACGACGTT-3′ and VB detection-reverse 5′-ACACGCCATTTGTGTTTTCA-3′) and Cas9 detecting primers (Cas9 detection forward 5′-AGTTCATCAAGCCCATCCTG-3′ and Cas9 detection reverse 5′-GAAGTTTCTGTTGGCGAAGC-3′). The homozygous *PCOLCE*-KI mice (*PCOLCE*^KI/KI^) were crossed with UCP1-Cre^+/–^ mice to generate BAT-specific PCPE-1–KI mice (UCP1-Cre^+/–^ *PCOLCE*^KI/KI^).

To generate the BAT-delivered mouse *Pcolce* gain-of-function model, pAAV-mouse *Pcolce* was directly injected into BAT of an HFD-fed male mice at the age of 8 and 11 weeks (for a total of 2 injections) with a concentration of 5 × 10^10^ genome copies/mice. The mock controls were injected with AAV-EGFR at the same concentration. Physiological studies were started 30 days after the second injection. Investigators were blinded to the mouse genotypes during experiments. An induction chamber filled with 2% isoflurane (Viatris) was used to induce anesthesia (Narcobit-E type II: Natume). The same concentration of the isoflurane was delivered through the nose cone, and mice were euthanized by exsanguination. The study was carried out in compliance with the ARRIVE guidelines and *Guide for the Care and Use of Laboratory Animals* (National Academies Press, 2011).

### Physiological analysis.

For the i.p. glucose tolerance test, mice were fasted for 4 hours with free access to water prior to the experiment. Glucose (D-(+)-glucose solution, Sigma-Aldrich, G8644) was i.p. administered at a dose of 1 g/kg body weight in the early afternoon, or was i.p. administered with insulin at 1:00 pm (Humulin R, Lilly Medical, 100 units/mL: 1 unit/kg body weight) without fasting. Tail vein blood samples were collected at 0, 15, 30, 60, and 120 minutes after injection, and blood glucose levels were measured immediately using a portable glucose analyzer (Sanwa Kagaku Kenkyusho Co., Ltd.).

Mice were fixed at the tablet under light anesthesia with isoflurane (1.5%–2% in oxygen). Echocardiography was performed using a Vevo 2100 or 3100 High-Resolution Imaging System (Fujifilm VisualSonics Inc.) equipped with a high-frequency transducer. Two-dimensional, M-mode, and tissue Doppler images were acquired in both parasternal long-axis and 4-chamber views. Standard cardiac parameters, including left ventricular end-diastolic and end-systolic dimensions, fractional shortening (FS), wall thickness, and E and e’ wave, were measured using the manufacturer’s software.

Exercise capacity was evaluated by treadmill running (Muromachi Kikai Co., Ltd.) according to the manufacturer’s protocol. After a 3-day adaptation period, mice were subjected to an incremental running test starting at a speed of 5 m/min with gradual increments (2 m/min every 2 min) until exhaustion, which was defined as the mouse remaining in the electrified resting zone (1.3 mA/s) for more than 5 seconds, at which point the treadmill was immediately stopped. Running distance, time to exhaustion, and maximum speed were recorded.

### Hemodynamic measurements and pressure-volume loop analysis.

Mice were anesthetized with 1.5%–2% isoflurane and placed on a temperature-controlled operating table to maintain normal body temperature. After a midline cervical incision, a tracheostomy was performed, and an ethylene tetrafluoroethylene cannula was inserted into the trachea. The animals were mechanically ventilated with room-temperature air using a small animal ventilator (PhysioSuite, Kent Scientific).

After thoracotomy, the heart was gently exteriorized. A small apical puncture was made with a 25-gauge needle, and a 1.2 F microtip pressure-volume catheter (Millar Inc.) was advanced into the left ventricular cavity through the puncture site. Left ventricular pressure and volume signals were continuously recorded using a Power Lab data acquisition system and Lab Chart software (ADInstruments). After stabilization, steady-state pressure-volume loops were obtained. To derive load-independent indices, transient occlusion of the inferior vena cava was performed, allowing calculation of the end-systolic pressure-volume relationship and the end-diastolic pressure-volume relationship. Data were analyzed using the pressure-volume loop module of Lab Chart Pro software (ADInstruments).

### CT scan analysis.

Mice were anesthetized with 1%–2% isoflurane in oxygen and subjected to in vivo CT scanning using a LaTheta LCT-200 system (Aloka). We performed CT scanning at 2 mm intervals from the diaphragm to the bottom of the abdominal cavity. Visceral and subcutaneous fat areas were quantified automatically using the manufacturer’s analysis software, and total fat mass was calculated as the sum of all slices.

### Cell culture and in vitro studies.

The mouse brown preadipocyte cell line was a gift from C. Ronald Kahn (Joslin Diabetes Center and Harvard Medical School, Section on Integrative Physiology and Metabolism, Boston, MA, USA) (43). The cell line was established from WT FVB mice, and was immortalized by infection with the retroviral vector pBabe encoding SV40T antigen. Cells were cultured in high-glucose DMEM (Gibco, 12430-062) with 10% FBS (Gibco, 10437-028) and 100 U/mL penicillin-streptomycin (Gibco, 5140122), and their differentiation was induced as described previously (44). Fully differentiated brown adipocytes were used for further analysis after 10 days of culture. The following drugs were used for fully differentiated brown adipocytes in in vitro experiments: T-5224 (10 μM: MedChemExpress, HY-12270) as an AP1 inhibitor. DOX (200 nM: Wako, 040-21521) was administered for 3 hours for immunofluorescence staining, 3-6 hours for Western blot, and 12 hours for qPCR analysis. H_2_O_2_ (10 μM: Nacalai Tesque, 20779-65) was administered for 1 hour for immunofluorescence staining, 6–12 hours for Western blot, and 12 hours for qPCR analysis. The AP1 inhibitor was administrated 1 hour before DOX or H_2_O_2_ treatment in in vitro studies. For immunofluorescence staining, brown adipocytes were cultured in 10 cm dishes and fully differentiated for 7 days. After differentiation, cells were harvested and counted, and then seeded onto glass-bottom dishes (Matsunami, D11130H) at a density of 1.5 × 10^5^ cells/mL. After 5–6 hours of incubation to allow cell attachment, cells were subjected to treatment with either DOX or H_2_O_2_. Subsequent fixation, permeabilization, and staining procedures were performed as described in *Histological analyses and immunostaining*.

### AAV.

The purified pAAV-mouse Pcolce or pAAV-EGFR (negative control) recombinant AAVDJ virus were made from pAAV[Exp]-CMV>mPcolce[NM_008788.2]:WPRE vector (VectorBuilder, AAVdjLP[VB211222-1285kyq]-C) or pAAV[Exp]-CMV> EGFR:WPRE vector (VectorBuilder, AAVdjLP[VB010000-9394npt]-C). The titer of purified AAV was quantified with QuickTiter AAV Quantitation kit (Cell Biolabs Inc, VPK-145).

### RNA analysis.

Total RNA was extracted from tissue and cultured cell samples using QIAzol Lysis reagent (QIAGEN, 79306) following the manufacturer’s protocol. Approximately 1 μg of RNA from each sample was used for downstream analysis. The concentration and purity of RNA were determined spectrophotometrically using a NanoDrop 2000 (Thermo Fisher Scientific). RNA samples were stored at −80°C until use. cDNA was synthesized from 1 μg of total RNA using the High-Capacity cDNA Reverse Transcription kit (Applied Biosystems, 205314), according to the manufacturer’s instructions, in a 20 μL reaction volume. qPCR was performed using a QuantStudio 6 Flex Real-Time PCR System (Applied Biosystems) with PowerUp SYBR Green Master Mix (Applied Biosystems, A25742). Primer sequences are listed below and were validated for amplification efficiency and specificity by melt curve analysis. The qPCR cycling conditions were uracil-DNA glycosylase activation at 50°C for 2 minutes, initial denaturation at 95°C for 2 minutes, followed by 40 cycles of denaturation at 95°C for 15 seconds and annealing/extension at 60°C for 1 minute. For absolute quantification, the copy number was calculated with Light Cycler 480 software version 1.5 (Roche) using the Fit Points method. Relative expression levels were normalized to the reference gene *Actb*, *Rplp0, 28s,* or *18s* and calculated using the 2^–ΔΔCt^ method. The primer information is listed in Supplemental Table 1.

### Public database studies.

Publicly available single-cell RNA-seq data from the Tabula Muris Senis (https://tabula-muris-senis.ds.czbiohub.org) consortium were analyzed using the CZ CELLxGENE Discover platform (https://cellxgene.cziscience.com/). BAT single-cell datasets from aged mice (24 months) were used for analysis.

### Western blot analysis.

Whole-cell or tissue lysates were prepared in lysis buffer (10 mM Tris-HCl, pH 8, 140 mM NaCl, 5 mM EDTA, 0.025% NaN3, 1% Triton X-100, 1% deoxycholate, 0.1% SDS, 1 mM PMSF, 5 μg mL^–1^ leupeptin, 2 μg mL^–1^ aprotinin, 50 mM NaF, and 1 mM Na_2_VO_3_). Then the lysates (40–50 μg) were resolved by SDS-PAGE. Proteins were transferred to a PVDF membrane (MilliporeSigma) that was incubated with the following primary antibodies: anti-PCPE-1 antibody (R&D Systems: MAB2239, lot UDN014091, clone 261845, Figure 4A; Proteintech: 28826-1-AP, lot 00137308, Figure 4J; Lifespan Biosciences LS-C74196, lot 210920, all other figures); anti-phospho-γH2AX (Ser139) antibody (Cell Signaling Technology, 9718, lot 21, clone 20E3); anti-phospho-c-Fos (Ser32) antibody (Cell Signaling Technology, 5348, lot 4, clone D82C12), anti-c-Fos antibody (Cell Signaling Technology, 2250, lot 12, clone 9F6), anti-phospho-c-Jun (Ser63) antibody (Cell Signaling Technology, 9261, lot 16), anti-c-Jun antibody (Cell Signaling Technology, 9165, lot11, clone 60A8), anti-alpha smooth muscle actin antibody (Abcam, ab21027, lot 1048914-1), anti-phospho-IRE1 (Ser724) antibody (Affinity Biosciences, AF7150, lot 32u2444), anti-IRE1 antibody (Abcam, ab37073, lot GR3354835-2), anti-pan-actin antibody (Cell Signaling Technology, 4968, lot 3), and anti-GAPDH antibody (Proteintech, 10494-1-AP, lot 00115763). All primary antibodies were used at a dilution of 1:1,000. Subsequently, incubation was performed with HRP-conjugated goat anti-rabbit IgG (H+L) (Jackson ImmunoResearch, 111-035-003), goat anti-rat IgG (H+L) (112-035-003), and rabbit anti-goat IgG (H+L) (305-035-003) at a dilution of 1:5,000. After incubation, proteins were detected by enhanced chemiluminescence (GE).

### ELISA.

The following kits were used for ELISA according to the instructions of the manufacturer: Mouse Procollagen C Proteinase Enhancer 1 ELISA kit (My BioSource, MBS021229), Human Procollagen C Proteinase Enhancer 1 ELISA kit (My BioSource, MBS722836S), and Mouse Collagen Type I ELISA kit (My BioSource, MBS724458).

### Histological analyses and immunostaining.

Samples of BAT and heart (LV) were harvested, fixed overnight in 10% formalin, embedded in paraffin, and sectioned for immunofluorescence, H&E (H&E Stain kit, ScyTek Laboratories, Inc., HAE-1), or Masson’s trichrome stain (Trichrome Stain kit, ScyTek Laboratories, Inc., TRM-1) (38), and photographed with a Biorevo (Keyence Co). Low magnification of the heart sections are demonstrated in Supplemental Figure 9 (for Figure 2F and 2K), Supplemental Figure 10 (for Figure 3C and 3H), and Supplemental Figure 11 (for Figure 4C, 4H, and 4L). For immunostaining, BAT deparaffinized sections were retrieved with citrate buffer, brown adipocytes were fixed with 4% paraformaldehyde (4% PFA, Thermo Fisher Scientific, J61899.AK), and incubated with anti-8-OHdG antibody (Bioss, bs-1278R, lot: BD1006918425) and anti-phospho-γH2AX (Ser139) antibody (Cell Signaling Technology, 9718, lot21, clone 20E3) at 1:100 dilution. Anti-rabbit Cy5 conjugate (Abcam, ab6564) at 1:200 dilution was used as a secondary antibody. The sections were stained with wheat germ agglutinin, Alexa Fluor 488 conjugate (Invitrogen, W11261, 1:10), and Hoechst (Life Technologies, 33258, 1:1,000) or DAPI (Invitrogen, D1306, 1:1,000), and photographed with a confocal microscope (C2, Nikon). Five (in vivo) or 3 (in vitro) fields were randomly selected from each section for quantification. For electron microscopy, heart tissue was fixed in 2.5% glutaraldehyde. Grids for electron microscopy were prepared by Masaaki Nameta at the core electron microscope facility of Niigata University, and electron microscopy studies were performed at Niigata University Medical Campus using a JEM1400 TEM. Fibrosis was detected with Masson’s trichrome stain, and 4 fields per section were randomly selected for quantification with the ImageJ (NIH) system. ROS were evaluated with dihydroethidium staining (WAKO, 041-28251, 10 μg/mL).

### Hydroxyproline assay.

Tissue hydroxyproline content was quantified using a modified colorimetric method based on chloramine T oxidation and Ehrlich’s reagent condensation. The heart tissue (20–30 mg) was homogenized in distilled water (100 μL per 10 mg tissue) and hydrolyzed with an equal volume of 10 N NaOH (Nacalai Tesque, 31511-05) at 120°C for 1 hour in sealed polypropylene vials. After cooling on ice, residual alkali was neutralized with 10 N HCl (Wako, 084-05425), and samples were centrifuged at 10,000*g* for 5 minutes. The supernatant was filtered through a 0.45 μm PVDF filter and kept on ice until use. Acetate citrate buffer was prepared from sodium acetate trihydrate (Wako, 198-01055), trisodium citrate (Nacalai Tesque, 31404-15), and citric acid (Wako, 035-03495) in a 40% v/v isopropanol/water solution. Chloramine-T (0.58% w/v sodium p-toluene sulfonamide trihydrate (Wako, 032-02182) in acetate citrate buffer) and Ehrlich’s reagents (15% w/v p-dimethylamino-benzaldehyde (Wako, 041-18045) in isopropanol) were freshly prepared before each assay. L-hydroxyproline (Wako, 080-01642) standards (0–2 μg/tube) were generated from a 10 μg/mL working solution. For each reaction, 20 μL of hydrolyzed sample or standard was mixed with 600 μL of 50% isopropanol and 100 μL of chloramine-T solution, incubated for 10 minutes at room temperature, followed by the addition of 500 μL Ehrlich’s reagent. The reaction mixture was heated at 50°C for 90 minutes, cooled to room temperature, and 150 μL of each reaction was transferred to a 96-well plate. Absorbance was measured at 558 nm using a microplate reader, and hydroxyproline content was calculated from the standard curve and normalized to tissue weight.

### Statistics.

All data are from different biological replicates. All statistical analyses were performed with SPSS software (version 30), and figures were generated with GraphPad Prism9. Outliers and abnormal values were recognized by box plot analyses with SPSS, and the details are described in the raw data files. All values, including outlier/abnormal values, were analyzed as mentioned. In studies with qPCR, ΔCP showing values with an outlier or abnormal value in the control group were excluded from further studies, and these are described in the Supporting Data Values file. SPSS uses Tukey’s method to identify outliers or abnormal values, and these are displayed on box plots based on the following algorithm: outlier (third quartile + 1.5 IQR, first quartile – 1.5 IQR), abnormal value (third quartile + 3 IQR, first quartile – 3 IQR). Data are shown as mean ± SEM. Differences between groups were examined by the independent-samples 2-tailed *t* test or 2-way ANOVA followed by Tukey’s multiple-comparison test or Dunnett’s T3 test, 1-way ANOVA, or Kruskal-Wallis test followed by Bonferroni’s multiple-comparison test for comparisons among more than 2 groups, unless otherwise specified. Levene’s test of equality of variances was used to test for homogeneity of variances and compared using a 2-tailed independent-samples *t* test. The 2-tailed Welch’s *t* test was used in further analyses when the assumption of homogeneity of variances was violated and when the groups differed in the number of samples studied. A *P* value of less than 0.05 was considered to indicate statistical significance in all analyses.

### Study approval.

The Scientific-Ethics Committees and IRB of National Cerebral and Cardiovascular Center, Niigata University, and Juntendo University approved the protocols of all human studies (protocols R23028, R23063, G2018-0023, 2015-2193, 2017-0102, E21-0075-H01, E22-0003-M01, H12-0871, P25-0015-H01), and each investigation was performed in accordance with the Declaration of Helsinki. All study participants provided written informed consent prior to participation.

All animal experiments were conducted in compliance with the animal protocol, which was reviewed by the IACUC of National Cerebral and Cardiovascular Center Research Institute, Juntendo University and Niigata University, and approved by the presidents of these universities.

### Data availability.

RNA-seq analysis was done as follows. Total RNA was isolated from BAT collected from young (12–13 wk) and aged (79 wk) C57BL/6NCrSlc mice with an RNeasy Mini kit (QIAGEN, 74104) and its quality was assessed by using the Agilent 2100 Bioanalyzer with the Agilent RNA6000 pico kit. The TruSeq Stranded mRNA LT Sample Prep kit (Illumina) was employed to construct 4 libraries according to the specifications of the manufacturer. Then, these libraries were analyzed on a NextSeq500 with a NextSeq 500/550 High Output kit v2 (Illumina). TopHat 2 (v2.0.13) was used for mapping reads to the reference genome (Ensembl GRCm38/mm10) with annotation data downloaded from the Ensembl Asia website (https://asia.ensembl.org/). The expression of each transcript was quantified as the number of fragments per kilobase of transcript per million fragments mapped (FPKM), and their expression was compared among 3 groups by Cuffdiff (included in Cufflinks version 2.2.1). Gene expression data obtained in these studies were deposited in NCBI’s GEO (GSE274901). The raw data supporting the findings of this study are provided in the Supporting Data Values file accompanying this article. We uploaded videos and images of cardiac echo in figshare (https://figshare.com/s/58099aac83e4681cf491).

## Author contributions

IS designed the studies and wrote the article. KW and JTP contributed to editing of the manuscript. YTH and IS performed the majority of experiments. YY, HT, JT, TMA, CHC, AJB, ZL, NSBO, TY, AI, JTP, KO, and TM aided in the in vivo studies. YL and SO performed bioinformatic studies. MA, SM, ST, and KW contributed to the generation of genetic mouse models. TI, HK, YS, DM, YM, TF, and HI contributed to the human study.

## Conflict of interest

The authors have declared that no conflict of interest exists.

## Funding support

- Fusion Oriented Research for disruptive Science and Technology (JST FOREST Program) (JPMJFR200L) (to IS).
- Japan Society for the Promotion of Science (JSPS) Grants-in-Aid for Scientific Researchers (B) (23K27602) (to IS).
- Japan Agency for Medical Research and Development (AMED) Project for Elucidating and Controlling Mechanisms of Aging and Longevity under grants JP21gm5010002, JSPS KAKENHI (to IS).
- Grants-in-Aid for Encouragement of Young Scientists (A) (16H06244) (to IS).
- Intramural Research Fund for Cardiovascular Diseases of the National Cerebral and Cardiovascular Center, Daiichi Sankyo research grant (TaNeDS) (to IS).
- Japan Health Research Promotion Bureau (to IS).
- Grant-in-Aid for Scientific Research (C) (22K08215) (to YY).
- Daiichi Sankyo Foundation of Life Science (to YY).
- Senshin Medical Research Foundation (to YY).
- Astellas Foundation for Research on Metabolic Disorders (to YY).
- Japan Diabetes Foundation, Life Science Foundation of Japan (to YY).
- Grant from Bourbon Corporation (to TM and YY).
- Foundation for Medical & Pharmaceutical Research (to IS).
- Uehara Memorial Foundation (to IS).
- Kowa Life Science Foundation (to IS).
- Manpei Suzuki Diabetes Foundation (to IS).
- MSD Life Science Foundation (to IS).
- Public Interest Incorporated Foundation (to IS).
- Kanae Foundation research grant (to IS).
- Inamori Foundation (to IS).
- Terumo Foundation for Life Sciences and Arts (to IS).
- Ono Medical Research Foundation (to IS).
- Nakajima Foundation (to IS).
- Suzuken Memorial Foundation. (to IS).
- Hokuto Corporation (to IS).
- Mochida Memorial Foundation for Medical & Pharmaceutical Research (to IS).
- Cell Science Research Foundation (to IS).
- Chugai Foundation for Innovative Drug Discovery Science (Tokyo Biochemical Research Foundation) (to IS).
- Research grant from Naito Foundation (to IS).
- Japan Geriatrics Society (to IS).
- Japan Diabetes Foundation and Novo Nordisk Pharma Ltd (to IS).
- Nakatomi Foundation (to IS).
- Kobayashi Magobei Memorial Medical Promotion Foundation of Japan (to IS).
- President’s Grant for Interfaculty Collaboration, Juntendo University (to IS).
- Japanese Circulation Society Grant for Future-Pioneering Doctors for Basic Research (to IS).
- Food Science Institute Foundation (Ryoushoku Kenkyukai) (to IS).
- Japan Society for the Promotion of Science (JSPS) Bilateral Joint Research Program with the National Research Foundation of Korea (NRF) (to IS).
- The Tojuro Iijima Foundation for Food Science and Technology (to IS).
- JSPS Grant in Aid for Early-Career Scientists (26K19267 and 24K18656) (to YTH).
- JSPS Grant-in-Aid for Research Activity Start-up (23K19579) (to YTH).
- Japan Foundation for Applied Enzymology (to YTH).
- Mochida Memorial Foundation for Medical and Pharmaceutical Research (to YTH).
- Suzuken Memorial Foundation (to YTH).
- MSD Life Science Foundation (to YTH).
- Research and Development Grants for Cardiovascular Diseases (Young Investigator Program) from the National Cerebral and Cardiovascular Center (to YTH).

## Supplementary Material

Supplemental data

Unedited blot and gel images

Supplemental table 1

Supporting data values

## Figures and Tables

**Table 1:**
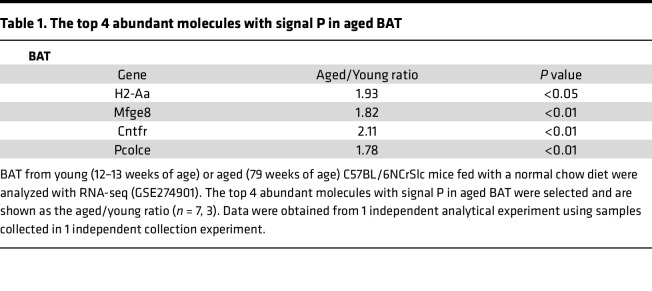
The top 4 abundant molecules with signal P in aged BAT

## Version 1 04/09/2026

In-Press Preview

## Version 2 07/08/2026

Electronic publication

## References

[1] Savarese G (2022). Global burden of heart failure: a comprehensive and updated review of epidemiology. Cardiovasc Res.

[2] Roberts NLS (2019). Global, regional, and national incidence, prevalence, and years lived with disability for 354 diseases and injuries for 195 countries and territories, 1990-2017: a systematic analysis for the global burden of disease study. Lancet.

[3] Shah KS (2017). Heart failure with preserved, borderline, and reduced ejection fraction: 5-year outcomes. J Am Coll Cardiol.

[4] Zinman B (2015). Empagliflozin, cardiovascular outcomes, and mortality in type 2 diabetes. N Engl J Med.

[5] McMurray JJV (2019). Dapagliflozin in patients with heart failure and reduced ejection fraction. N Engl J Med.

[6] McMurray JJV (2014). Angiotensin-neprilysin inhibition versus enalapril in heart failure. N Engl J Med.

[7] Armstrong PW (2020). Vericiguat in patients with heart failure and reduced ejection fraction. N Engl J Med.

[8] Swedberg K (2010). Ivabradine and outcomes in chronic heart failure (SHIFT): a randomised placebo-controlled study. Lancet.

[9] Solomon SD (2022). Dapagliflozin in heart failure with mildly reduced or preserved ejection fraction. N Engl J Med.

[10] Anker SD (2021). Empagliflozin in heart failure with a preserved ejection fraction. N Engl J Med.

[11] Packer M (2025). Tirzepatide for heart failure with preserved ejection fraction and obesity. N Engl J Med.

[12] Butler J (2024). Semaglutide versus placebo in people with obesity-related heart failure with preserved ejection fraction: a pooled analysis of the STEP-HFpEF and STEP-HFpEF DM randomised trials. Lancet.

[13] Kosiborod MN (2024). Semaglutide versus placebo in patients with heart failure and mildly reduced or preserved ejection fraction: a pooled analysis of the SELECT, FLOW, STEP-HFpEF, and STEP-HFpEF DM randomised trials. Lancet.

[14] Ibe T, Borlaug BA (2025). Glucagon-like peptide 1 receptor agonists and cardiovascular disease. Annu Rev Med.

[15] Borlaug BA (2023). Heart failure with preserved ejection fraction: JACC scientific statement. J Am Coll Cardiol.

[16] Redfield MM, Borlaug BA (2023). Heart failure with preserved ejection fraction: a review. JAMA.

[17] Lewis GA (2017). Biological phenotypes of heart failure with preserved ejection fraction. J Am Coll Cardiol.

[18] Mann DL (2004). Targeted anticytokine therapy in patients with chronic heart failure: results of the randomized etanercept worldwide evaluation (RENEWAL). Circulation.

[19] Ridker PM (2017). Antiinflammatory therapy with canakinumab for atherosclerotic disease. N Engl J Med.

[20] Hsiao YT (2024). PCPE-1, a brown adipose tissue-derived cytokine, promotes obesity-induced liver fibrosis. EMBO J.

[21] Cataldo LR (2022). The human batokine EPDR1 regulates β-cell metabolism and function. Mol Metab.

[22] Lagoutte P (2021). Procollagen C-proteinase enhancer-1 (PCPE-1), a potential biomarker and therapeutic target for fibrosis. Matrix Biol Plus.

[23] Ouchi N (2011). Adipokines in inflammation and metabolic disease. Nat Rev Immunol.

[24] Rosen ED, Spiegelman BM (2014). What we talk about when we talk about fat. Cell.

[25] Shimizu I (2013). Semaphorin3E-induced inflammation contributes to insulin resistance in dietary obesity. Cell Metab.

[26] Hotamisligil GS (1993). Adipose expression of tumor necrosis factor-alpha: direct role in obesity-linked insulin resistance. Science.

[27] Chertow GM (2024). IL-6 inhibition with clazakizumab in patients receiving maintenance dialysis: a randomized phase 2b trial. Nat Med.

[28] Everett BM (2019). Anti-inflammatory therapy with canakinumab for the prevention of hospitalization for heart failure. Circulation.

[29] Ouellet V (2012). Brown adipose tissue oxidative metabolism contributes to energy expenditure during acute cold exposure in humans. J Clin Invest.

[30] Branca RT (2018). Accurate quantification of brown adipose tissue mass by xenon-enhanced computed tomography. Proc Natl Acad Sci U S A.

[31] Leitner BP (2017). Mapping of human brown adipose tissue in lean and obese young men. Proc Natl Acad Sci U S A.

[32] Cypess AM (2009). Identification and importance of brown adipose tissue in adult humans. N Engl J Med.

[33] Van Ham WB (2022). Clinical phenotypes of heart failure with preserved ejection fraction to select preclinical animal models. JACC Basic Transl Sci.

[34] Hahn VS (2020). Endomyocardial biopsy characterization of heart failure with preserved ejection fraction and prevalence of cardiac amyloidosis. JACC Heart Fail.

[35] Toischer K (2017). Cardiomyocyte proliferation prevents failure in pressure overload but not volume overload. J Clin Invest.

[36] Yoshida Y (2022). Brown adipose tissue dysfunction promotes heart failure via a trimethylamine N-oxide-dependent mechanism. Sci Rep.

[37] Leach JP (2017). Hippo pathway deficiency reverses systolic heart failure after infarction. Nature.

[38] Nguyen NUN (2020). A calcineurin-Hoxb13 axis regulates growth mode of mammalian cardiomyocytes. Nature.

[39] Bauersachs J (2021). Heart failure drug treatment: the fantastic four. Eur Heart J.

[40] Mishina M, Sakimura K (2007). Conditional gene targeting on the pure C57BL/6 genetic background. Neurosci Res.

[41] Yuza K (2018). Different roles of sphingosine kinase 1 and 2 in pancreatic cancer progression. J Surg Res.

[42] Mizuno-Iijima S (2021). Efficient production of large deletion and gene fragment knock-in mice mediated by genome editing with Cas9-mouse Cdt1 in mouse zygotes. Methods.

[43] Klein J (1999). beta(3)-adrenergic stimulation differentially inhibits insulin signaling and decreases insulin-induced glucose uptake in brown adipocytes. J Biol Chem.

[44] Fasshauer M (2001). Essential role of insulin receptor substrate 1 in differentiation of brown adipocytes. Mol Cell Biol.

