# Supplementary material for: PCPE-1 promotes cardiac fibrosis with aging and obesity
Source: JCI Insight. 2026 Apr 9;11(13):e195508. doi: 10.1172/jci.insight.195508 (PMC13460844; doi:10.1172/jci.insight.195508)
Supplement: Supplementary file 1 [file jciinsight-11-195508-s039.pdf]

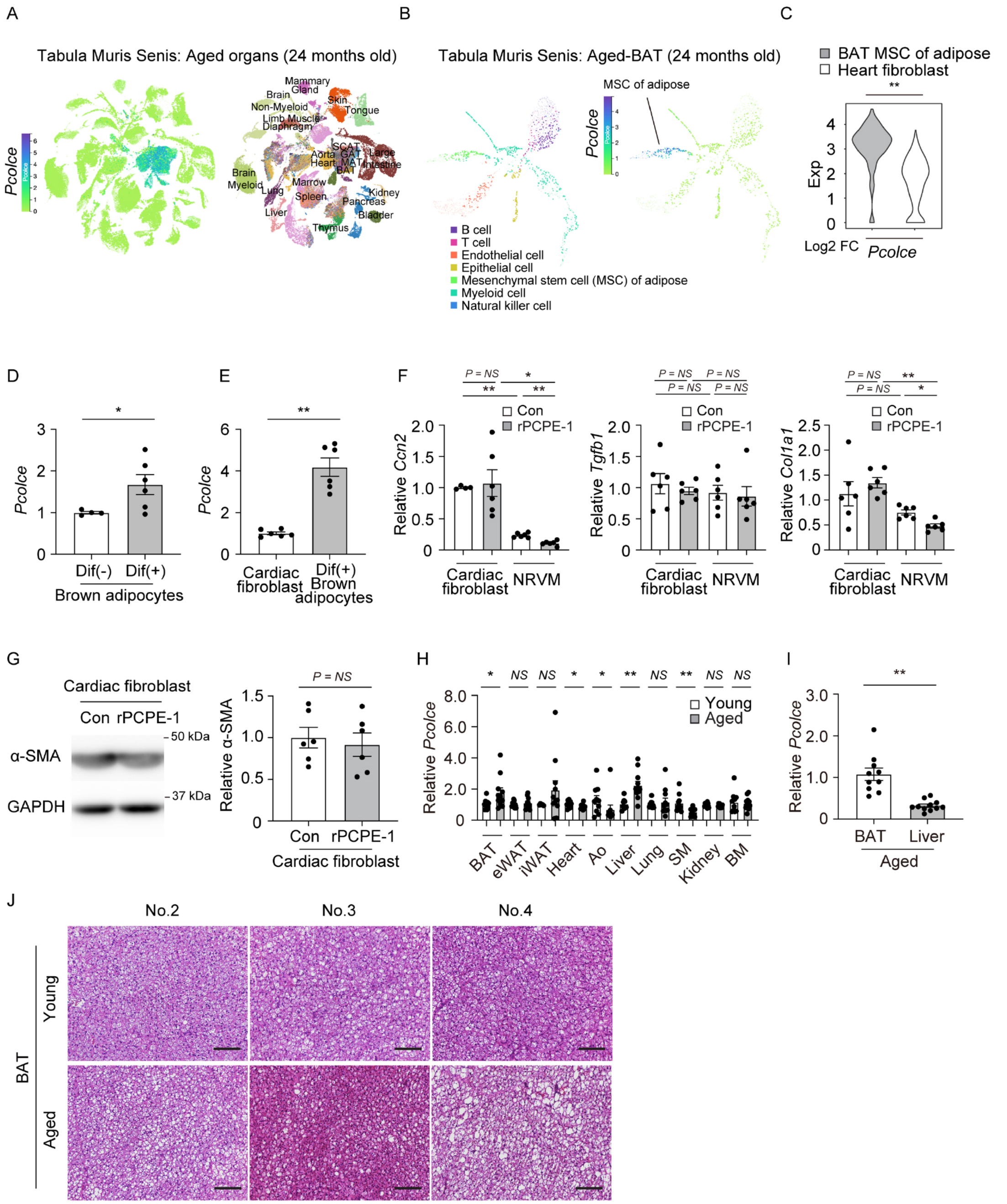

Supplemental Figure 1.

(A, B, C) Tabula Muris Senis testing transcript *Pcolce* in systemic organs (A), BAT (B) or indicated cells (C) in mice aged 24 months. For studies in Figure 1, D-G, brown adipocytes, cardiac fibroblasts, and ventricular myocytes (NRVM) were collected from

neonatal rats. **(D, E)** qPCR testing the level of transcript *Pcolce* in brown adipocytes subjected to differentiation (Dif(+)) or not (Dif(-)) (*n* = 6, 6)(D), Dif(+) brown adipocytes and cardiac fibroblasts (*n* = 6, 6)(E). **(F)** qPCR study in cardiac fibroblasts and NRVM administered with recombinant PCPE-1 protein (rPCPE-1) (*n* = 6, 6, 6, 6) (F) (Housekeeping genes: *28s* (D) and *Rplp0* (E, F)). **(G)** Western blot analysis of α-SMA in cardiac fibroblasts administered with or without rPCPE-1. Right panel indicates quantification. **(H, I)** qPCR study testing the level of transcript *Pcolce* in systemic organs (H)(*n* = 10, 11) or BAT and liver (I)(*n* = 11, 11) in young (12-week-old) and aged (109- to 125-week-old) mice (Housekeeping genes: *Rplp0* (H), and *Actb* (I)). **(J)** Hematoxylin-eosin (HE) staining of BAT from the indicated mice at 12 or 116 weeks of age. No. 1 mice are demonstrated in Figure 1C. Scale bar = 100 μm. Supplemental Figure 1F: two-way ANOVA followed by Dunnett T3 (*Ccn2* and *Col1a1*) or Tukey's multiple comparison (*Tgfb1*); others: independent-samples T-test. Data information: Data were analyzed from one public database (Tabula Muris Senis) (A-C). Representative hematoxylin and eosin-stained images from one series of observations (J), and other data were obtained from one representative analytical experiment out of at least two independent experiments showing similar results. Data in panels F-I were obtained from one independent experiment. $^*P < 0.05$, $^{**}P < 0.01$. Values are presented as mean ± SEM. NS = not significant.

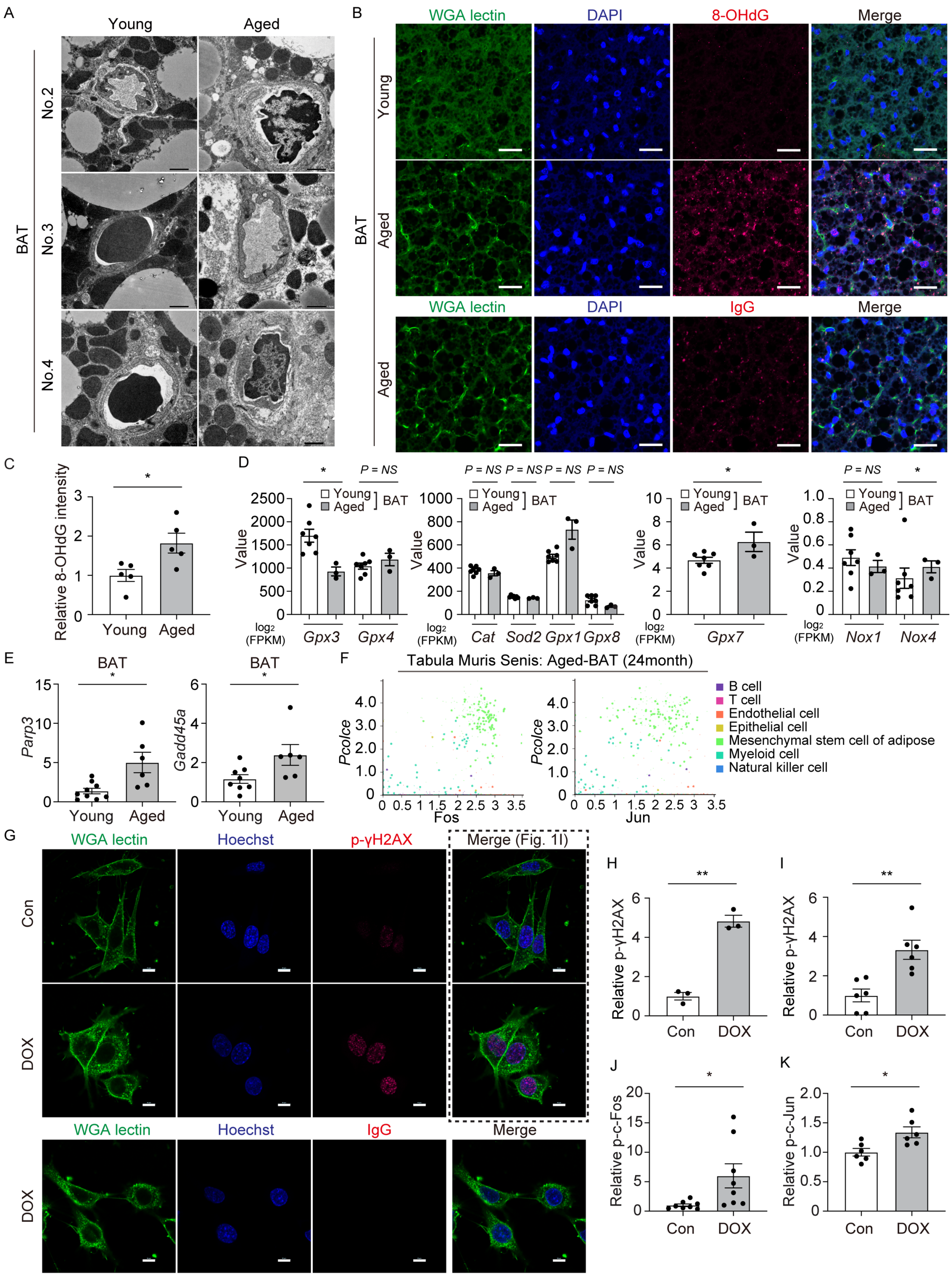

Supplemental Figure 2.

(A) Electron microscopic findings on the BAT from mice aged 12 or 112-121 weeks of

age. Scale bar = 2 μm. No. 1 mice are demonstrated in Figure 1D. **(B, C)** Immunofluorescent staining for 8-OHdG, WGA lectin, and DAPI in the aged BAT (young : 11-12 w of age, aged: 116 w of age)(B) and its quantification (C)(*n* = 5, 5). **(D)** Results of RNAseq dataset analyzing BAT collected from young (12-13 w of age) and aged (79 w of age) C57BL/6NCrSlc mice (GSE274901) (*n* = 7, 3). **(E)** Results from qPCR showing transcript *Parp3* and Gadd45a in the BAT of the indicated mice (*n* = 9, 6) **(**Housekeeping gene: *28s*). **(F)** Tabula Muris Senis testing transcript *Pcolce* in BAT together with *Fos* or *Jun* in mice aged 24 months. **(G)** Immunofluorescent staining for phospho-γH2AX, WGA lectin, and Hoechst in differentiated brown adipocytes administered with doxorubicin (DOX). For the negative control study, IgG was administered instead of the primary antibody targeting phospho-γH2AX. Indicated merged panels were also shown in the Figure. 1I. Scale bar = 10 μm. **(H)** Quantification data for phospho-γH2AX positive nucleus related to Figure 1I (*n* = 3, 3). **(I-K)** Quantification data for γH2AX (H)(*n* = 6, 6), phospho c-Fos (I) (*n* = 8, 8), or phospho c-Fos (J) (*n* = 6, 6) relative to GAPDH, related to Figure 1, J-L. All data were analyzed by an independent-samples T-test. Data information: Representative EM images from one series of observations (A). Analyses from one public database (Tabula Muris Senis) (F), and other data were obtained from one representative analytical experiment out of at least two independent experiments showing similar results. **P* < 0.05, ***P* < 0.01. Values are presented as mean ± SEM. NS = not significant.

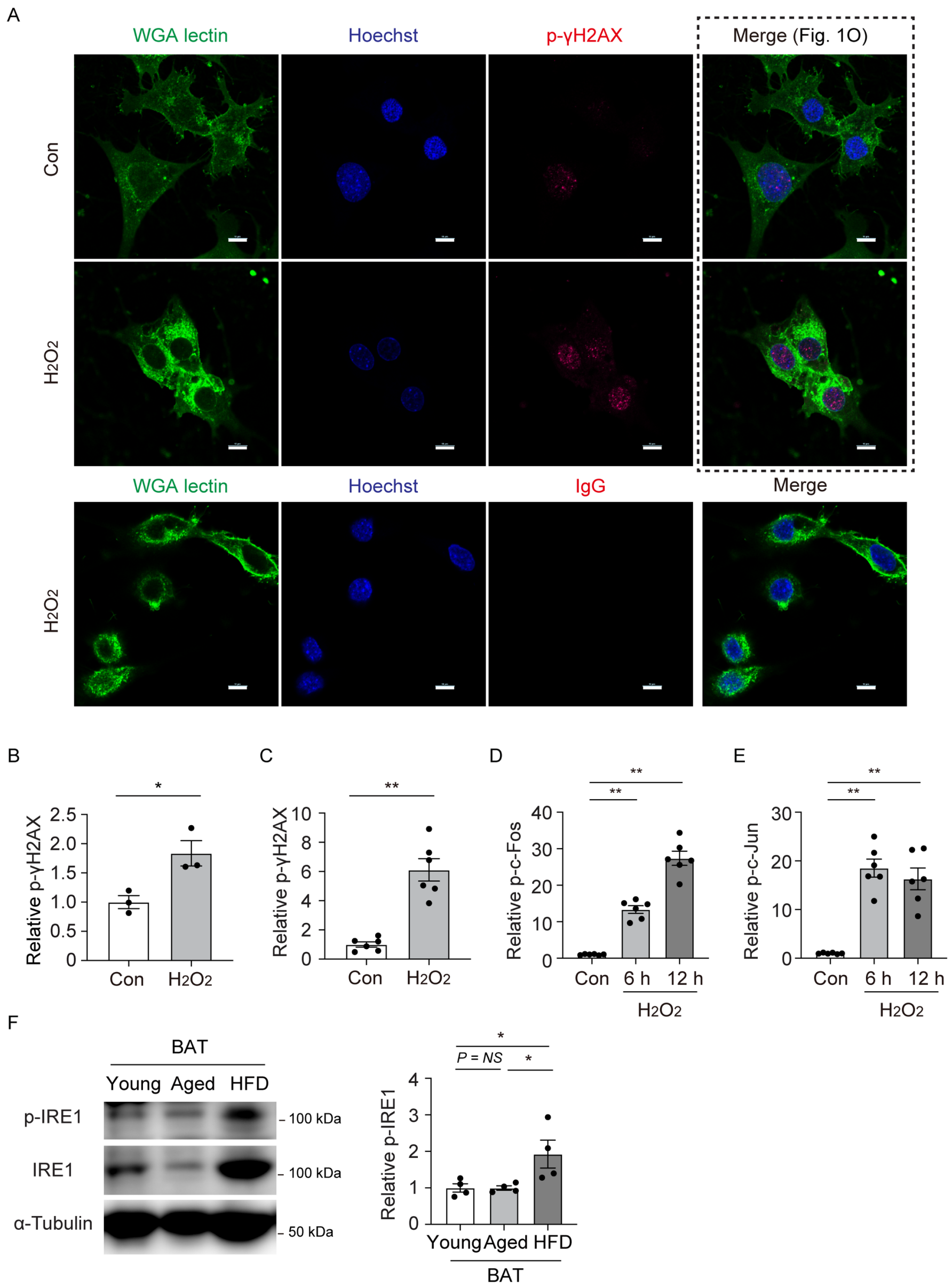

Supplemental Figure 3.

(A) Immunofluorescent staining for phospho-γH2AX, WGA lectin, and Hoechst in differentiated brown adipocytes administered with H_2O_2. For the negative control study, IgG was administered instead of the primary antibody targeting phospho-γH2AX.

Indicated merged panels were also shown in the Figure 1O. Scale bar = 10 μm. **(B)** Quantification data for phospho-γH2AX positive nucleus related to Figure 1O (*n* = 3, 3). **(C-E)** Quantification data for γH2AX (C) (*n* =6, 6), phospho c-Fos (D) (*n* = 6, 6, 6), or phospho c-Fos (E) (*n* = 6, 6, 6) relative to GAPDH, related to Figure 1, P-R. (**F**) Western blot analysis of phospho-IRE1, IRE1 and α-Tubulin in the BAT of the C57BL/6NCrSlc mice aged 11-12 weeks (Young) or 110-135 weeks (Aged) fed normal chow, and 45-week-old C57BL/6NCrSlc mice fed a high-fat diet (HFD) (*n* = 4, 4, 4). All data were analyzed by an independent-samples T-test, except for Supplemental Figure 3, D-F analyzed with one-way ANOVA (D, E) or Kruskal-Wallis test (F) followed by Bonferroni multiple comparison test. Data information: Data were obtained from one representative analytical experiment out of at least two independent experiments showing similar results. $^{*}P < 0.05$, $^{**}P < 0.01$. Values are presented as mean ± SEM. NS = not significant.

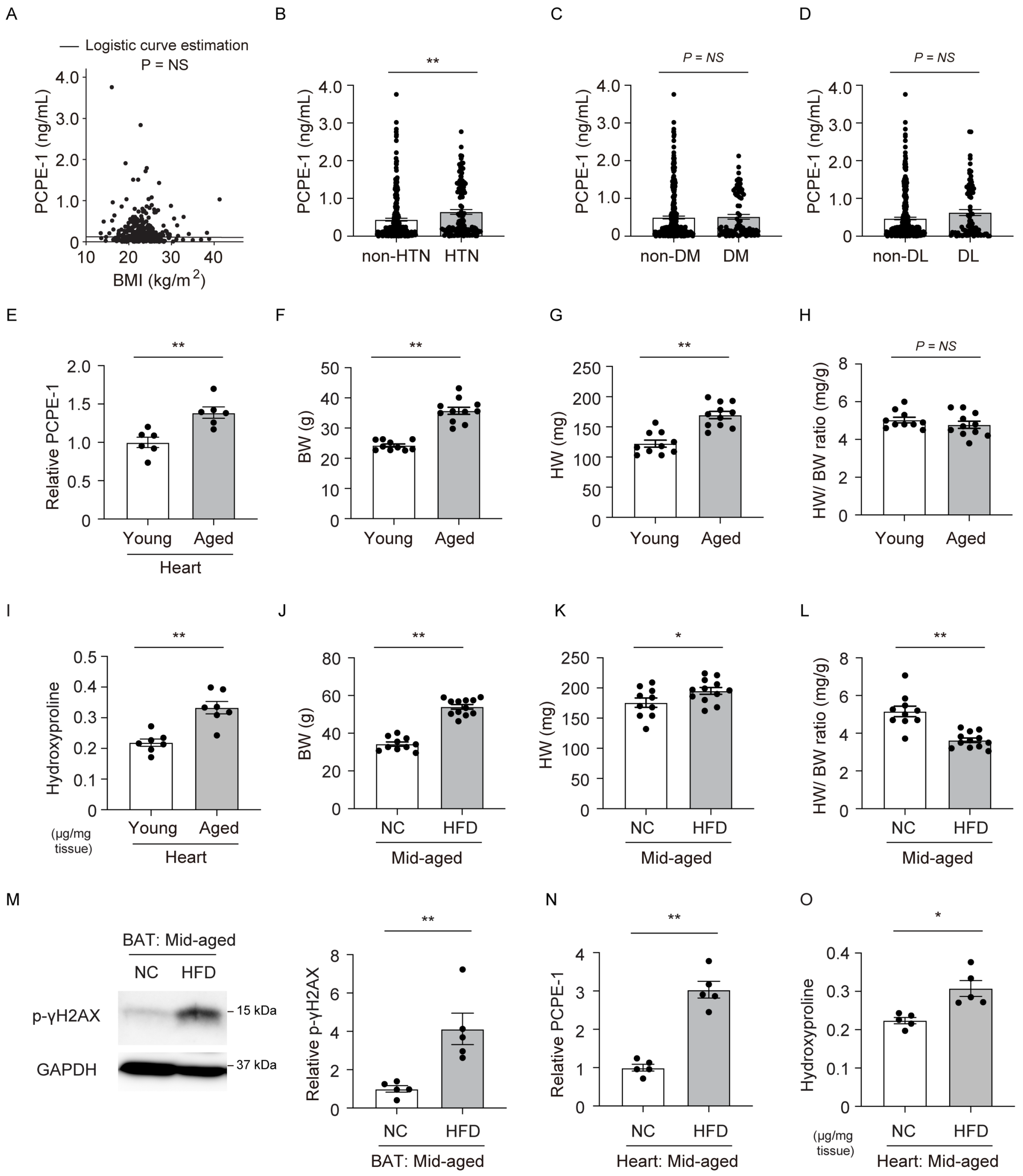

Supplemental Figure 4.

(A) BMI (data available for 325 of 405 participants) and plasma PCPE-1 (ELISA) were analyzed for correlation. **(B, C, D)** Level of plasma PCPE-1 in individuals with hypertension (HTN) (*n* = 123), non-HTN (*n* = 282)), type 2 diabetes (DM) (*n* = 83), non-DM (*n* = 322), dyslipidemia (DL) (*n* = 80), and non-DL (*n* = 325). **(E)** Quantification data

for cardiac PCPE-1 relative to GAPDH in mice related to Figure 2D (*n* = 6, 6). **(F-I)** Body weight (BW) (F) (*n* = 10, 11), heart weight (HW) (G) (*n* = 10, 11), HW/BW ratio (H) (*n* = 10, 11), Hydroxyproline assay of hearts (I) (*n* = 7, 7) in mice related to Figure 2. **(J-L)** Body weight (BW) (J) (*n* = 10, 12), heart weight (HW) (K) (*n* =10, 12), and HW/BW ratio (L) (*n* = 10, 12) of the mice related to Figure 2. (**M**) Western blot analysis of phospho-γH2AX in the BAT of the C57BL/6NCrSlc mice aged 44-45 weeks fed normal chow (NC) or a high-fat diet (HFD). The right panels indicate the quantification relative to GAPDH (*n* = 5, 5). **(N)** Quantification data for cardiac PCPE-1 relative to GAPDH of mice related to Figure 2I (*n* = 5, 5). (**O**) Hydroxyproline assay of hearts (*n* = 5, 5). Supplemental Figure 4A: logistic curve estimation; others: independent-samples T-test.

Data information: Samples obtained from two biobanks were analyzed in two independent experiments (A-D). Other data were obtained from one representative analytical experiment out of at least two independent experiments showing similar results. Data in panels I and O were obtained from one independent experiment. $*P < 0.05$, $**P < 0.01$. Values are presented as mean ± SEM. NS = not significant.

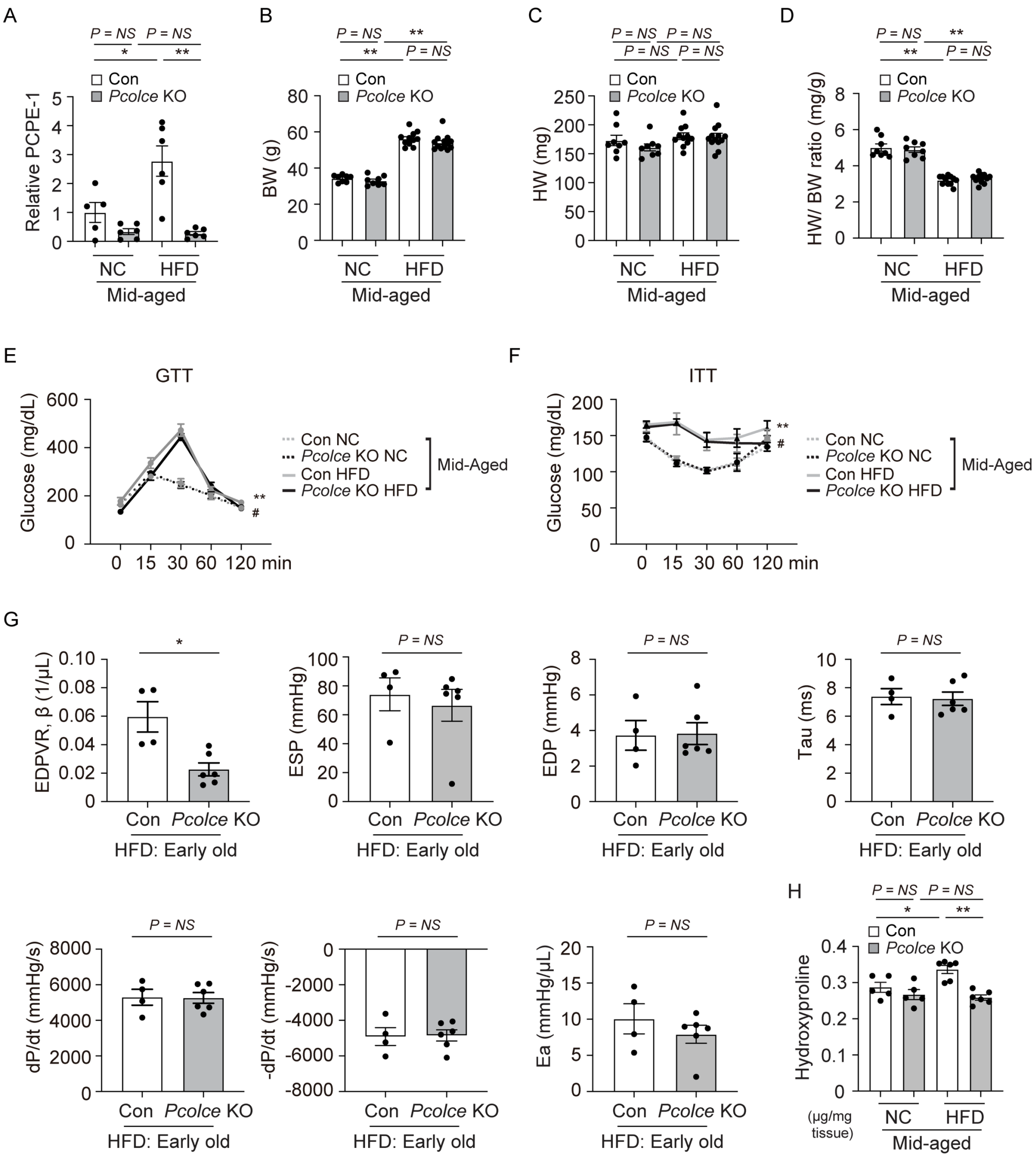

Supplemental Figure 5.

A normal chow (NC) or a high fat diet (HFD) fed systemic *Pcolce* KO male mice were studied at 41-46 weeks of age (mid-aged), otherwise mentioned. **(A)** Quantification data for cardiac PCPE-1 relative to GAPDH in the indicated mice related to Figure 3A (n =6, 6, 6, 6). **(B-D)** Body weight (BW) (B) (n = 8, 8,11,13), heart weight (HW) (C) (n = 8, 8,11,13), and HW/BW ratio (D) (n = 8, 8,11,13) in mice related with fig. 3. **(E, F)** Glucose

tolerance test (GTT) (E) (*n* = 9, 8, 4, 6) or insulin tolerance test (ITT) (F) (*n* = 6,6,6,8)(** indicates Con NC vs Con HFD, # indicates Pcolce KO NC vs Pcolce KO HFD) (L) in mice aged 60-63w (Early old). (**G**) Pressure-Volume loop analyses showing end-diastolic pressure-volume relationship (EDPVR), End-Systolic Pressure (ESP), End-Diastolic Pressure (EDP), Time constant of isovolumic relaxation (Tau), First derivative of pressure over time (dP/dt), Negative first derivative of pressure over time (-dP/dt), and effective Arterial Elastance (Ea) in the left ventricle of mice aged 61-64w (Early old)(*n* =4, 6). (**H**) Hydroxyproline assay of hearts (*n* = 5, 5, 6, 6). Supplemental Figure 5, B-D: two-way ANOVA followed by Tukey's (B, C, H) or Dunnett's T3 multiple comparison test (D); Supplemental Figure 5, E and F: 2-way repeated measures followed by Tukey's multiple comparison test; others: independent-samples T-test. Data information: Data were obtained from one representative analytical experiment out of at least two independent experiments showing similar results. Data in panel H were obtained from one independent experiment. * and # $P < 0.05$, **$P < 0.01$. Values are presented as mean ± SEM. NS = not significant.

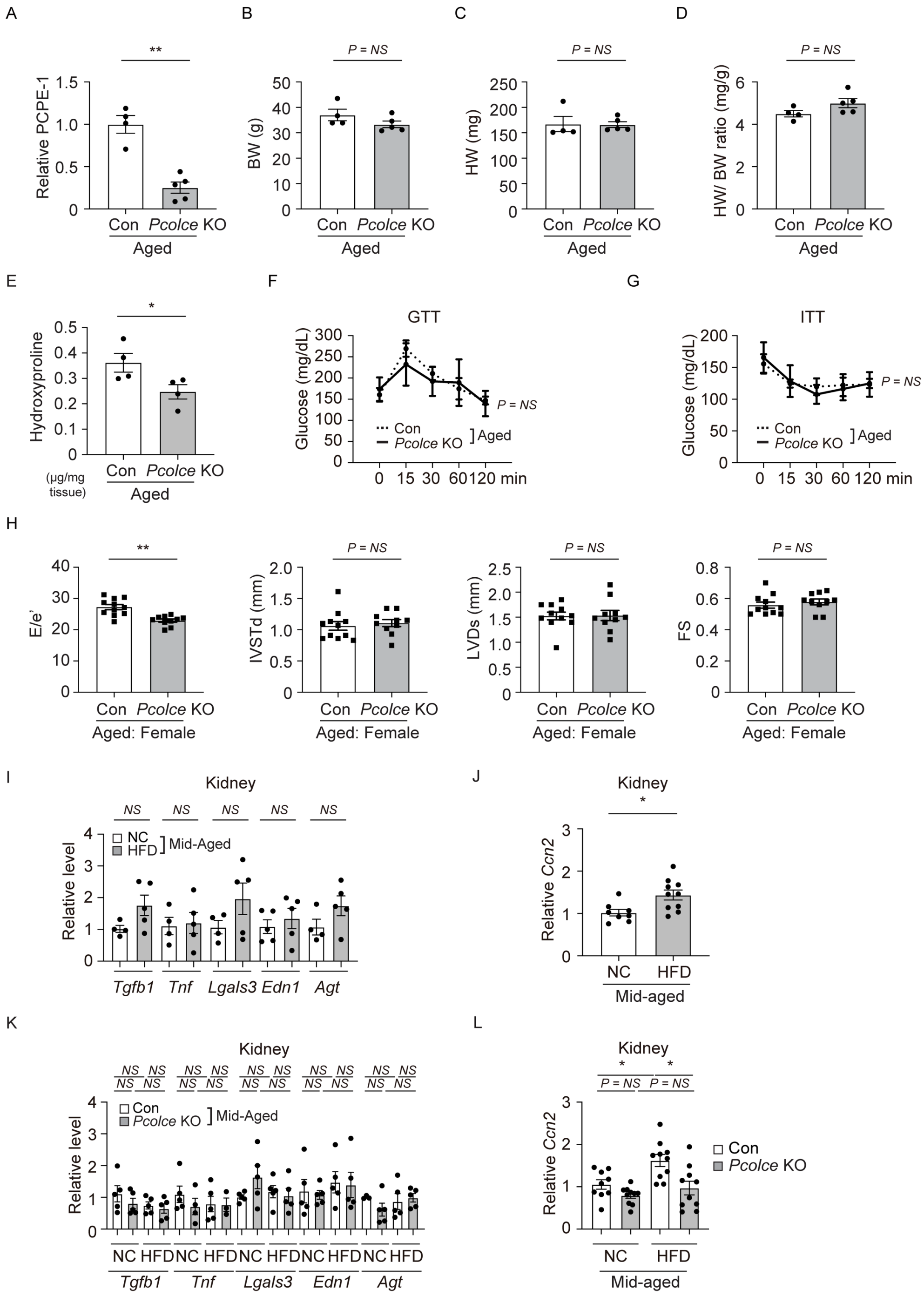

Supplemental Figure 6.

A normal chow (NC) fed systemic Pcolce KO male mice aged 78-80 weeks (A-G), and

44-45 weeks-of-age (Mid-aged) C57BL/6NCrSlc mice (H, I) and systemic Pcolce KO male mice (J, K) fed with a NC or a high-fat diet (HFD) were studied. (**A**) Quantification data for cardiac PCPE-1 relative to GAPDH in mice related to Figure 3F (n = 4, 5). (**B, C, D**) Body weight (BW) (B) (n = 4, 5), heart weight (HW) (C) (n = 4, 5), and HW/BW ratio (D) (n = 4, 5) in mice related to Figure 3. (**E**) Hydroxyproline colorimetric assay testing hearts of the indicated mice (n = 4, 5). (**F, G**) Glucose tolerance test (GTT) (F) (n = 4, 5) or insulin tolerance test (ITT) (G) (n = 4, 5). (**H**) Echocardiographic findings in female mice aged 92weeks: E/e' (marker for diastolic dysfunction), IVST (interventricular septum thickness), LVDs (left ventricular systolic dimension), FS (fractional shortening) (n = 11, 10). Data points (squares) indicate females. (**I-L**) qPCR results for transcripts *Tgfb1*, *Tnf*, *Lgals3*, *Edn1*, *Agt* (I, K) and *Ccn2* (J, L) in kidneys (I and K) (n = 5), (J and L) (n = 10) (Housekeeping gene: Rplp0). Supplemental Figure 6, E and F: 2-way repeated measures ANOVA; supplemental Figure 6K: two-way ANOVA followed by Tukey's comparison test; Supplemental Figure 6L: Kruskal-Wallis test followed by the Bonferroni multiple comparison test; others: independent-samples T-test. Data information: Data were obtained from one representative analytical experiment out of at least two independent experiments showing similar results. Data in panel E were obtained from one independent experiment. *P < 0.05, **P < 0.01. Values are presented as mean ± SEM. NS = not significant.

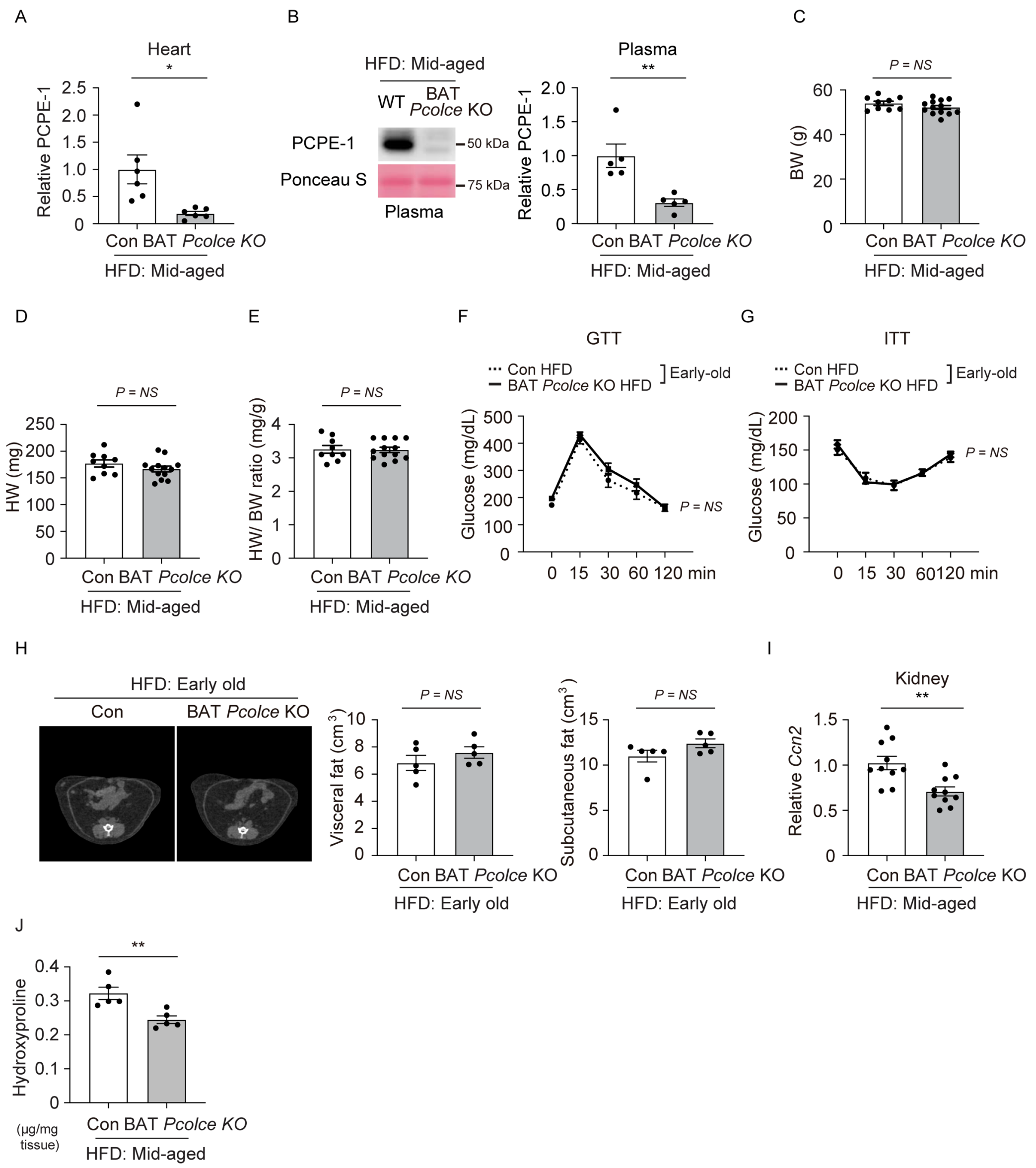

Supplemental Figure 7.

Littermate control (Con) or BAT-specific *Pcolce* knockout mice (BAT *Pcolce* KO) were fed with a high-fat diet (HFD) from 4 weeks of age and studied during 41-46 weeks of age (Mid-aged), otherwise mentioned. **(A)** Quantification data for cardiac PCPE-1 relative to GAPDH in the indicated mice related to Figure 4A (n = 6, 6). **(B)** Western blot analysis of plasma PCPE-1 expression in the indicated mice. The right panel indicates the

quantification (*n* = 5, 5). **(C-E)** Body weight (BW) (C) (*n* = 9, 13), heart weight (HW) (D) (*n* = 9, 13), HW/BW ratio (E) (*n* = 9, 13) in the indicated mice related to Figure 4. **(F, G)** Glucose tolerance test (GTT) (F) (*n* = 6, 7) or insulin tolerance test (ITT) (G) (*n* = 6, 7) in the indicated mice aged 67-67 weeks of age (Early old). **(H)** CT analysis of the HFD fed indicated mice aged 61-62 weeks (Early old). The graph shows the volume of visceral fat or subcutaneous fat tissue weight estimated by CT (*n* = 5, 5). **(I)** Results from qPCR showing transcript *Ccn2* in the indicated mice aged 44-45 weeks (Mid-aged)(*n* = 10, 10). *Rplp0* was used as a housekeeping gene. **(J)** Hydroxyproline colorimetric assay testing hearts of the indicated mice (*n* = 5, 5). Supplemental Figure 7, F and G: 2-way repeated measures ANOVA; others: independent-samples T-test. Data information: Representative CT scan images from one series of observations (H), and other data were obtained from one representative analytical experiment out of at least two independent experiments showing similar results. Data in panel J were obtained from one independent experiment. $^*P < 0.05$, $^{**}P < 0.01$. Values are presented as mean ± SEM. NS = not significant.

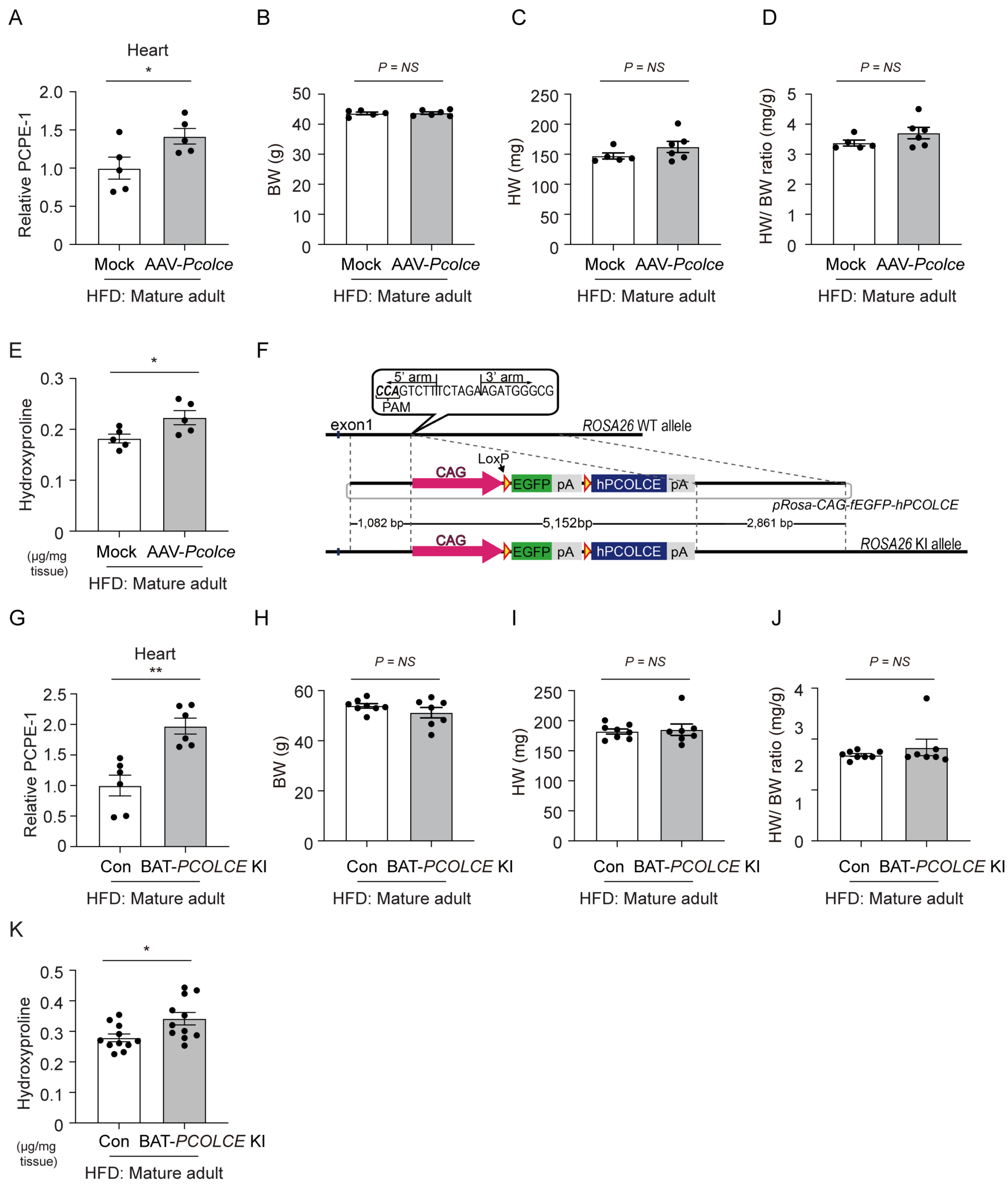

Supplemental Figure 8.

Adeno-associated virus (AAV) encoding *Pcolce* was injected in the BAT of C57BL/6NCrSlc mice fed with an HFD and studied at 15 weeks of age (Mature-adult). **(A)** Quantification data for cardiac PCPE-1 relative to GAPDH in the indicated mice

related to Figure 4F (n = 5, 5). **(B, C, D)** Body weight (BW) (B) (n = 5, 6), heart weight (HW) (C) (n = 5, 6), HW/BW ratio (D) (n = 5, 6) (M) in the indicated mice related with Figure 4. **(E)** Hydroxyproline colorimetric assay testing hearts of the indicated mice (n = 5, 5). **(F)** Generation of BAT-specific human *PCOLCE* knock-in (KI) mice. The knock-in mice were crossed with UCP1-$Cre^{+/-}$ mice to generate BAT-specific *PCOLCE* knock-in mice (UCP1-$Cre^{+/-}$; *PCOLCE* $^{KI/KI}$) (BAT-*PCOLCE* KI). BAT-*PCOLCE* KI mice were also fed with an HFD from 4w of age and studied at 25 weeks of age (Mature adult). **(G)** Quantification data for cardiac PCPE-1 relative to GAPDH in the indicated mice related to Figure 4J (n = 6, 6). (G, H, I) Body weight (BW) **(H)** (n = 8, 7), heart weight (HW) **(I)** (n = 8, 7), HW/BW ratio **(J)** (n = 8, 6) in the indicated mice related to Figure 4. **(K)** Hydroxyproline colorimetric assay testing hearts of the indicated mice (n = 11, 11). All data were analyzed by an independent-samples T-test. Data information: Data in panel A-E, and K were obtained from one independent experiment, and other data were obtained from one representative analytical experiment out of at least two independent experiments showing similar results. *$P < 0.05$, **$P < 0.01$. Values are presented as mean ± SEM. NS = not significant.

Supplemental Figure 9

Lower magnification image for Figure 2F

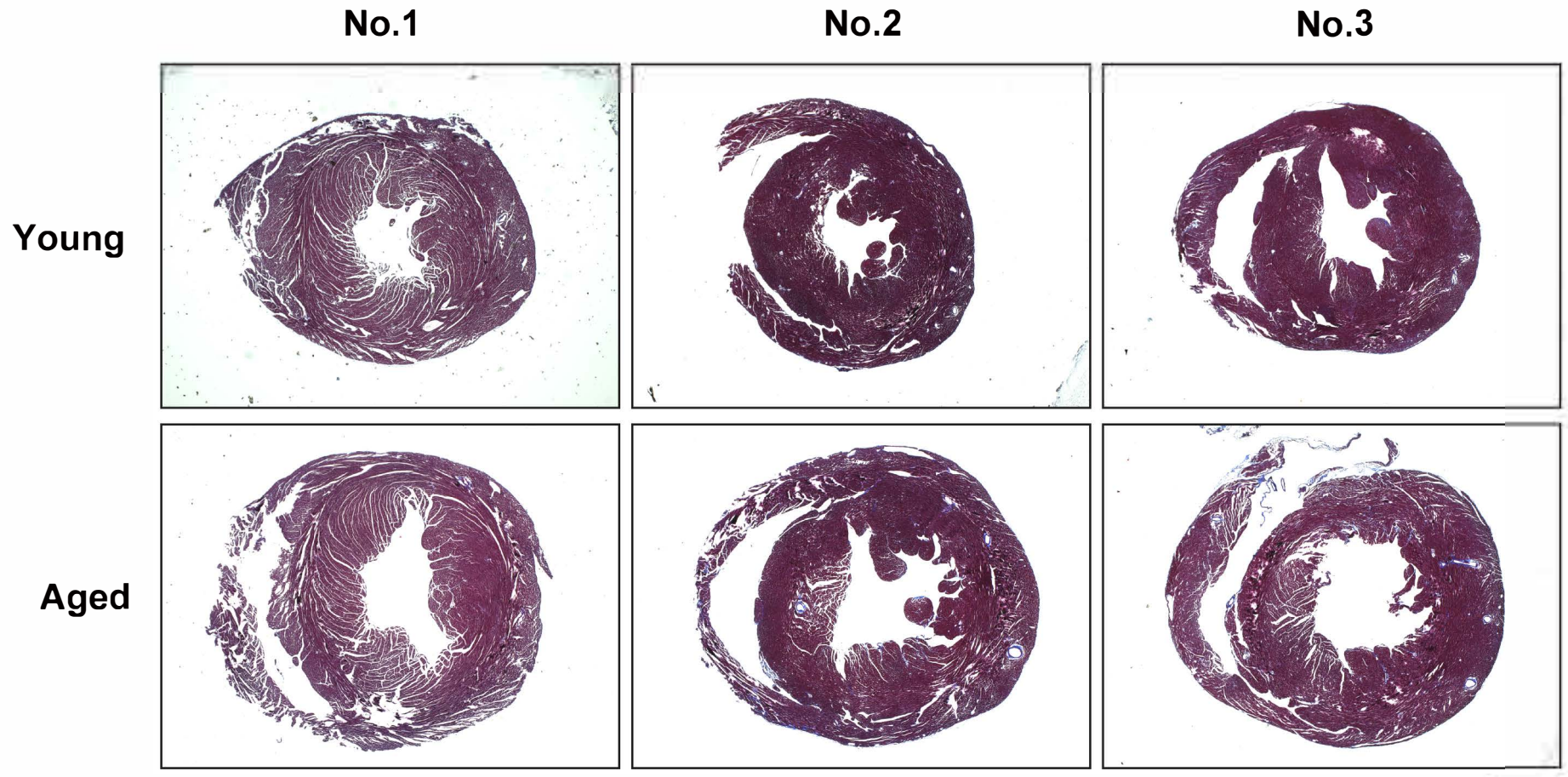

Lower magnification image for Figure 2K

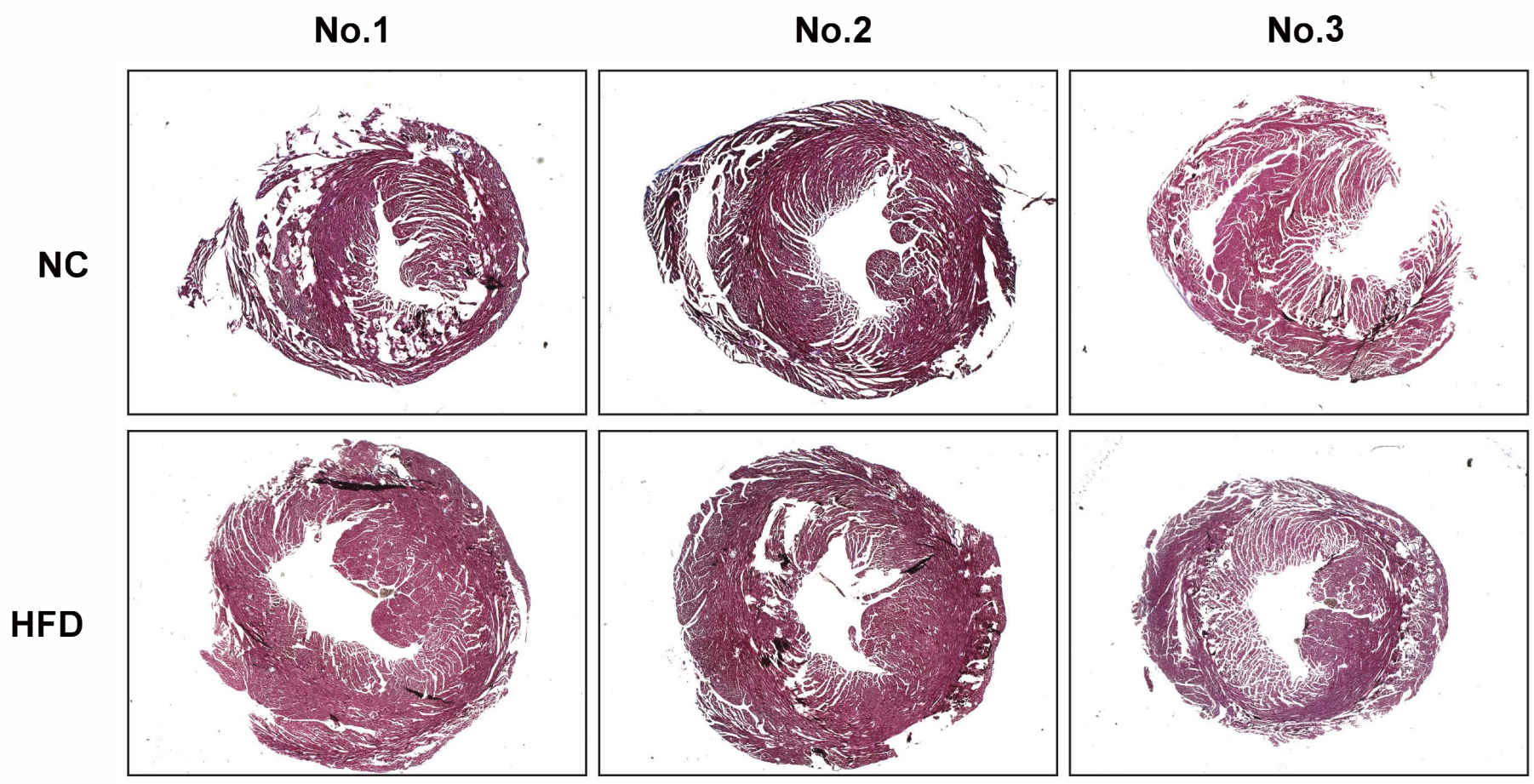

Supplemental Figure 10

Lower magnification image for Figure 3C

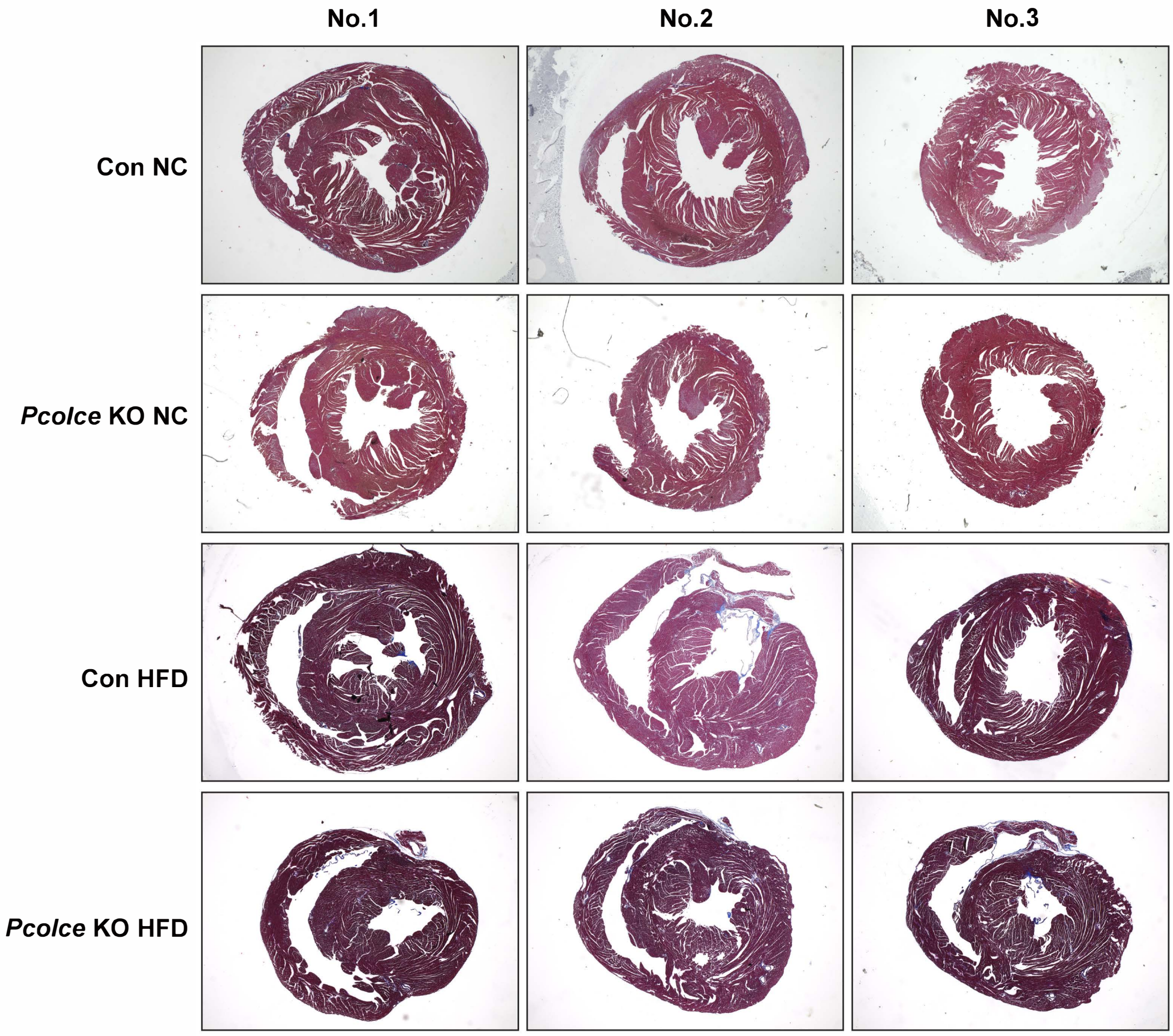

Lower magnification image for Figure 3H

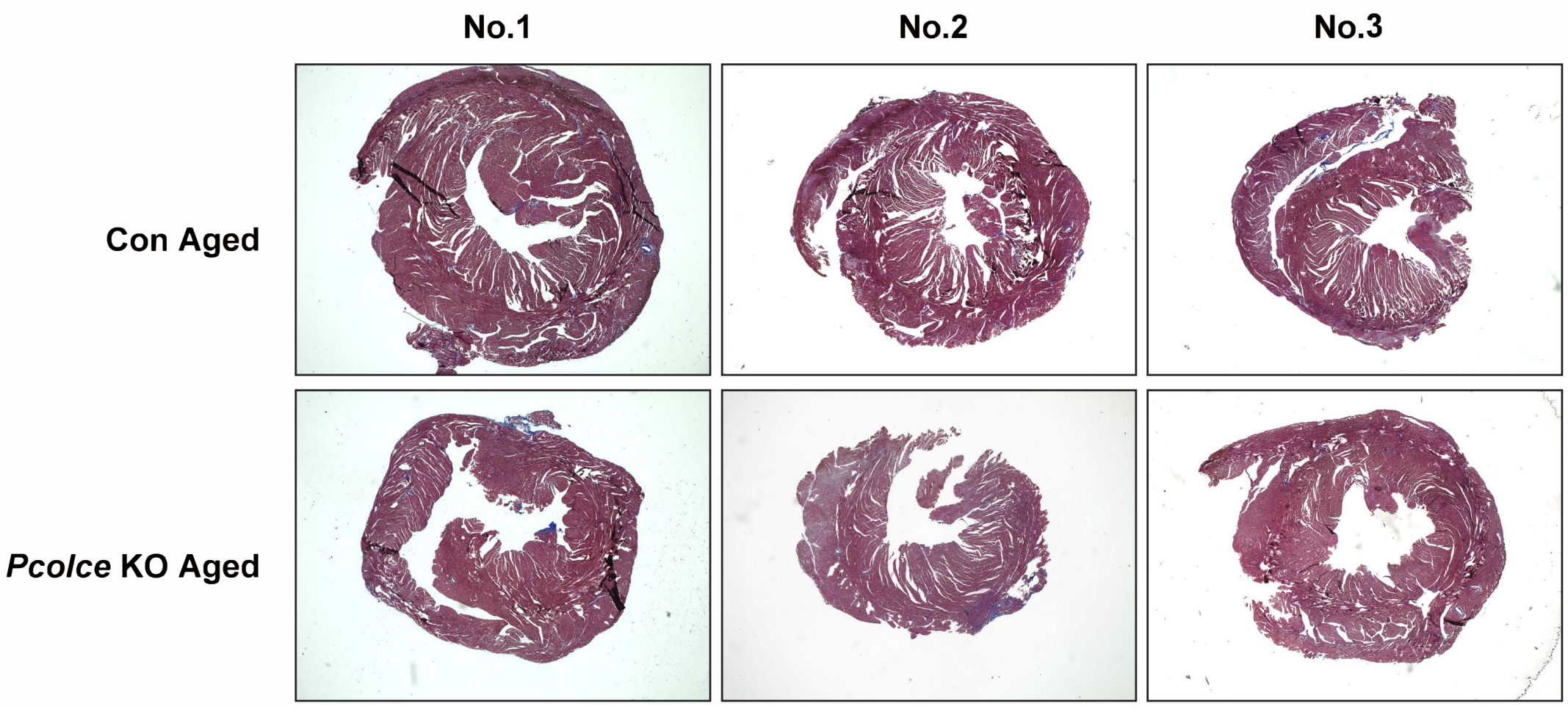

Supplemental Figure 11

Lower magnification image for Figure 4C

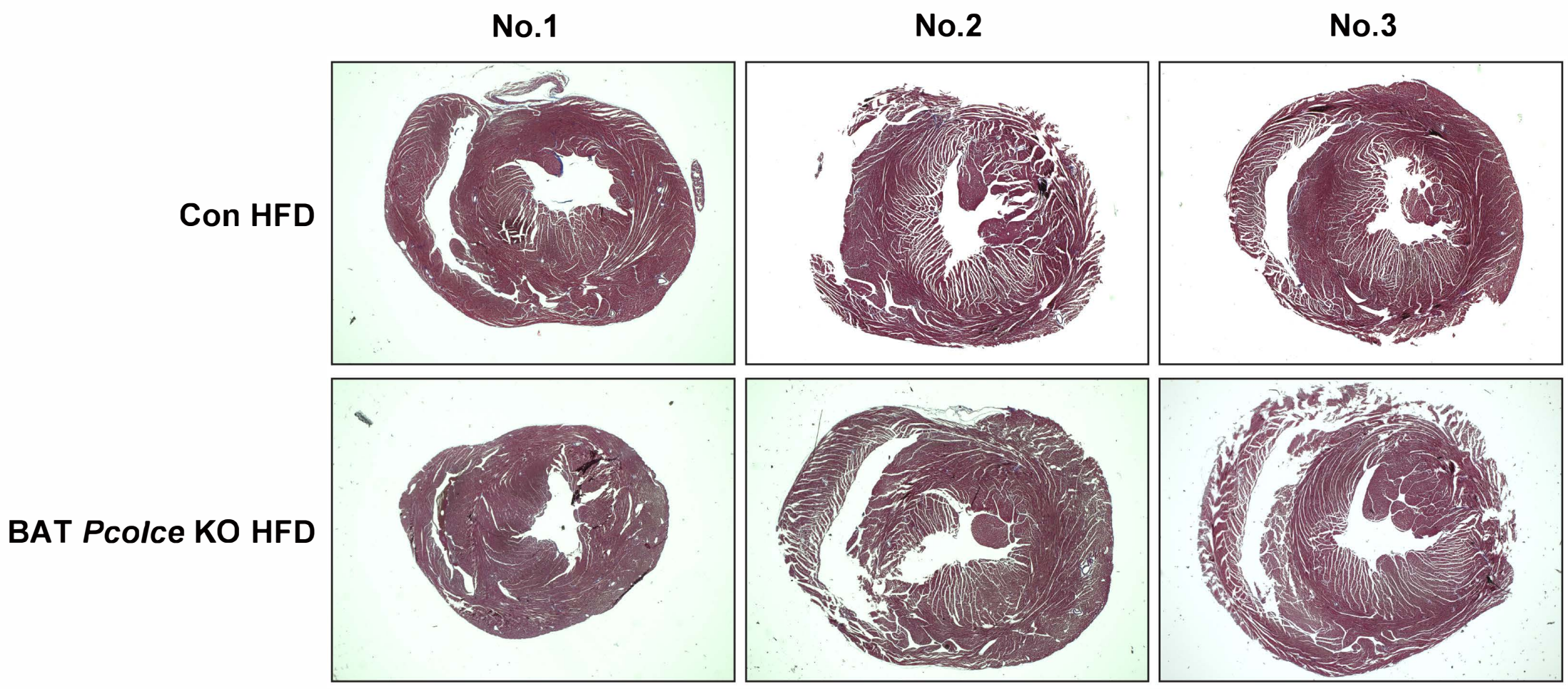

Lower magnification image for Figure 4H

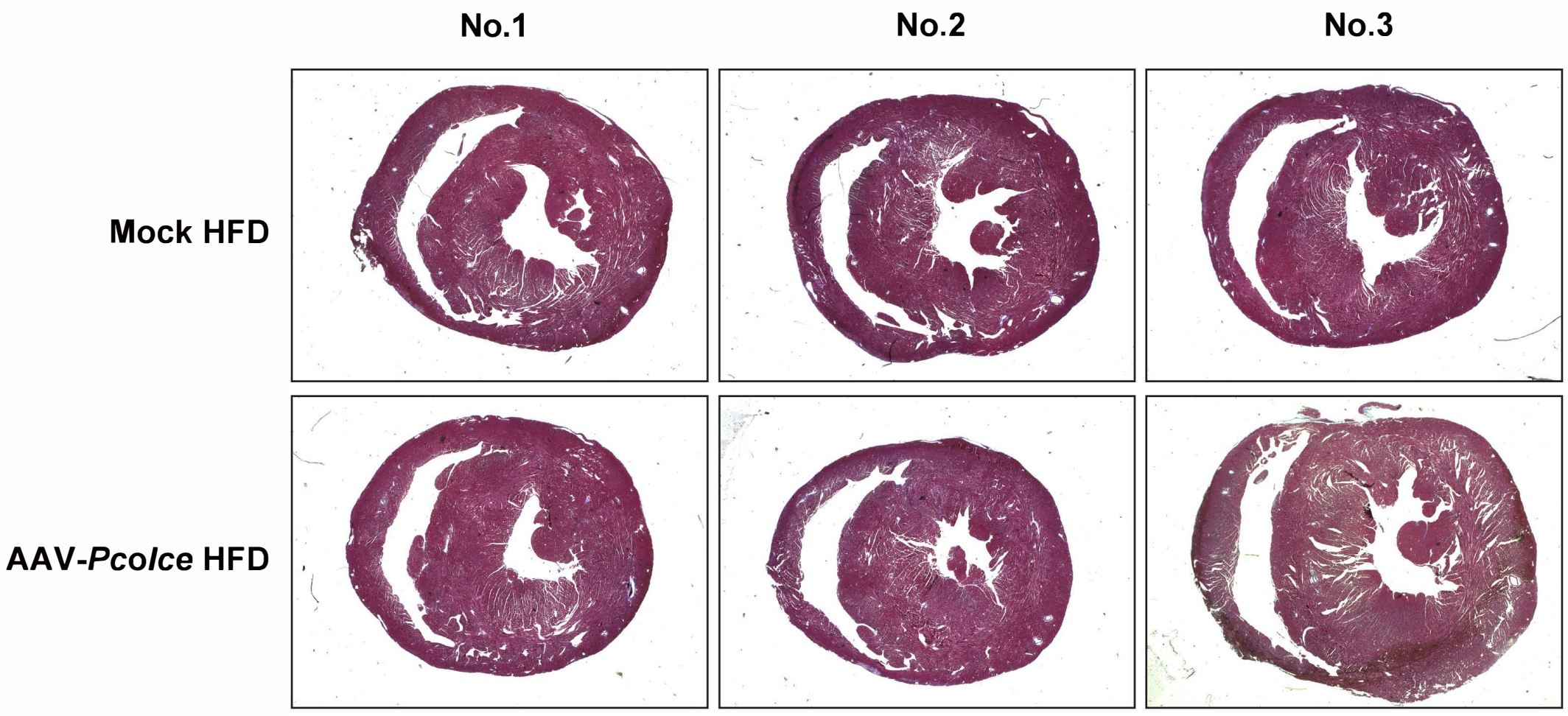

Lower magnification image for Figure 4L

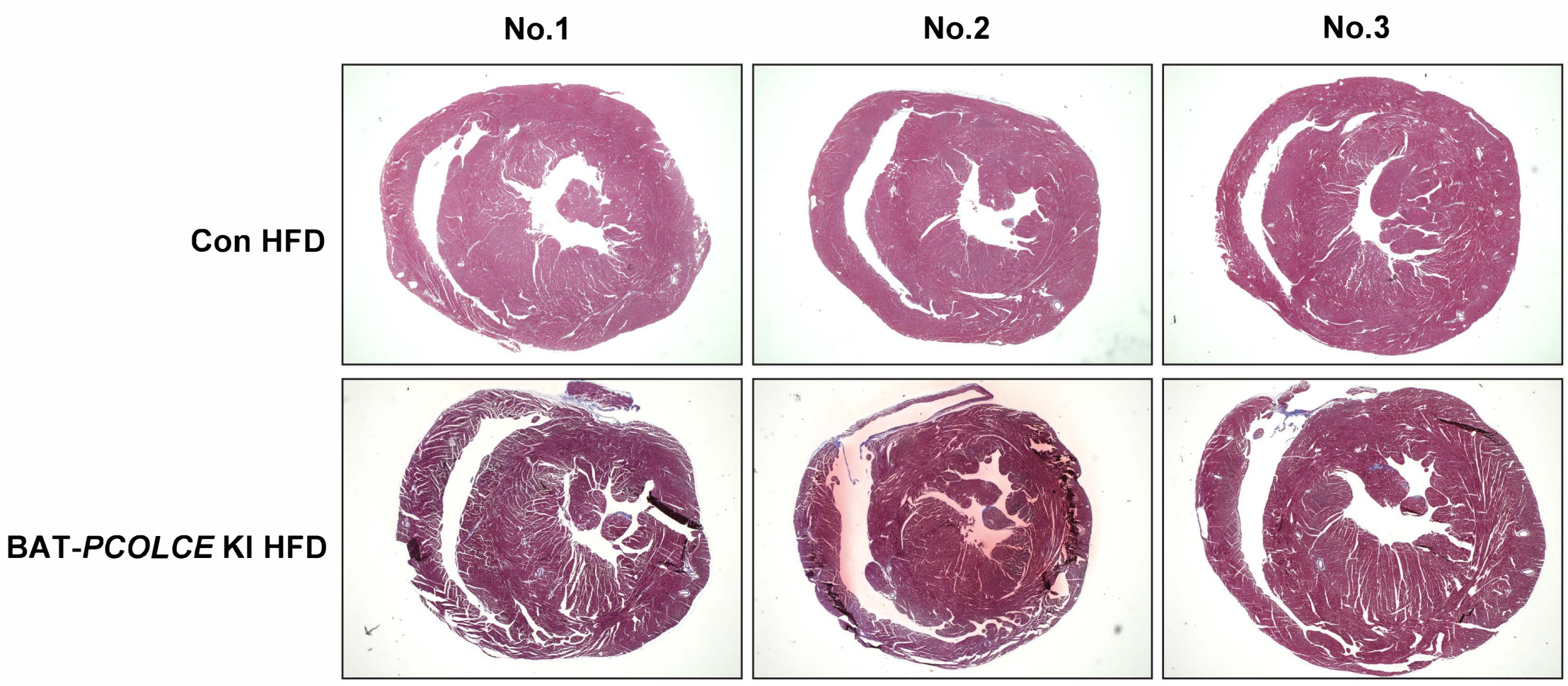

Supplemental Table 1. Multiple regression analysis testing variables correlated with plasma PCPE-1

Multiple regression analysis was performed to examine the association between plasma PCPE-1 levels and the tested variables in the samples studied in Figure 2A (n=405). Data information: Samples obtained from two biobanks were analyzed in two independent experiments.
