# Supplementary material for: PCPE-1 promotes cardiac fibrosis with aging and obesity
Source: JCI Insight. 2026 Apr 9;11(13):e195508. doi: 10.1172/jci.insight.195508 (PMC13460844; doi:10.1172/jci.insight.195508)

Supplemental material 1

Full unedited blot for Figure 1C

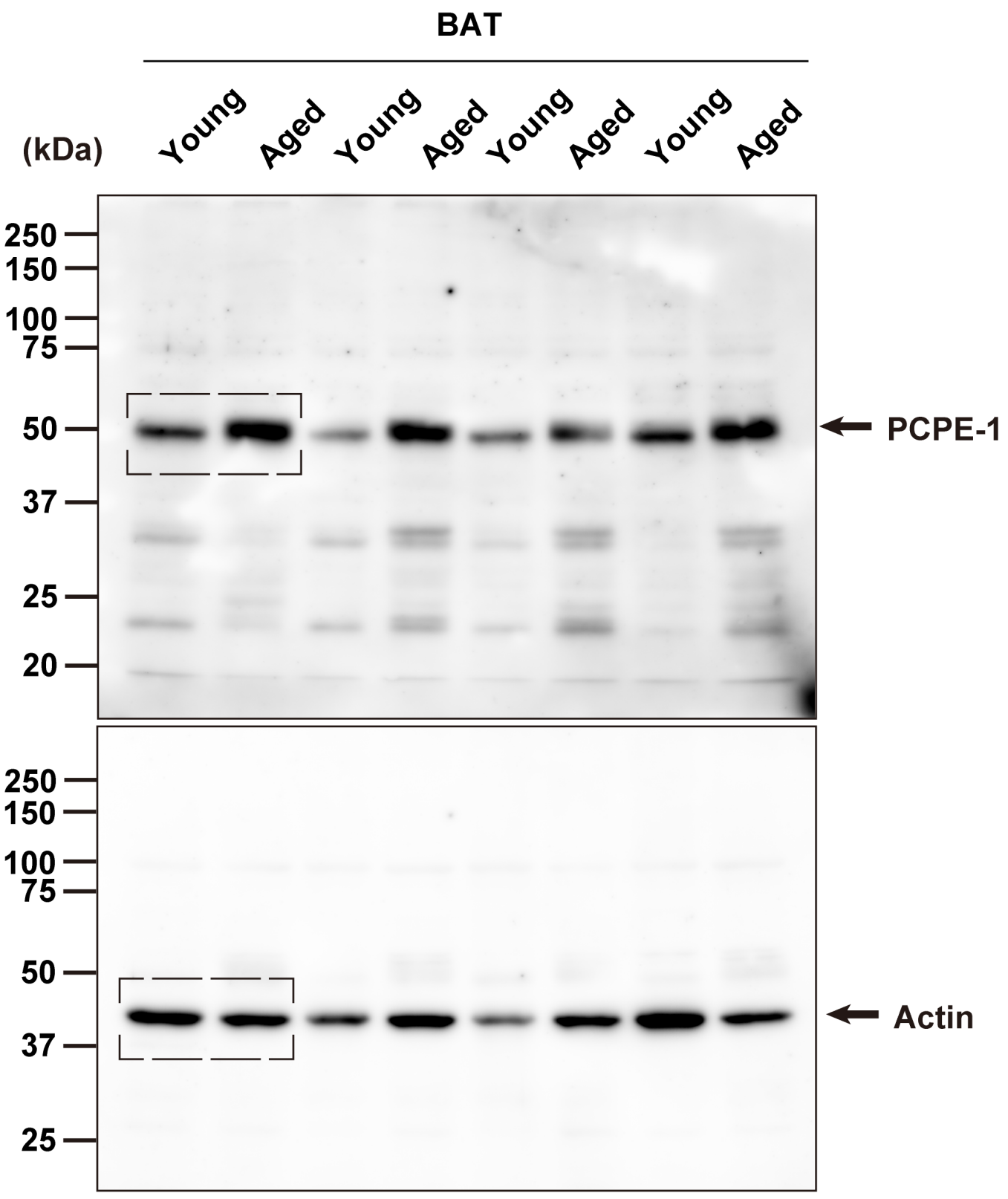

Full unedited blot for Figure 1G

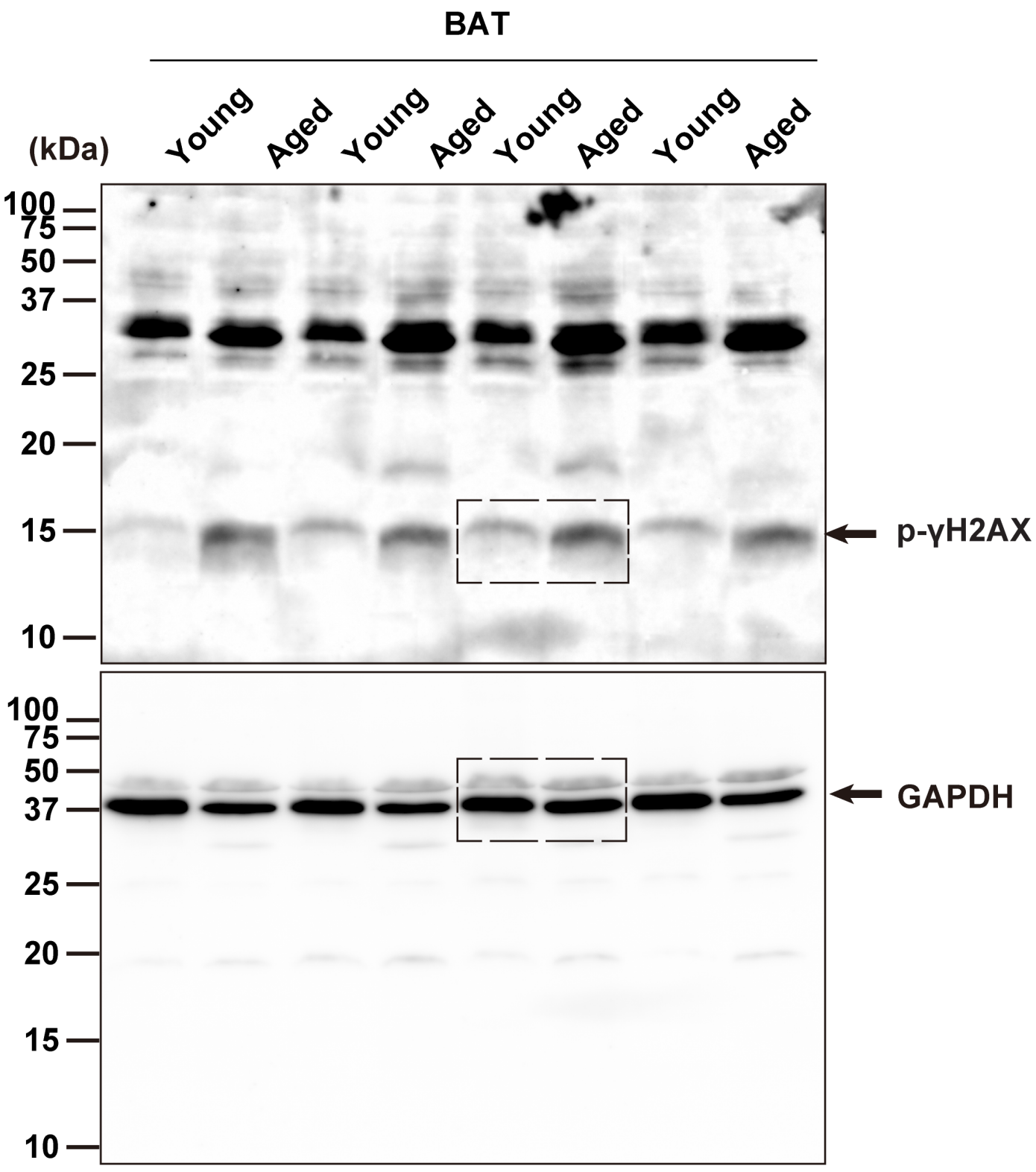

Full unedited blot for Figure 1H

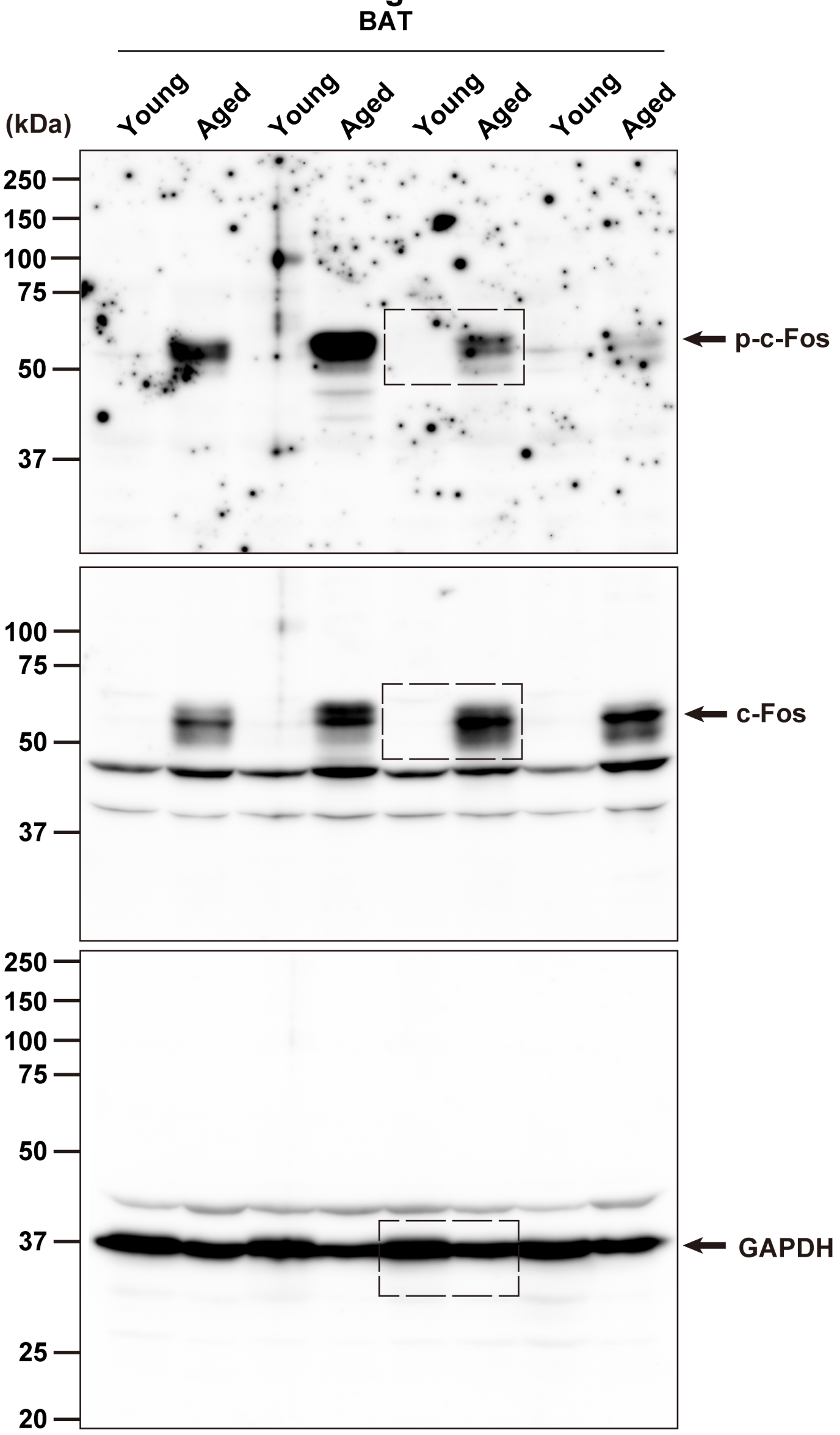

Full unedited blot for Figure1I

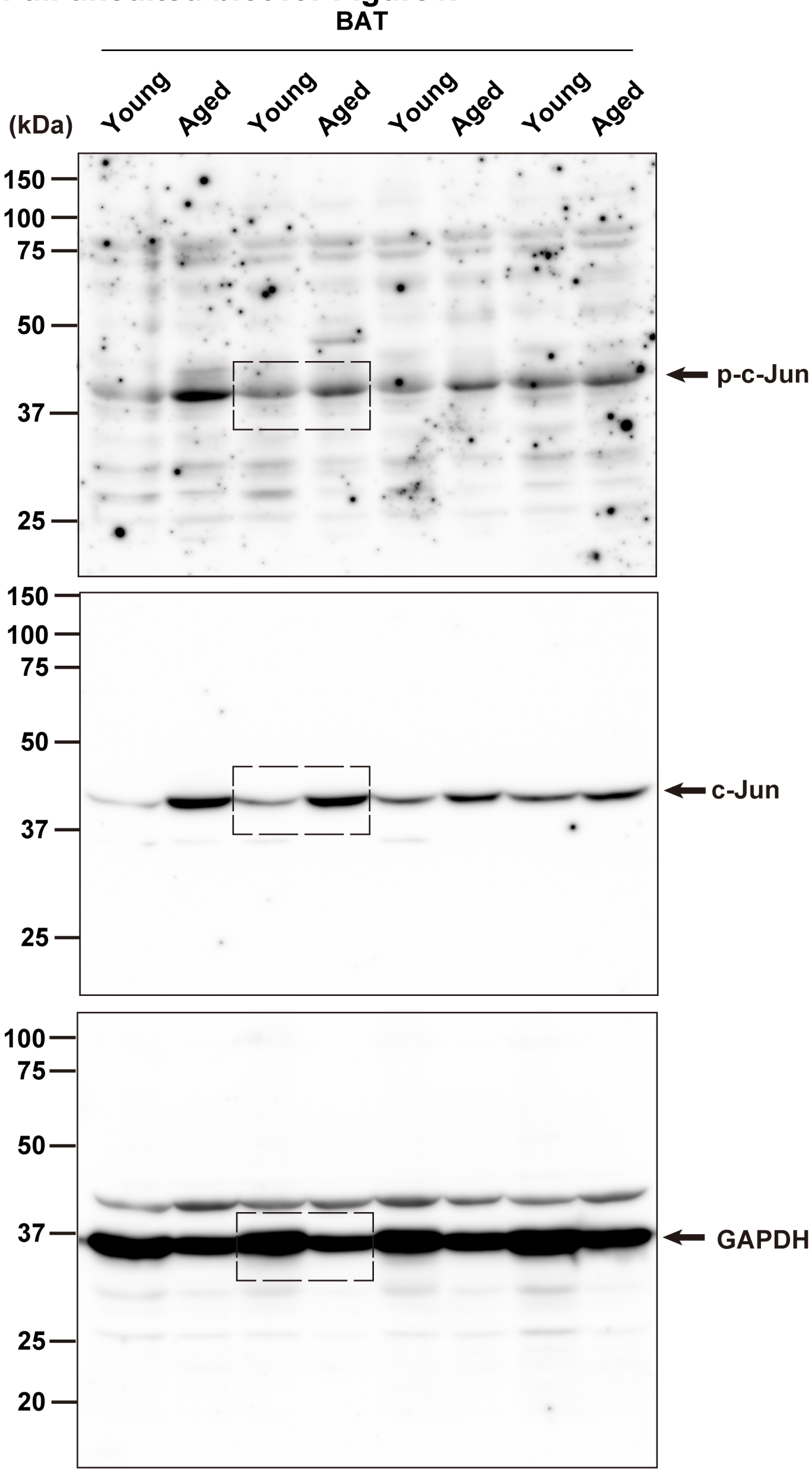

Supplemental material 2

Full unedited blot for Figure 1K

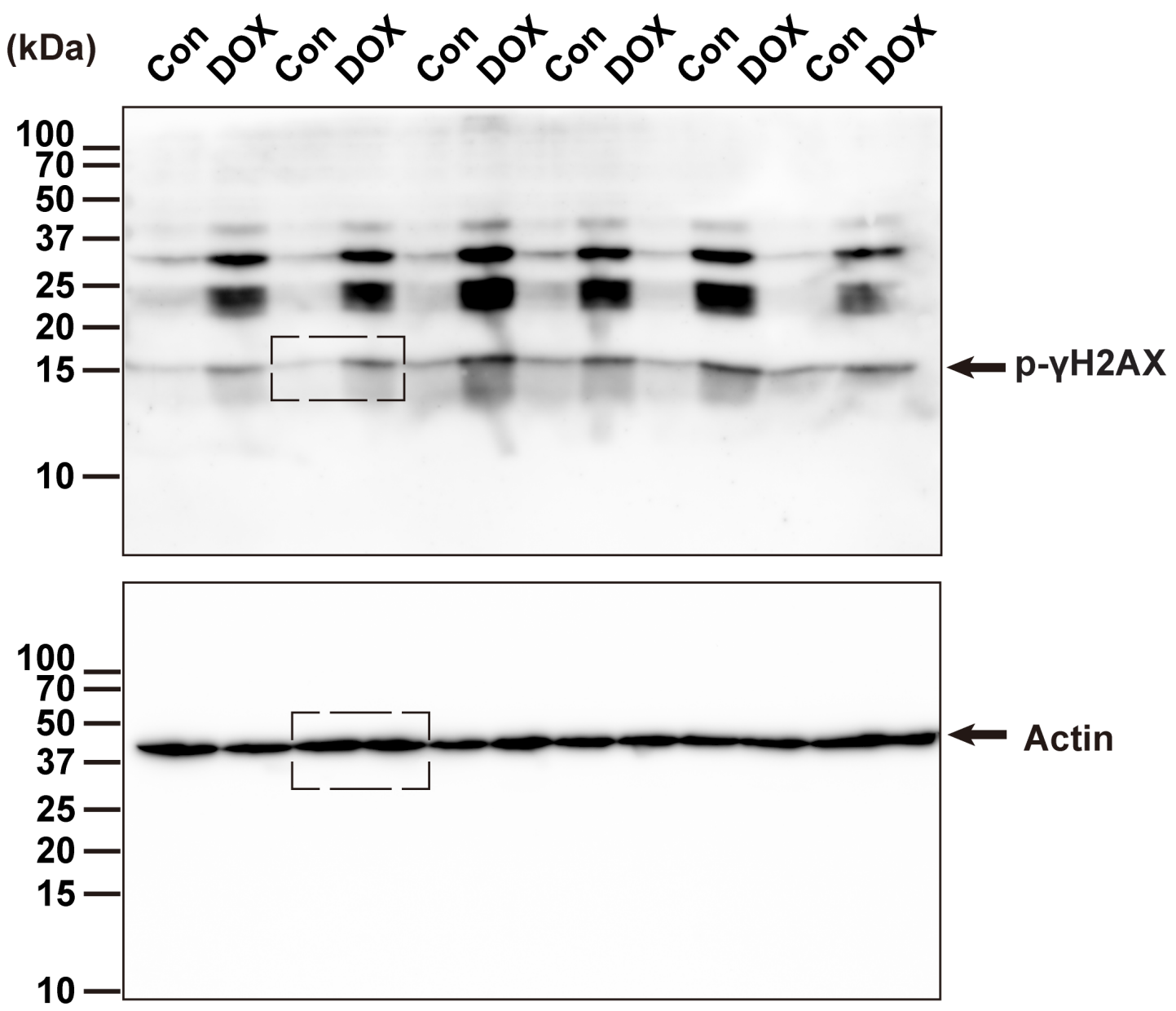

Full unedited blot for Figure 1L

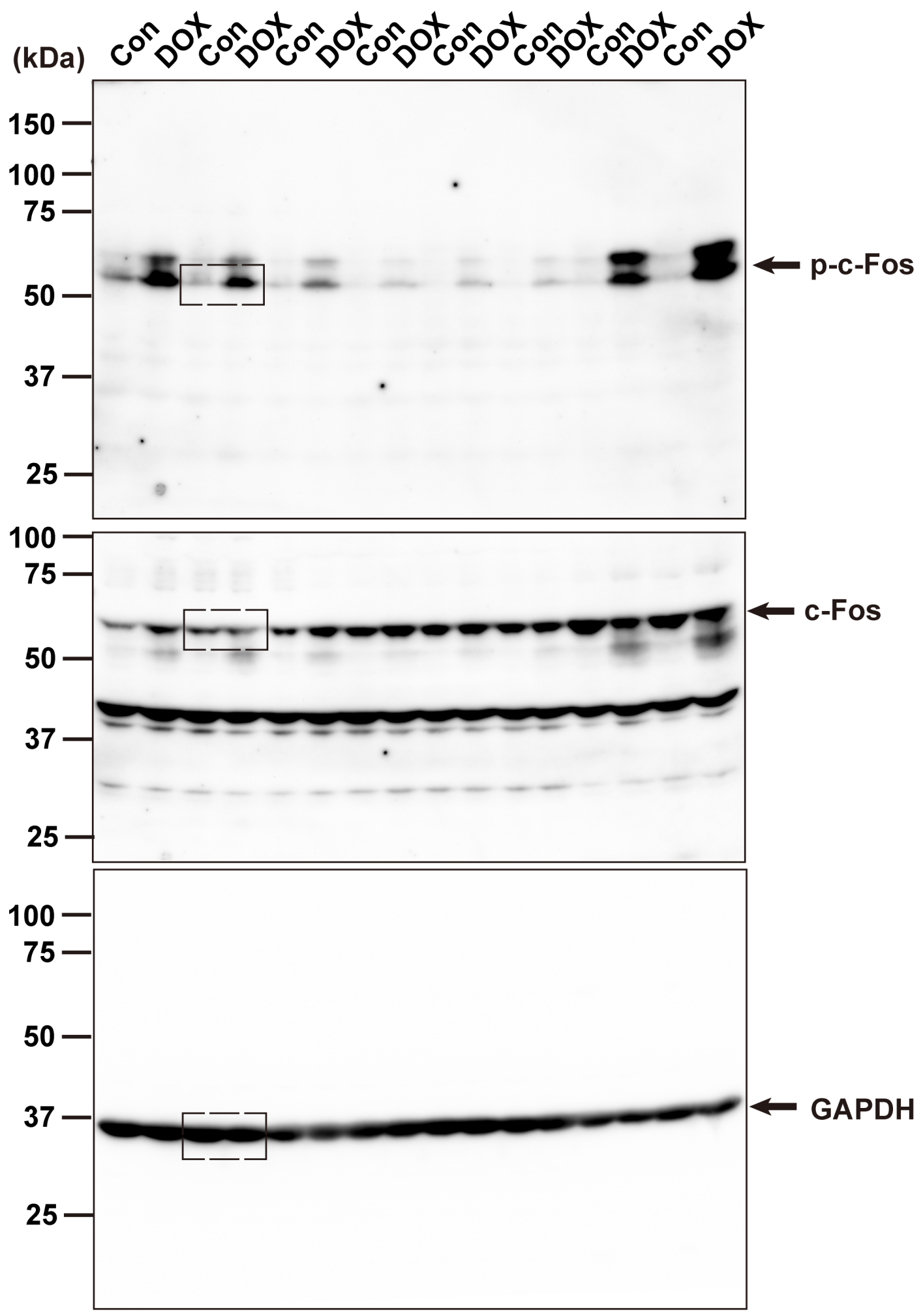

Full unedited blot for Figure 1M

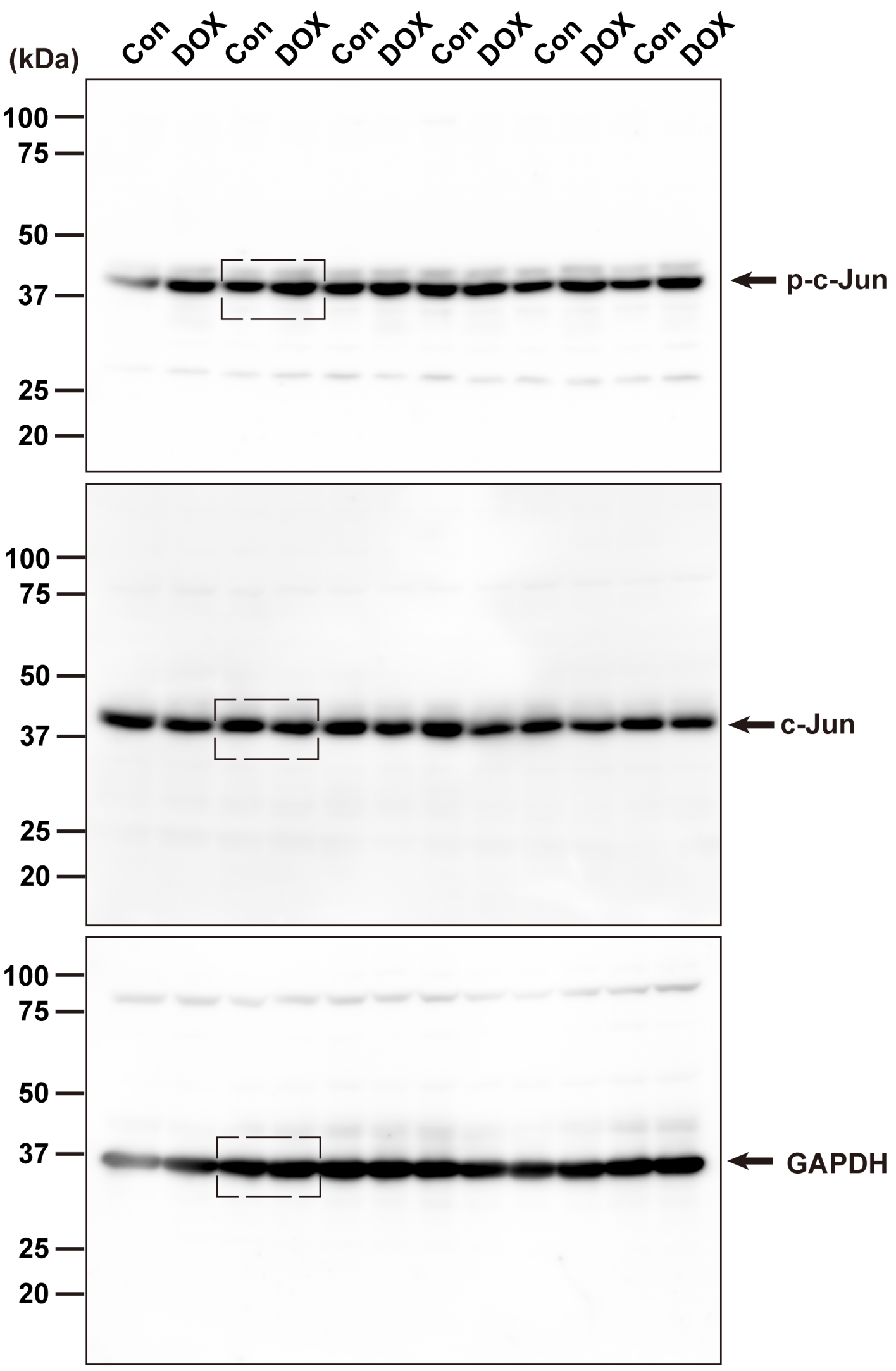

Full unedited blot for Figure 1Q

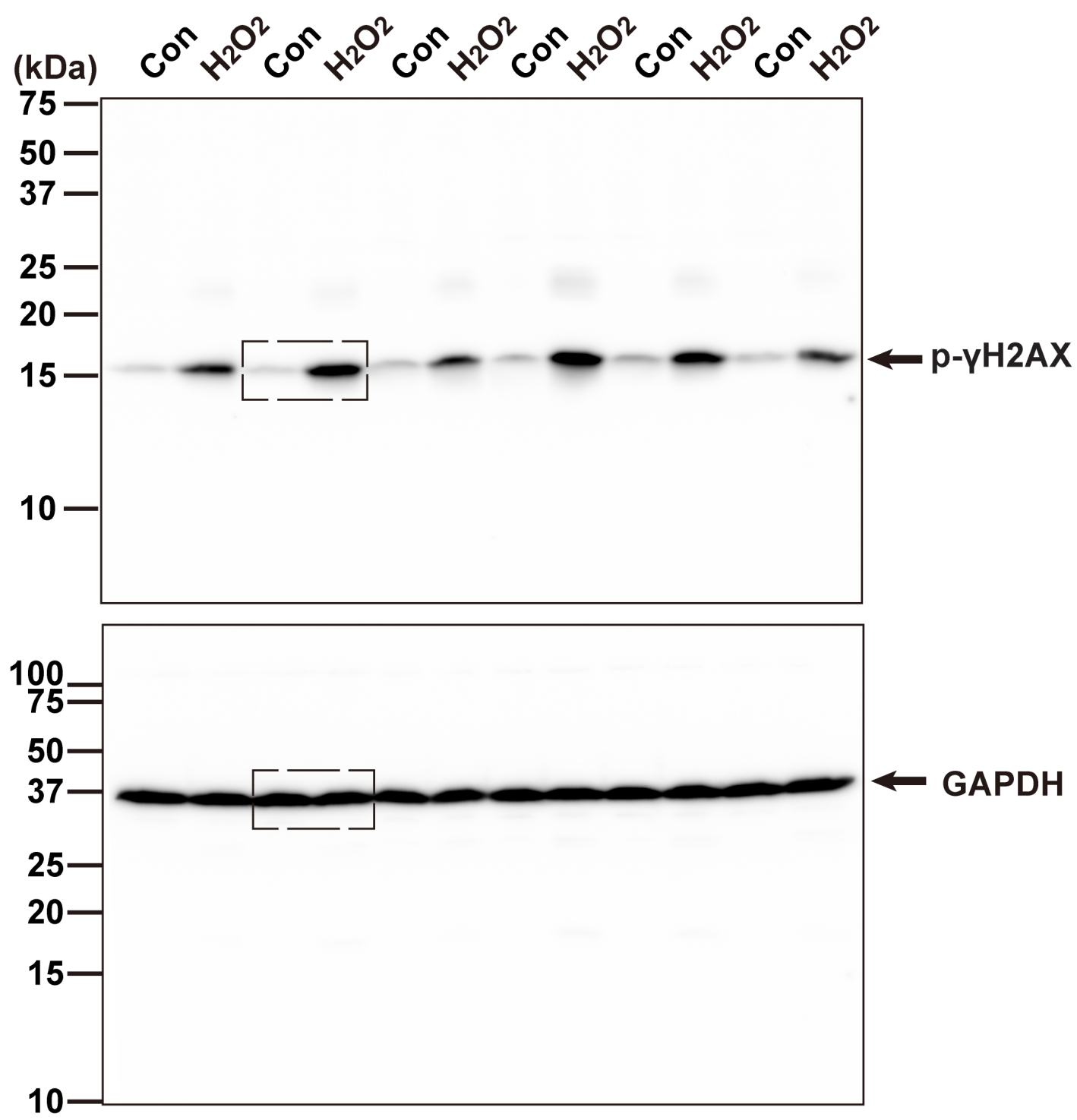

Supplemental material 3

Full unedited blot for Figure 1R

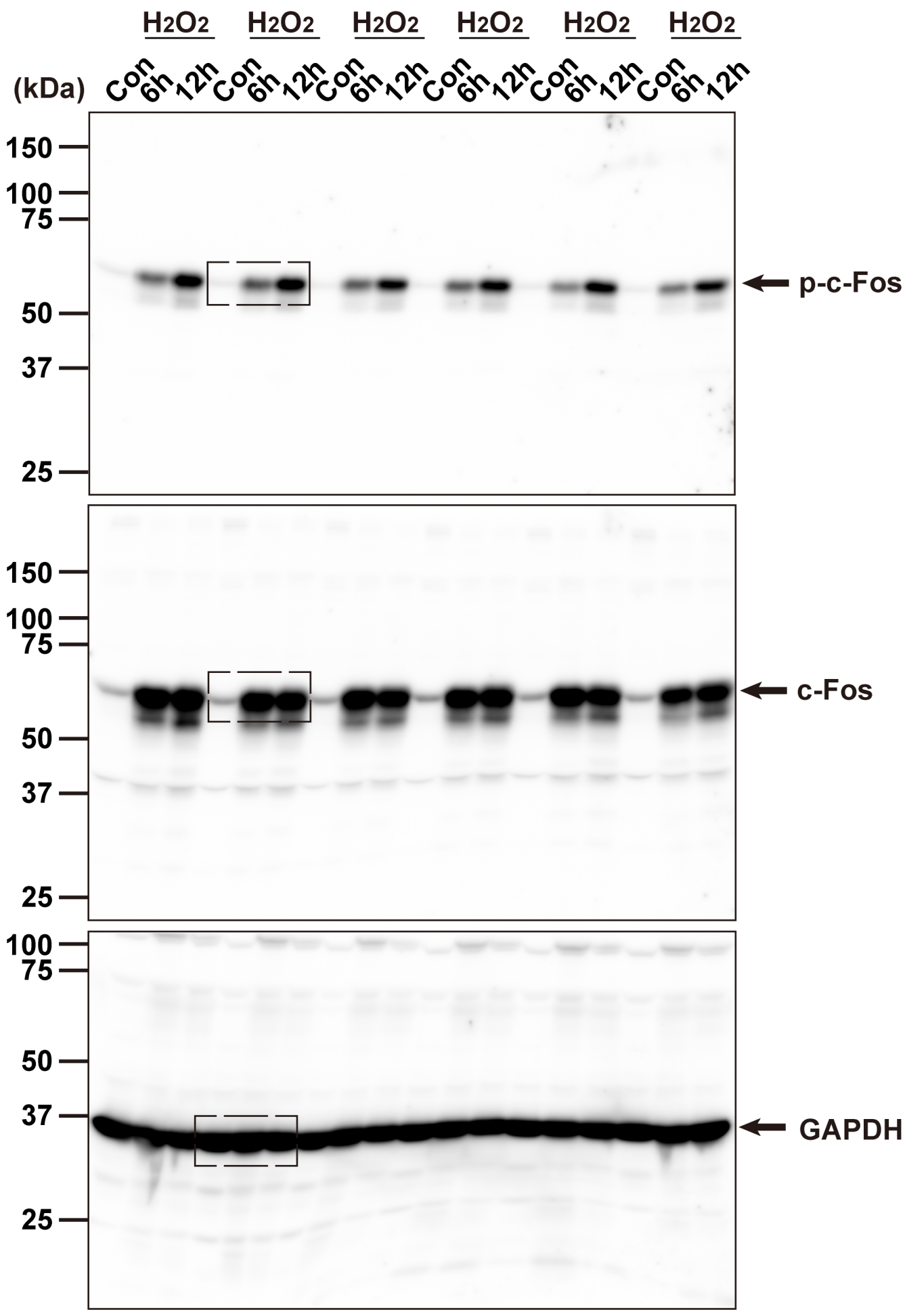

Full unedited blot for Figure 1S

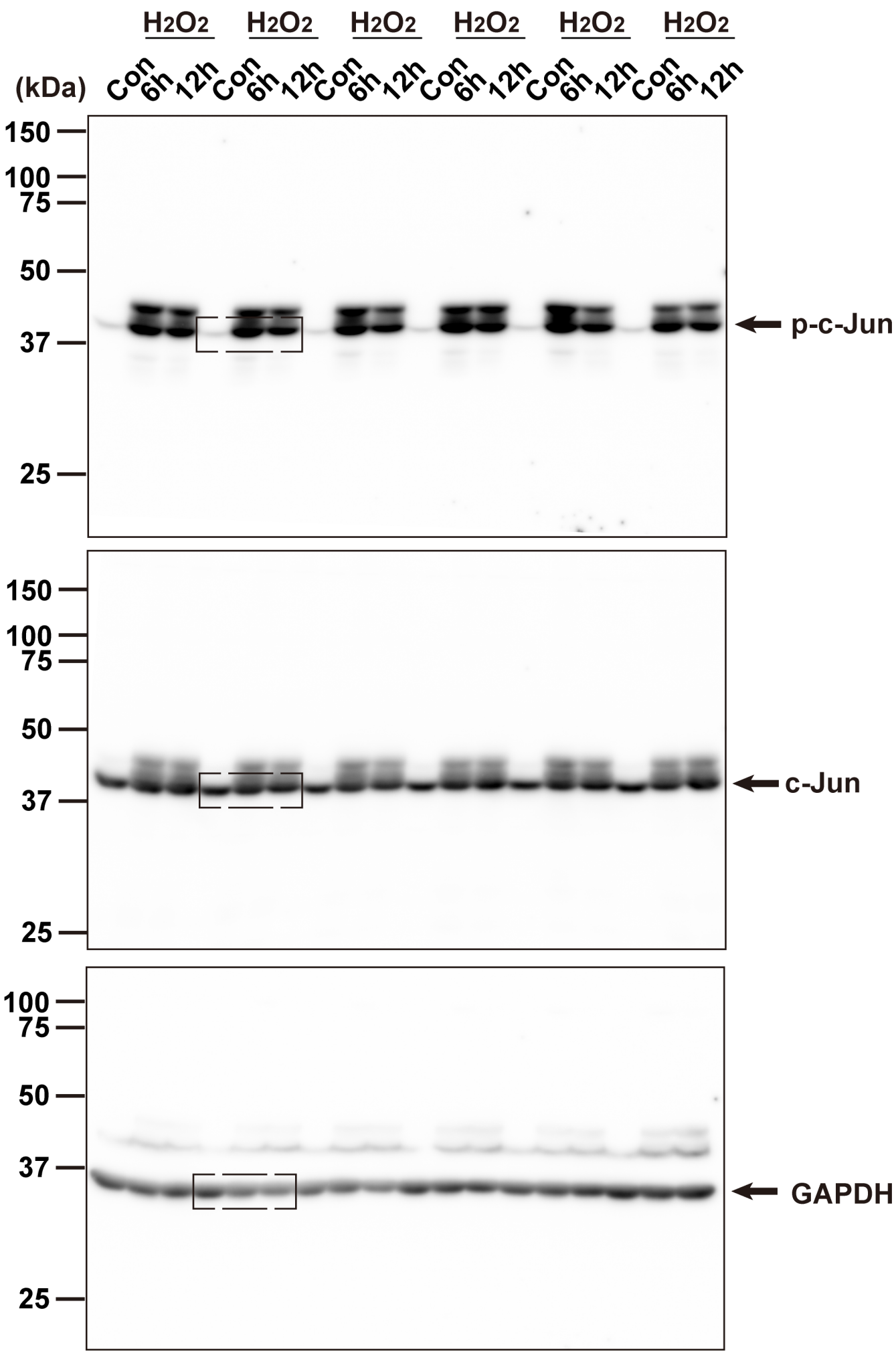

Supplemental material 4

Full unedited blot for Figure 2D

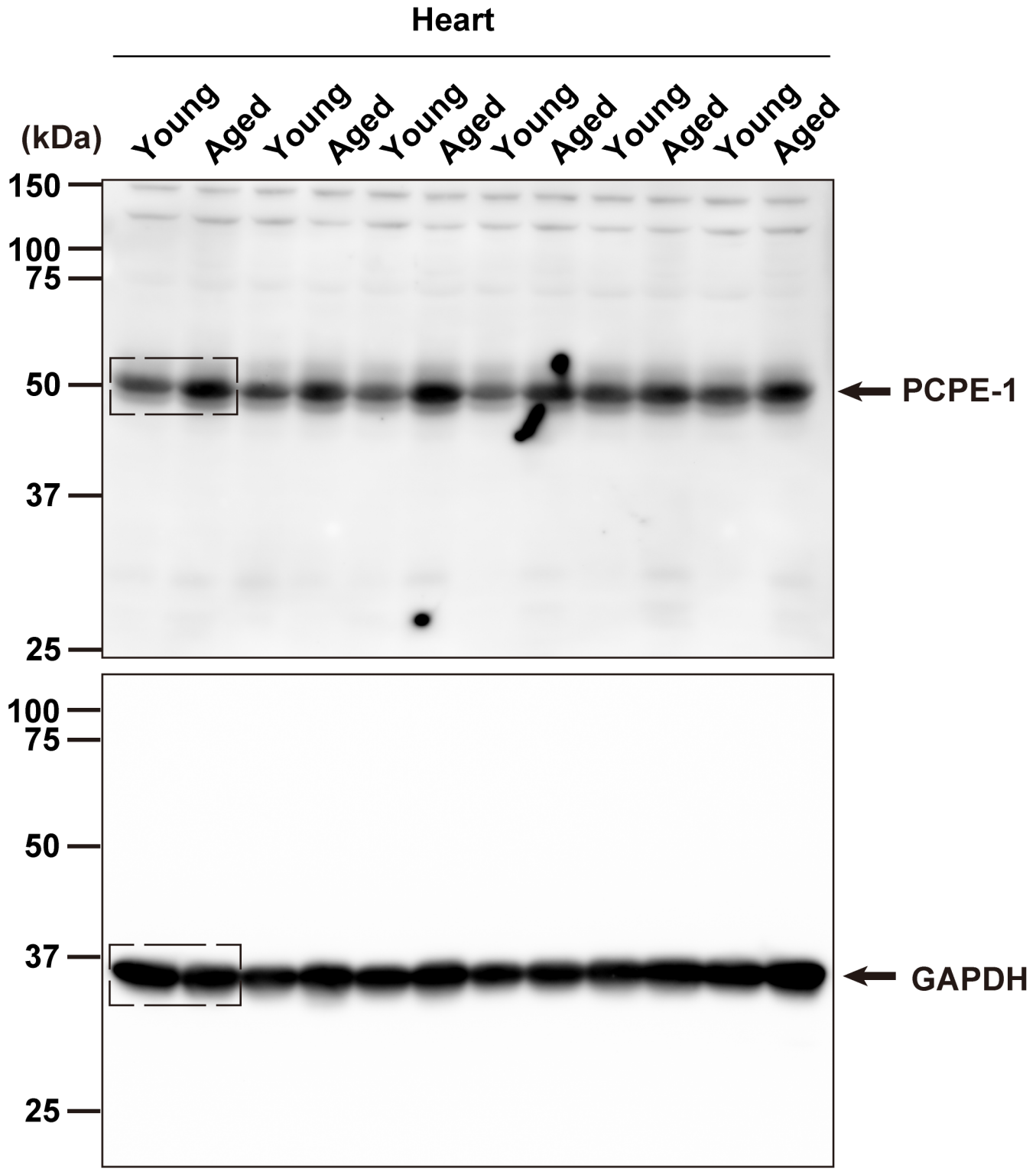

Full unedited blot for Figure 2I

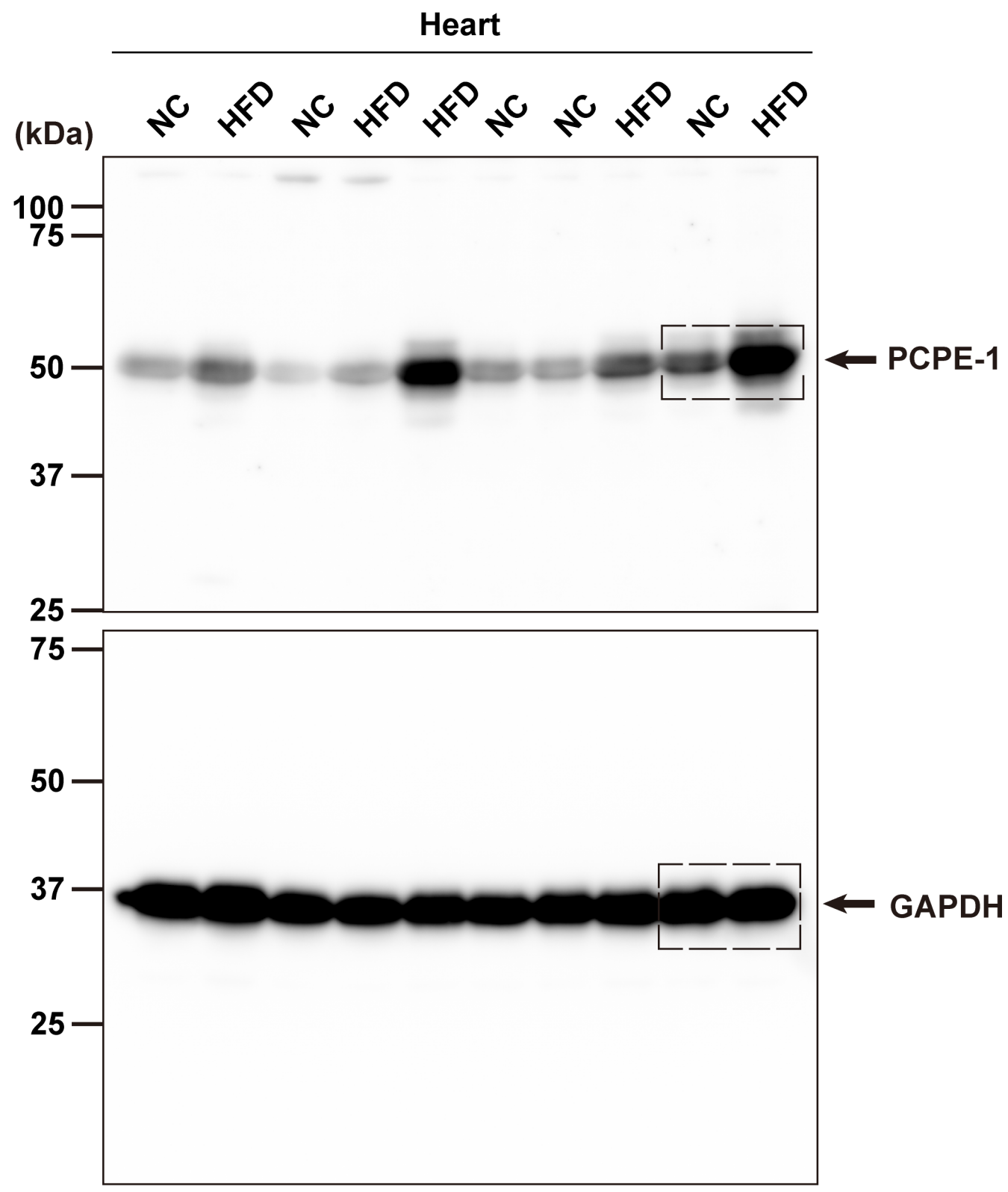

Supplemental material 5

Full unedited blot for Figure 3A

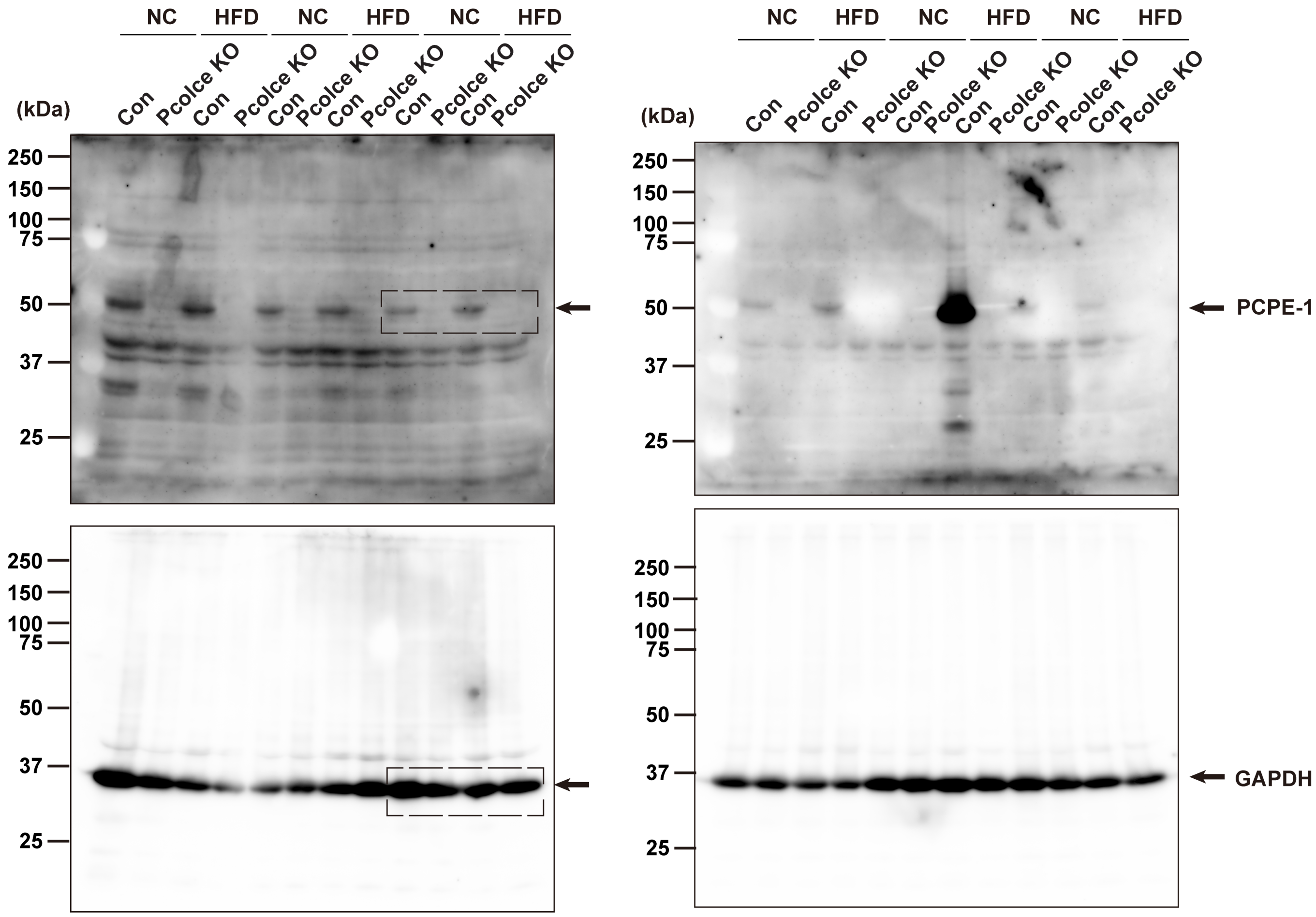

Full unedited blot for Figure 3F

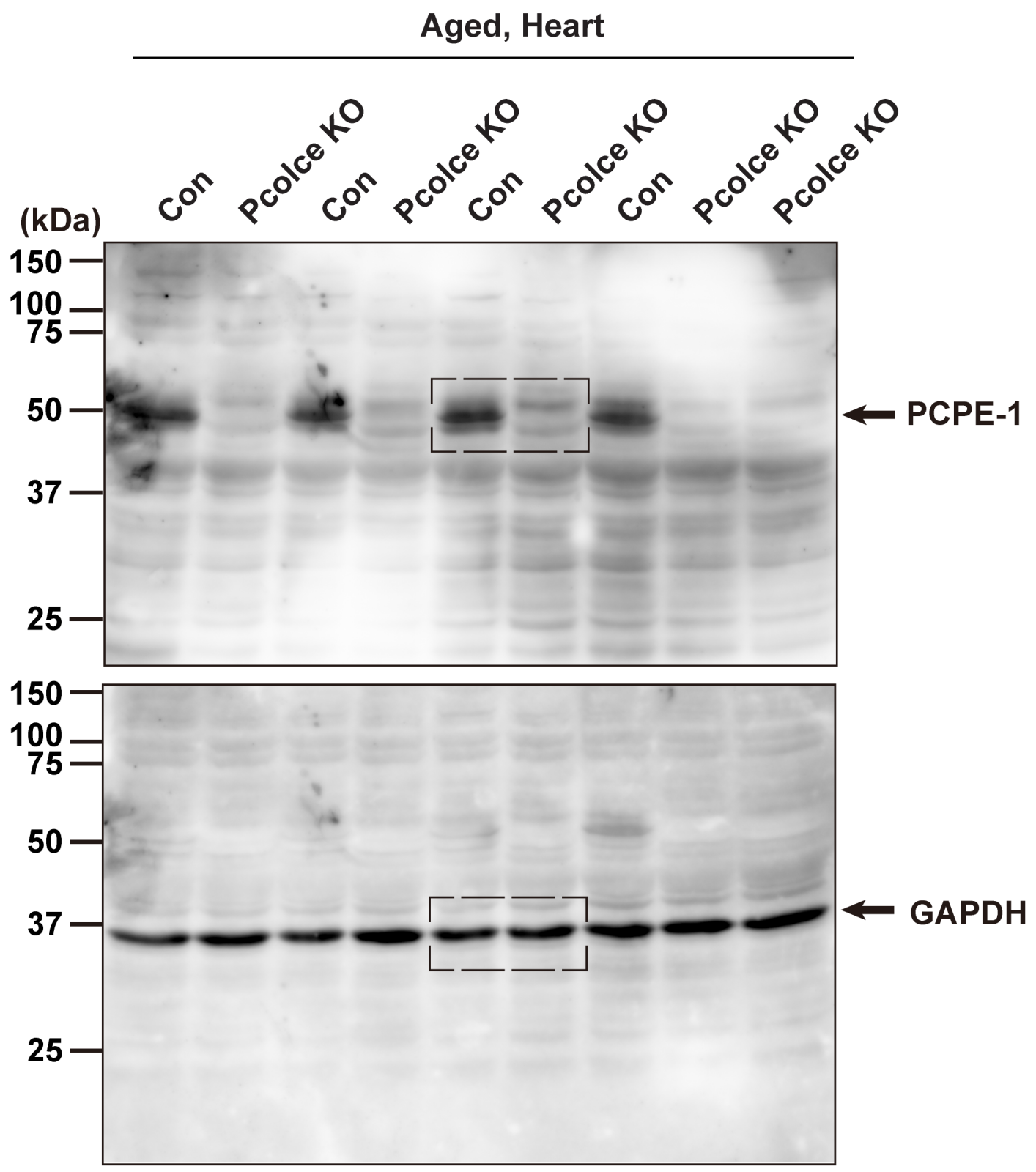

Supplemental material 6

Full unedited blot for Figure 4A

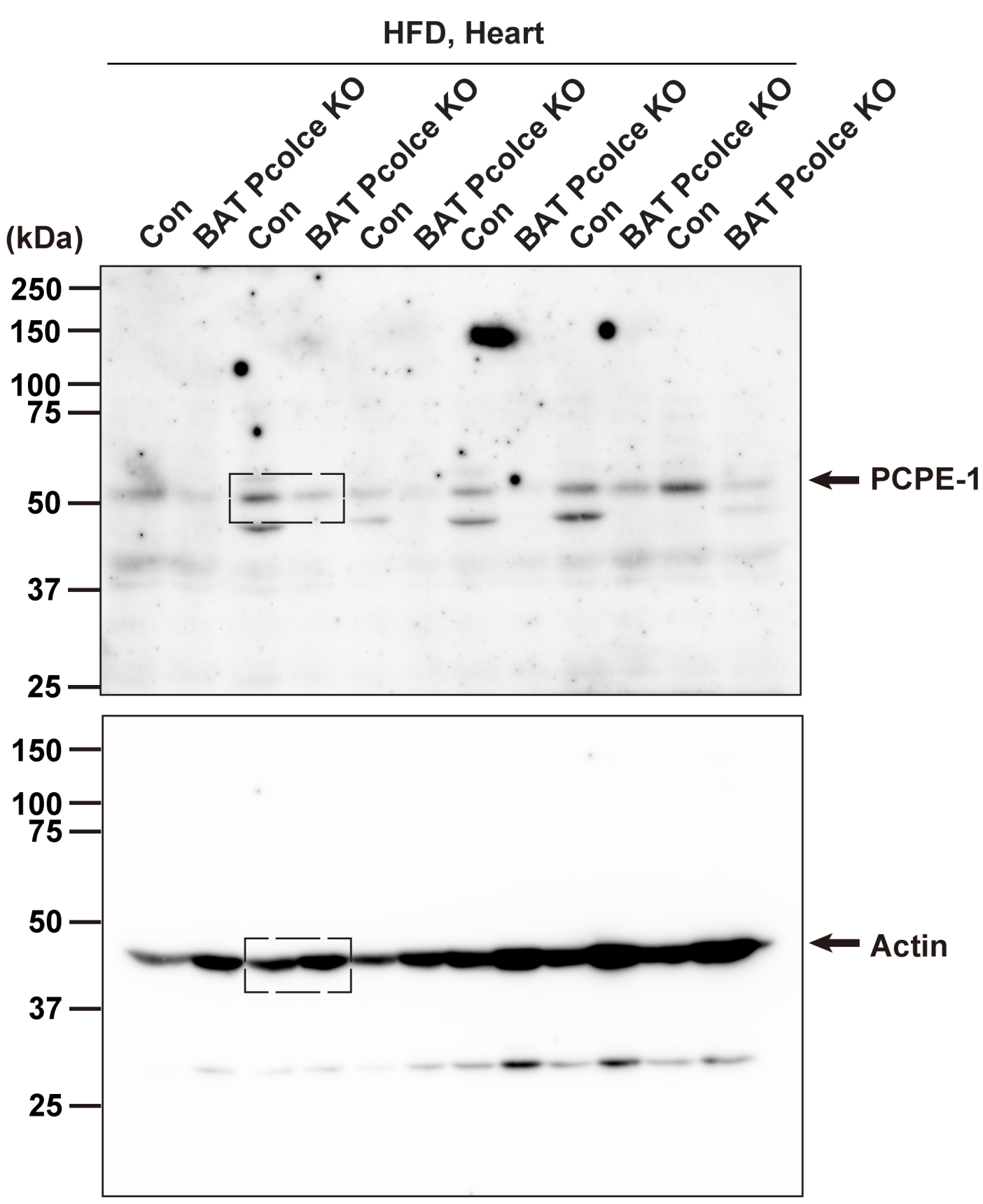

Full unedited blot for Figure 4F

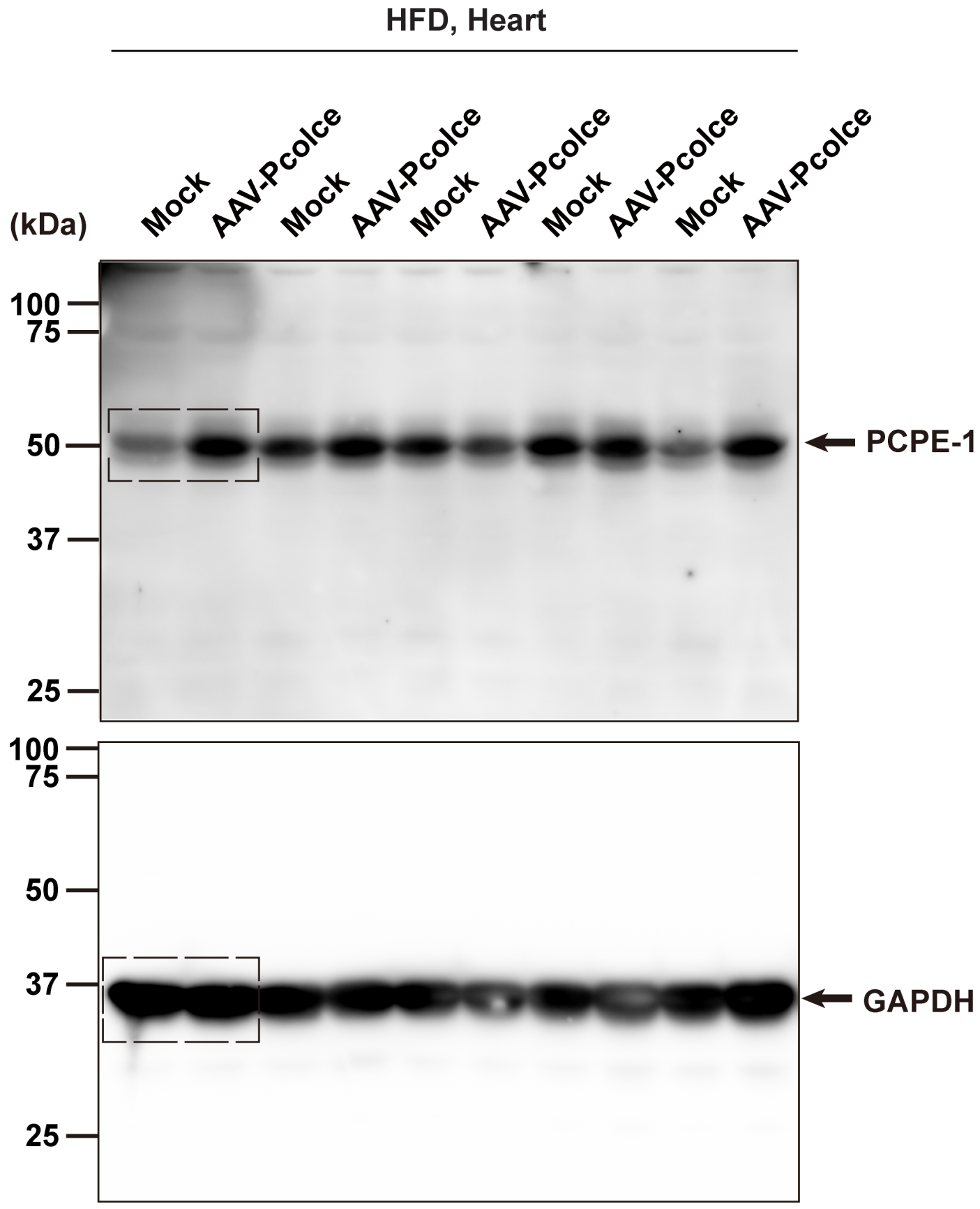

Full unedited blot for Figure 4J

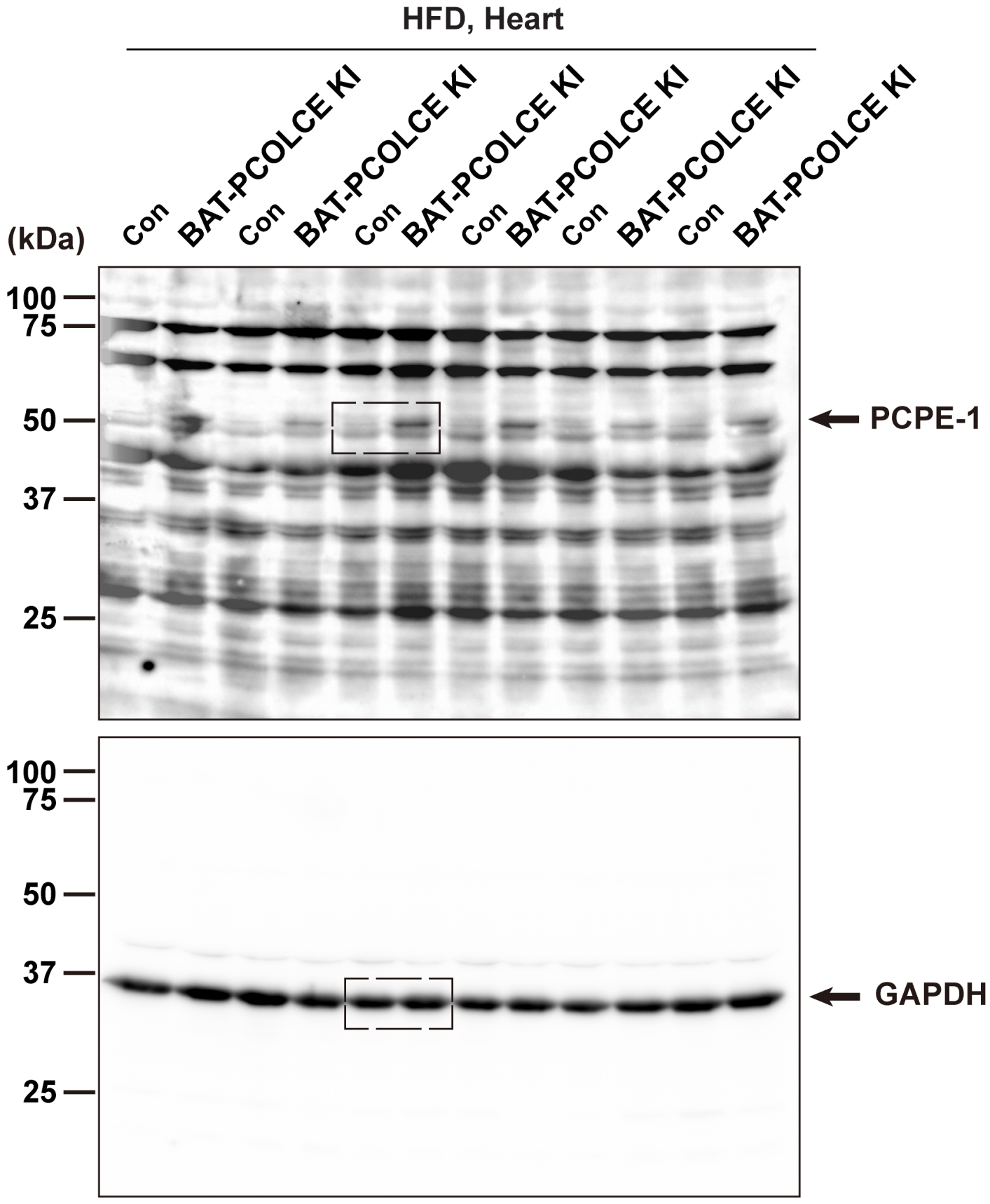

Supplemental material 7

Full unedited blot for Supplemental Figure 1G

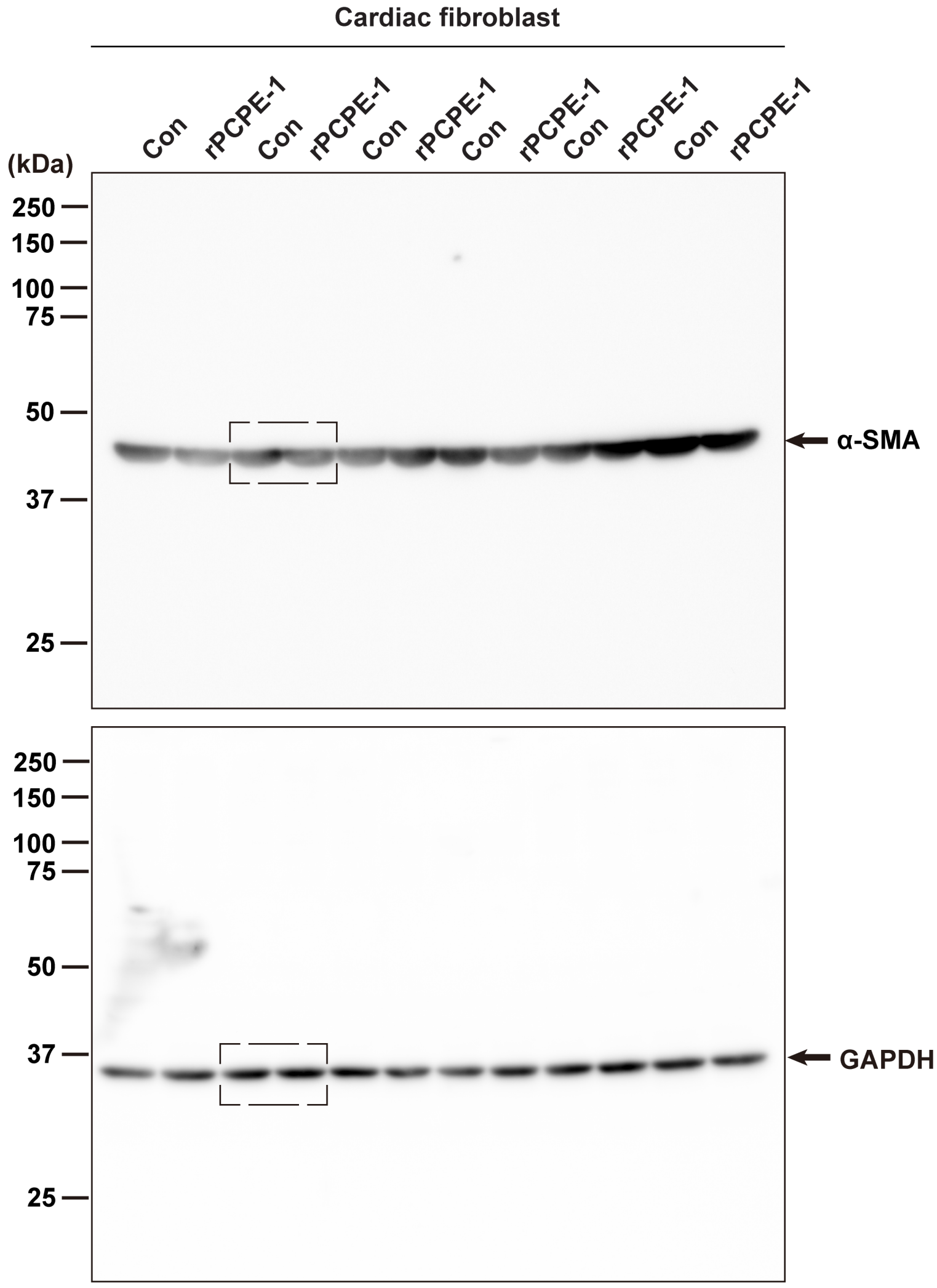

Full unedited blot for Supplemental Figure 3F

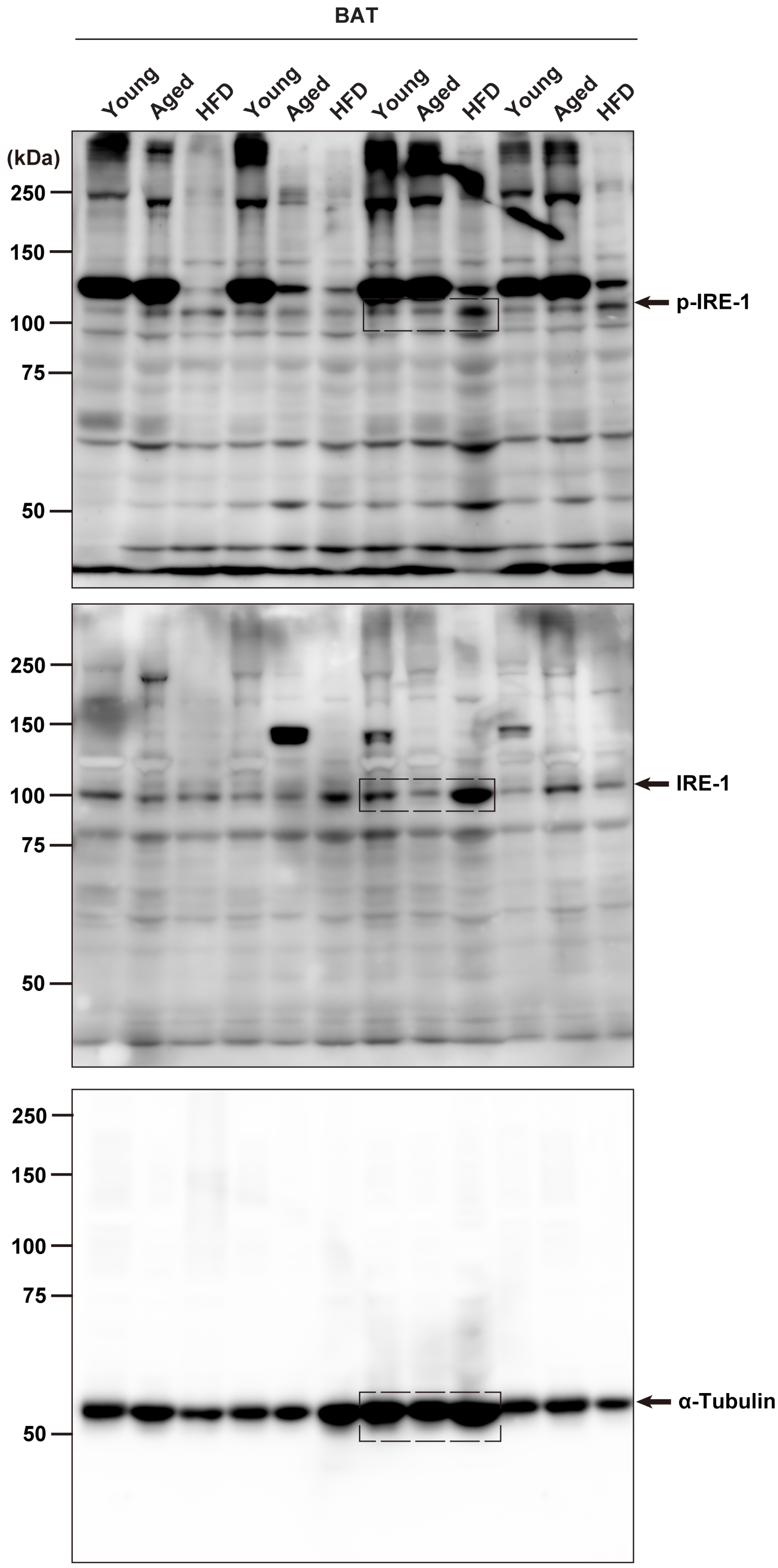

Supplemental material 8

Full unedited blot for Supplemental Figure 4M

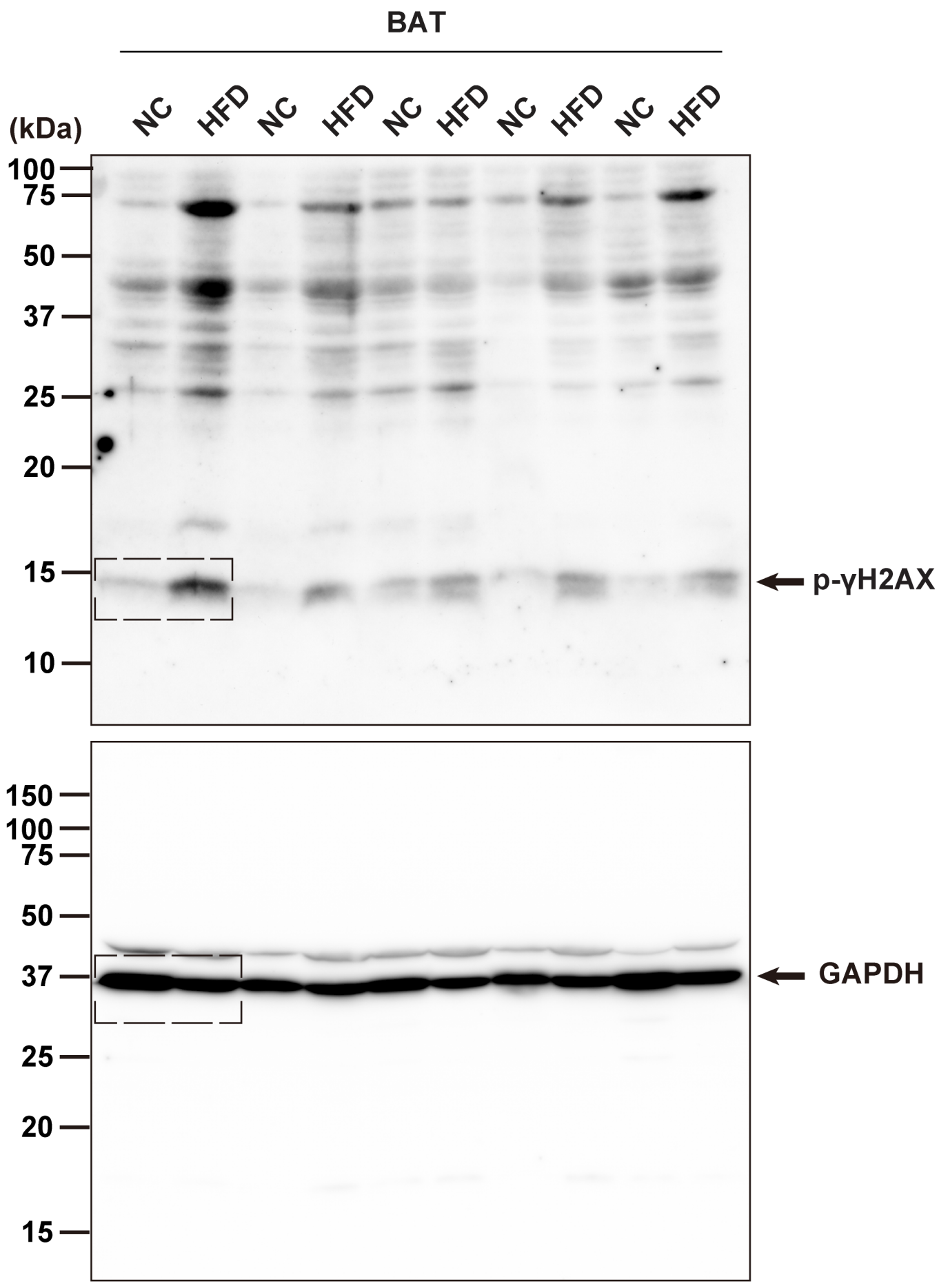

Full unedited blot for Supplemental Figure 7B

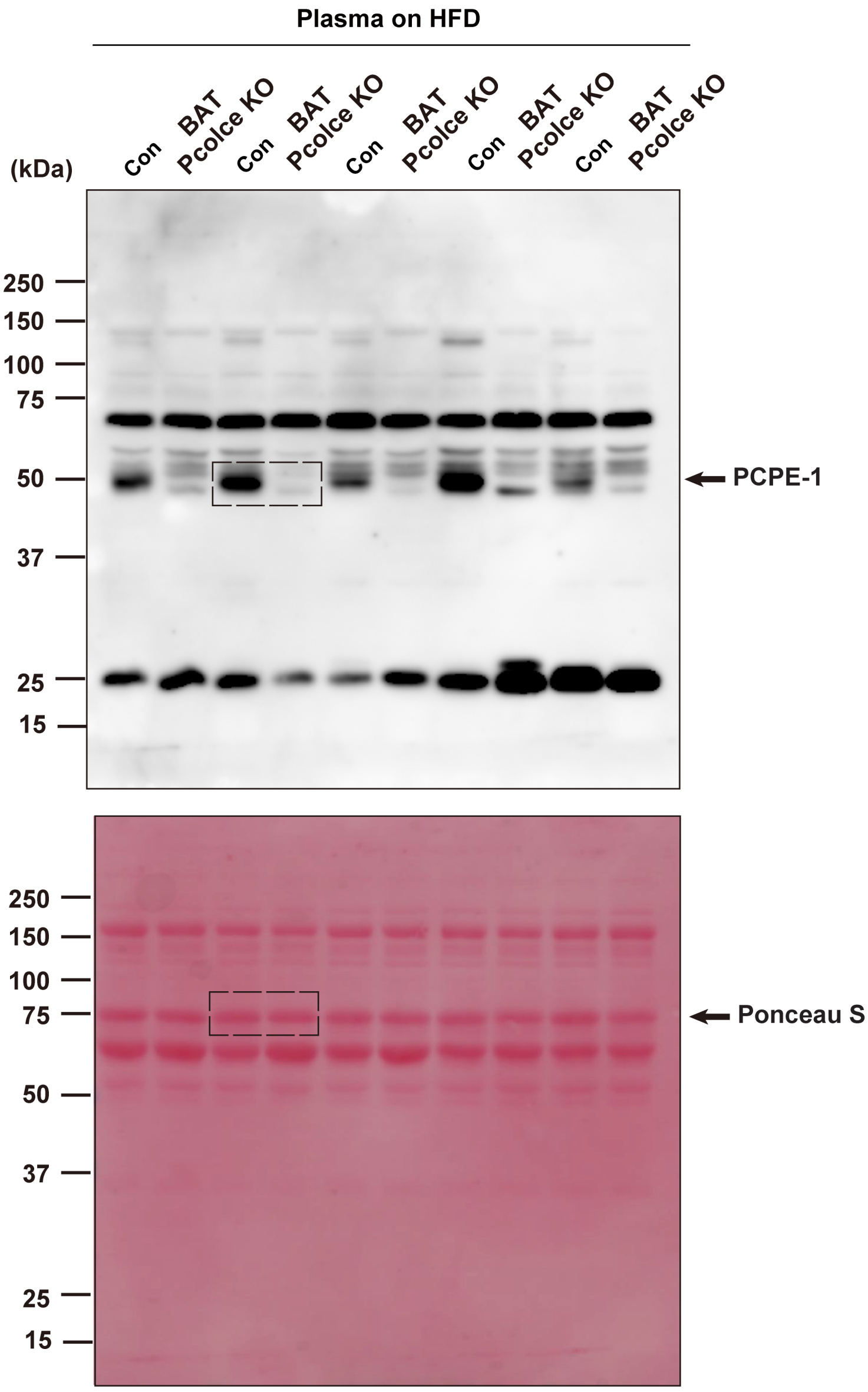

Supplement: Supplementary file 2 [file jciinsight-11-195508-s040.pdf]
